# Correction: Maternal vitamin D intake and BMI during pregnancy in relation to child’s growth and weight status from birth to 8 years: a large national cohort study

**DOI:** 10.1136/bmjopen-2021-048980corr1

**Published:** 2023-03-08

Amberntsson A, Papadopoulou E, Winkvist A, *et al*. Maternal vitamin D intake and BMI during pregnancy in relation to child’s growth and weight status from birth to 8 years: a large national cohort study. *BMJ Open* 2021;11:e048980. doi: 10.1136/bmjopen-2021-048980

The authors want to alert the readers on the updates done for tables 2-3 in the published version.

