# Supplementary material for: Maternal vitamin D intake and BMI during pregnancy in relation to child’s growth and weight status from birth to 8 years: a large national cohort study
Source: BMJ Open. 2021 Oct 1;11(10):e048980. doi: 10.1136/bmjopen-2021-048980 (PMC8488702; doi:10.1136/bmjopen-2021-048980)
Supplement: Supplementary data [file bmjopen-2021-048980supp001.pdf]

Supplementary table 1. Study population retention and anthropometric measures of the children at the 11 ages.

Intended age	Reported age	Weight (kg)		Response rate	Height (cm)		Response rate
	**Mean (SD)**	**N**	**Mean (SD)**	**%**	**N**	**Mean (SD)**	**%**
1.5 months	1.4 (0.2) months	56 625	5.0 (0.7)	84	45 745	57 (2.3)	67
3 months	3.1 (0.3) months	62 237	6.4 (0.8)	92	61 266	62 (2.5)	91
6 months	5.8 (0.5) months	60 515	7.9 (1.0)	89	60 224	68 (2.5)	89
8 months	8.0 (0.8) months	49 528	8.8 (1.0)	73	49 354	71 (2.7)	73
12 months	12.2 (0.6) months	49 609	9.9 (1.1)	73	49 686	76 (2.7)	73
15-18 months	16.0 (1.3) months	50 393	10.9 (1.2)	75	50 168	81 (3.0)	74
2 years	2.1 (0.2) years	25 195	13.0 (1.5)	37	25 346	89 (3.7)	37
3 years	3.0 (0.1) years	36 987	15.1 (1.8)	55	36 365	97 (3.8)	54
5 years	5.2 (0.3) years	30 288	20.0 (2.8)	45	31 671	113 (5.2)	47
7 years	7.1 (0.1) years	36 480	25.1 (4.0)	54	38 130	126 (5.4)	56
8 years	8.1 (0.1) years	23 123	28.2 (4.5)	34	24 611	132 (5.7)	36

Supplementary table 2. Cut-offs of overweight in children

Age (years)	Overweight cut-offs[a, b]		Prevalence (%)[c]	
	Boys	Girls	Boys	Girls
**3**	17.85	17.64	9.9	9.5
**5**	17.39	17.23	18.8	20.5
**8**	18.41	18.28	5.5	8.4

[a] Cole TJ, Lobstein T. Extended international (IOTF) body mass index cut-offs for thinness, overweight and obesity. Pediatr Obes. 2012;7(4):284-294. doi:10.1111/j.2047-6310.2012.00064.x

[b] All children with a body mass index equal to, or above, the cut-off are denoted as overweight

[c] Based on child´s body mass index calculated from the predicted anthropometric data.

Supplementary table 3. Associations between maternal vitamin D intake from foods alone, and from supplements alone, and predicted weight growth trajectories from 1 month to 2 years of age.

	**Infancy**			**Toddlerhood**		
	**1 month**	**3 months**	**6 months**	**12 months**	**18 months**	**2 years**
	**Beta (95% CI)**	**Beta (95% CI)**	**Beta (95% CI)**	**Beta (95% CI)**	**Beta (95% CI)**	**Beta (95% CI)**
**Maternal vitamin D intake from foods alone**						
All mothers n=66 840						
<2.5 µg						
2.5-4.9 µg	-5.8 (-14.6, 2.99)	-6.2 (-15.1, 2.7)	-6.7 (-16.1, 2.6)	-7.9 (-19.2, 3.5)	-9.0 (-23.1, 5.1)	-10.2 (-27.4, 7.1)
5-7.5 µg	-9.7 (-22.8, 3.5)	-7.1 (-20.4, 6.2)	-3.3 (-17.4, 10.7)	4.3 (-12.5, 21.1)	11.9 (-8.9, 32.7)	19.5 (-5.9, 45.0)
>7.5 µg	-12.0 (-34.8, 10.8)	-11.6 (-34.7, 11.4)	-11.0 (-35.3, 13.3)	-9.8 (-39.0, 19.3)	-8.7 (-44.7, 27.4)	-7.5 (-51.6, 36.6)
Mothers with normal weight [a] n=41 970						
<2.5 µg						
2.5-4.9 µg	-6.4 (-16.2, 3.4)	-7.0 (-16.9, 3.0)	-7.8 (-18.3, 2.7)	-9.3 (-22.0, 3.4)	-10.9 (-26.7, 4.9)	-12.5 (-31.9, 6.9)
5-7.5 µg	-7.3 (-21.7, 7.1)	-5.4 (-19.9, 9.2)	-2.5 (-17.8, 12.9)	3.3 (-15.1, 21.8)	9.1 (-13.8, 32.0)	14.9 (-13.1, 42.9)
>7.5 µg	-14.9 (-40.1, 10.3)	-14.8 (-40.3, 10.7)	-14.6 (-41.5, 12.3)	-14.1 (-46.5, 18.2)	-13.7 (-53.8, 26.4)	-13.2 (-62.3, 35.8)
Mothers with overweigh [b] n=20 080						
<2.5 µg						
2.5-4.9 µg	-4.3 (-17.5, 9.0)	-4.3 (-17.7, 9.2)	-4.3 (-18.5, 9.9)	-4.4 (-21.6, 12.8)	-4.5 (-25.9, 16.9)	-4.6 (-30.9, 21.7)
5-7.5 µg	-15.3 (-35.2, 4.6)	-11.3 (-31.4, 8.8)	-5.3 (-26.6, 15.9)	6.6 (-19.0, 32.3)	18.6 (-13.2, 50.4)	30.6 (-8.4, 69.6)
>7.5 µg	-5.1 (-39.1, 28.9)	-4.1 (-38.5, 30.3)	-2.6 (-38.9, 33.7)	0.3 (-43.5, 44.1)	3.3 (-51.1, 57.7)	6.2 (-60.4, 72.8)
**Maternal vitamin D intake from supplements alone**						
All mothers n=66 840						
<5 µg						
5-9.9 µg	-11.8 (-21.6, -2.1)	-11.4 (-21.3, -1.5)	-10.8 (-21.2, -0.3)	-9.5 (-22.1, 3.2)	-8.2 (-23.9, 7.6)	-6.9 (-26.2, 12.4)
10-15 µg	-4.0 (-17.4, 9.3)	-4.1 (-17.6, 9.4)	-4.1 (-18.4, 10.2)	-4.1 (-21.4, 13.2)	-4.2 (-25.7, 17.3)	-4.2 (-30.7, 22.2)
>15 µg	-8.5 (-21.5, 4.5)	-7.0 (-20.1, 6.2)	-4.6 (-18.5, 9.3)	0.1 (-16.8, 17.9)	4.8 (-16.2, 25.8)	9.5 (-16.2, 35.3)
Mothers with normal weight [a] n=41 970						
<5 µg						

5-9.9 µg	-10.6 (-21.4, 0.2)	-11.7 (-22.6, -0.7)	-13.3 (-24.9, -1.8)	-16.6 (-30.6, -2.6)	-19.9 (-37.4, -2.5)	-23.2 (-44.6, -1.8)
10-15 µg	-5.8 (-20.4, 8.7)	-7.1 (-21.9, 7.6)	-9.1 (-24.7, 6.5)	-13.0 (-31.9, 5.9)	-16.9 (-40.5, 6.6)	-20.9 (-49.7, 8.0)
>15 µg	-8.6 (-22.5, 5.4)	-8.2 (-22.4, 5.9)	-7.7 (-22.6, 7.3)	-6.6 (-24.7, 11.5)	-5.6 (-28.1, 17.0)	-4.5 (-32.2, 23.2)
Mothers with overweight [b] ***n=20 080***						
<5 µg						
5-9.9 µg	-14.9 (-29.8, 0.1)	-10.8 (-25.9, 4.3)	-4.7 (-20.7, 11.3)	7.5 (-11.8, 26.9)	20.0 (-4.4, 43.9)	32.0 (2.3, 61.6)
10-15 µg	0.2 (-21.6, 22.0)	3.2 (-18.8, 25.3)	7.8 (-15.5, 31.2)	17.0 (-11.3, 45.3)	26.1 (-9.2, 61.4)	35.2 (-8.1, 78.6)
>15 µg	-8.4 (-30.2, 13.4)	-4.0 (-26.0, 18.1)	2.7 (-20.6, 26.0)	16.1 (-12.2, 44.3)	29.4 (-5.8, 64.7)	42.8 (-0.4, 86.1)

Beta=Beta coefficient, CI=Confidence interval

Effect estimates derive from multilevel mixed effects linear regression model, adjusted for maternal education, parity, maternal milk and yoghurt intake, maternal fiber intake, child´s gender and birth weight. The model with all mothers was also adjusted for maternal pre-pregnancy BMI.

[a] Mothers with pre-pregnancy BMI 18.5-24.9 kg/m^2

[b] Mothers with pre-pregnancy BMI ≥25 kg/m^2

Supplementary table 4. Associations between maternal vitamin D intake from foods alone, and from supplements alone, and predicted weight growth trajectories from 3 years to 8 years of age.

	**Preschool age**		**School age**
	**3 years**	**5 years**	**8 years**
	**Beta (95% CI)**	**Beta (95% CI)**	**Beta (95% CI)**
**Maternal vitamin D intake from foods alone**			
All mothers n=66 840			
<5 µg			
5-9.9 µg	-12.4 (-36.7, 11.8)	-17.0 (-56.0, 22.0)	-23.8 (-85.6, 38.0)
10-15 µg	34.8 (-0.8, 70.3)	65.2 (8.1, 122)	111 (20.5, 201)
>15 µg	-5.1 (-66.8, 56.5)	-0.4 (-99.4, 98.6)	6.7 (-150, 163)
Mothers with normal weight [a] n=41 970			
<5 µg			
5-9.9 µg	-15.7 (-42.8, 11.5)	-22.0 (-65.8, 21.8)	-31.4 (-100, 37.9)
10-15 µg	26.4 (-12.7, 65.6)	49.6 (-13.4, 113)	84.3 (-15.3, 184)
>15 µg	-12.4 (-81.0, 56.3)	-10.6 (-121, 99.8)	-8.0 (-183, 167)
Mothers with overweight [b] n=20 080			
<5 µg			
5-9.9 µg	-4.8 (-41.7, 32.1)	-5.1 (-64.6, 54.2)	-5.7 (-99.8, 88.3)
10-15 µg	54.5 (-0.1, 109)	102.4 (14.5, 190)	174.3 (35.1, 313)
>15 µg	12.1 (-81.3, 106)	23.9 (-126, 174)	41.7 (-196, 280)
**Maternal vitamin D intake from supplements alone**			
All mothers n=66 840			
<5 µg			
5-9.9 µg	-4.3 (-31.4, 22.8)	0.9 (-42.7, 44.6)	8.7 (-60.5, 77.9)
10-15 µg	-4.3 (-41.4, 32.8)	-4.5 (-64.3, 55.2)	-4.8 (-99.5, 89.8)
>15 µg	18.9 (-17.2, 55.1)	37.8 (-20.5, 96.0)	66.0 (-26.3, 158)
Mothers with normal weight [a] n=41 970			
<5 µg			

5-9.9 µg	-29.8 (-59.9, 0.3)	-43.0 (-91.5, 5.4)	-62.8 (-140, 13.9)
10-15 µg	-28.7 (-69.3, 11.9)	-44.4 (-110, 21.0)	-67.9 (-171, 35.6)
>15 µg	-2.4 (-41.2, 36.5)	1.9 (-60.7, 64.5)	8.2 (-90.9, 107)
Mothers with overweight [b] ***n=20 080***			
<5 µg			
5-9.9 µg	56.4 (14.8, 98.0)	105 (38.2, 172)	179 (72.3, 285)
10-15 µg	53.5 (-7.3, 114)	90.1 (-7.9, 188)	145 (-10.3, 300)
>15 µg	69.5 (8.8, 130)	123 (25.1, 221)	203 (48.2, 358)

Beta=Beta coefficient, CI=Confidence interval

Effect estimates derive from multilevel mixed effects linear regression model, adjusted for maternal education, parity, maternal milk and yoghurt intake, maternal fiber intake, child´s gender and birth weight. The model with all mothers was also adjusted for maternal pre-pregnancy BMI.

[a] Mothers with pre-pregnancy BMI 18.5-24.9 kg/m^2

[b] Mothers with pre-pregnancy BMI ≥25 kg/m^2

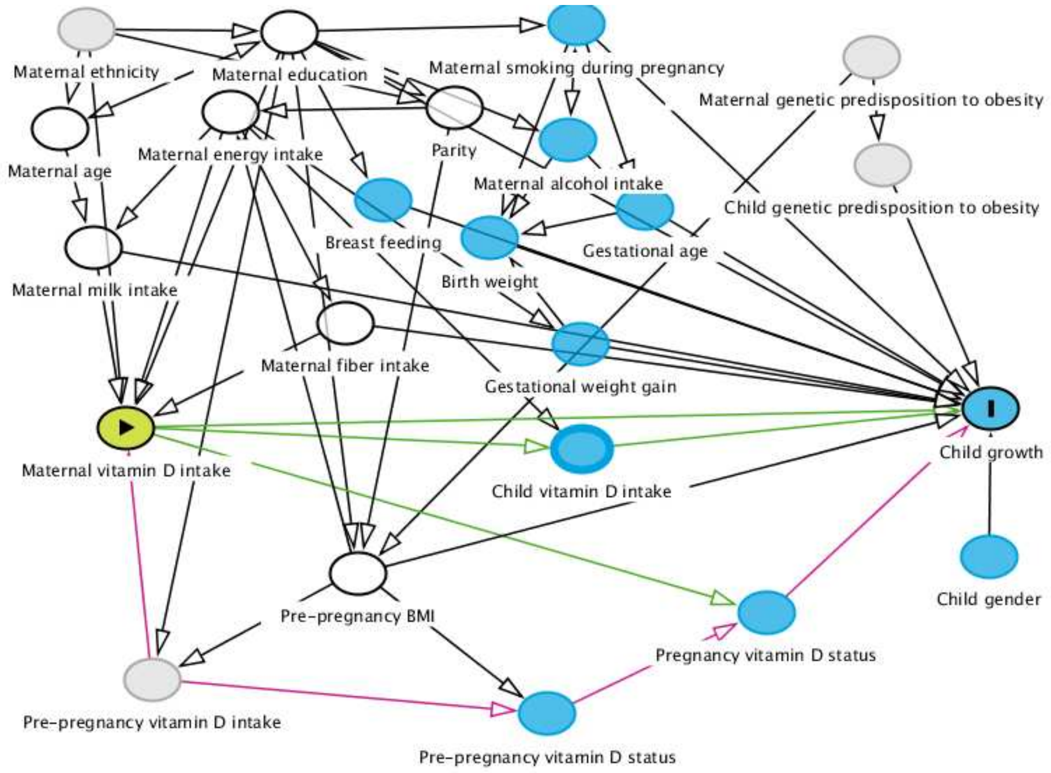

Supplementary figure 1. Directed acyclic graph on the suggested association between, exposure, outcome and covariates [a]. The graph is made using the software DAGitty[b].

Legends: White circle – ancestor of exposure and outcome, adjusted variable; blue circle – ancestor of outcome; grey circle – unobserved variable; black arrow – direct causal effects; green arrow – causal path; pink arrow – biasing path.

[a] Textor J, van der Zander B, Gilthorpe M. S, Liskiewicz M, Ellison G. T. Robust causal inference using directed acyclic graphs: the R package 'dagitty'. International Journal of Epidemiology 45(6):1887-1894, 2016.

[b] (www.dagitty.net/dags.html).

Supplementary table 5. Adjusted change in siblings' weights and heights from 1 month to 2 years, associated with an increase in maternal total vitamin D intake within sibships.

	**Infancy**			**Toddlerhood**		
	**1 month**	**3 months**	**6 months**	**12 months**	**18 months**	**2 years**
**Siblings in the higher maternal vitamin D category**	**Beta (95% CI)**	**Beta (95% CI)**	**Beta (95% CI)**	**Beta (95% CI)**	**Beta (95% CI)**	**Beta (95% CI)**
Weight (g)						
All mothers (n=1216)	22.0 (-28.0, 71.9)	20.4 (-30.0, 70.9)	18.2 (-34.3, 70.7)	13.7 (-46.6, 74.0)	9.2 (-62.5, 80.8)	4.6 (-80.6, 89.8)
Mothers with normal weight [a] (n=837)	3.2 (-56.6, 63.0)	2.0 (-58.2, 62.2)	0.3 (-61.7, 62,2)	-3.3 (-72.8, 66.2)	-6.8 (-87.8, 74.2)	-10.4 (-105, 84.6)
Mothers with overweight [b] (n=344)	31.3 (-57.7, 120)	29.6 (-59.9, 119)	27.0 (-65.1, 119)	21.9 (-81.4, 125)	16.9 (-104, 137)	11.8 (129, 153)
Height (cm)						
All mothers (n=1216)	0.07 (-0.15, 0.29)	0.07 (-0.14, 0.29)	0.07 (-0.14, 0.29)	0.08 (-0.12, 0.28)	0.08 (-0.11, 0.27)	0.08 (-0.11, 0.27)
Mothers with normal weight [a] (n=837)	0.02 (-0.22, 0.27)	0.02 (-0.22, 0.27)	0.03 (-0.21, 0.26)	0.04 (-0.19, 0.26)	0.04 (-0.17, 0.26)	0.05 (-0.16, 0.26)
Mothers with overweight [b] (n=344)	0.11 (-0.26, 0.47)	0.10 (-0.25, 0.46)	0.10 (-0.25, 0.45)	0.09 (-0.24, 0.42)	0.08 (-0.24, 0.40)	0.07 (-0.24, 0.39)

Beta=Beta coefficient, CI=Confidence interval

Effect estimates derived by multilevel mixed effects linear regression model, adjusted for maternal education, parity, maternal milk and yoghurt intake, maternal fiber intake, child´s gender and birth weight. The model with all mothers was also adjusted for maternal pre-pregnancy BMI. The siblings in the lower maternal vitamin D category was used as reference in separate analyses of overall (n=1211), mothers with normal weight (n=845) and with overweight (n=320).

[a] Mothers with pre-pregnancy BMI 18.5-24.9 kg/m^2

[b] Mothers with pre-pregnancy BMI ≥25 kg/m^2

Supplementary table 6. Adjusted changes in siblings' weights and heights from 3 years to 8 years of age, associated with an increase in maternal vitamin D intake within sibships.

	**Preschool age**		**School age**
	**3 years**	**5 years**	**8 years**
**Sibling in the higher maternal vitamin D category**	**Beta (95% CI)**	**Beta (95% CI)**	**Beta (95% CI)**
Weight (in g)			
All mothers (n=1216)	-4.4 (-120, 111)	-22.5 (-204, 159)	-49.6 (-334, 235)
Mothers with normal weight [a] (n=837)	-17.5 (-145, 110)	-31.7 (-230, 166)	-53.0 (-363, 257)
Mothers with overweight [b] (n=344)	1.6 (-187, 191)	-18.7 (-313, 276)	-49.2 (-510, 411)
Height (in cm)			
All mothers (n=1216)	0.09 (-0.10, 0.26)	0.10 (-0.12, 0.31)	0.11 (-0.20, 0.42)
Mothers with normal weight [a] (n=837)	0.07 (-0.14, 0.28)	0.10 (-0.14, 0.34)	0.15 (-0.19, 0.49)
Mothers with overweight [b] (n=344)	0.05 (-0.26, 0.36)	0.01 (-0.34, 0.37)	-0.04 (-0.55, 0.46)

Beta=Beta coefficient, CI=Confidence interval

Effect estimates derived by multilevel mixed effects linear regression model, adjusted for maternal education, parity, maternal milk and yoghurt intake, maternal fiber intake, child´s gender and birth weight. The model with all mothers was also adjusted for maternal pre-pregnancy BMI. The siblings in the lower maternal vitamin D category was used as reference in separate analyses of overall (n=1211), mothers with normal weight (n=845) and with overweight (n=320).

[a] Mothers with pre-pregnancy BMI 18.5-24.9 kg/m^2

[b] Mothers with pre-pregnancy BMI ≥25 kg/m^2

Supplementary table 7. Adjusted changes in siblings' risk of overweight at 3, 5 and 8 years, associated with an increase in maternal total vitamin D intake within sibships.

	**Risk of childhood overweight [a]**
	**OR (95% CI)**
**Sibling in the higher maternal vitamin D category**	
All mothers (n=1216)	
3 years	0.97 (0.75, 1.26)
5 years	0.92 (0.76, 1.12)
8 years	1.03 (0.80, 1.32)
Mothers with normal weight [b] (n=837)	
3 years	0.89 (0.62, 1.28)
5 years	0.92 (0.71, 1.20)
8 years	0.99 (0.70, 1.39)
Mothers with overweight [c] (n=344)	
3 years	1.04 (0.69, 1.57)
5 years	0.90 (0.64, 1.24)
8 years	1.03 (0.68, 1.57)

OR=Odds Ratio, CI=Confidence interval

Effect estimates derived by multilevel mixed effects linear regression model, adjusted for maternal education, parity, maternal milk and yoghurt intake, maternal fiber intake, child´s gender and birth weight. The model with all mothers was also adjusted for maternal pre-pregnancy BMI. The siblings in the lower maternal vitamin D category was used as reference in separate analyses of overall (n=1211), mothers with normal weight (n=845) and with overweight (n=320).

[a] Overweight, including obesity, defined by Cole TJ, Lobstein T. Extended international (IOTF) body mass index cut-offs for thinness, overweight and obesity. Pediatr Obes. 2012;7(4):284-294. doi:10.1111/j.2047-6310.2012.00064.x

[b] Mothers with pre-pregnancy BMI 18.5-24.9 kg/m^2

[c] Mothers with pre-pregnancy BMI ≥25 kg/m^2

Supplementary table 8. Associations between total maternal vitamin D intake and predicted weight growth velocities and height growth velocities from 1 month to 2 years of age.

	**Infancy**			**Toddlerhood**		
**Maternal vitamin D intake**	**1 month**	**3 months**	**6 months**	**12 months**	**18 months**	**2 years**
	**Beta (95% CI)**	**Beta (95% CI)**	**Beta (95% CI)**	**Beta (95% CI)**	**Beta (95% CI)**	**Beta (95% CI)**
All mothers n=66 840						
Weight growth velocities (g/month)						
<5 µg						
5-9.9 µg	-0.13 (-2.11, 1.86)	-0.11 (-2.04, 1.81)	-0.10 (-1.94, 1.75)	-0.06 (-1.75, 1.63)	-0.03 (-1.60, 1.54)	0.003 (-1.48, 1.49)
10-15 µg	-0.51 (-2.96, 1.94)	-0.52 (-2.89, 1.86)	-0.53 (-2.80, 1.74)	-0.55 (-2.63, 1.53)	-0.57 (-2.51, 1.36)	-0.60 (-2.43, 1.23)
>15 µg	-0.25 (-2.70, 2.20)	-0.24 (-2.62, 2.14)	-0.24 (-2.51, 2.04)	-0.22 (-2.31, 1.87)	-0.20 (-2.14, 1.73)	-0.19 (-2.03, 1.65)
Height growth velocities (cm/month)						
<5 µg						
5-9.9 µg	$4x10^{-5}$ (-0.004, 0.004)	$4x10^{-5}$ (-0.004, 0.004)	$3x10^{-5}$ (-0.004, 0.004)	$3x10^{-5}$ (-0.004, 0.004)	$2x10^{-5}$ (-0.003, 0.003)	$2x10^{-5}$ (-0.003, 0.003)
10-15 µg	$7x10^{-5}$ (-0.005, 0.005)	$3x10^{-5}$ (-0.005, 0.005)	$-3x10^{-5}$ (-0.005, 0.005)	$-2x10^{-4}$ (-0.005, 0.004)	$-3x10^{-4}$ (-0.004, 0.004)	$-4x10^{-4}$ (-0.004, 0.003)
>15 µg	$7x10^{-4}$ (-0.004, 0.006)	$7x10^{-4}$ (-0.004, 0.006)	$7x10^{-4}$ (-0.004, 0.005)	$6x10^{-4}$ (-0.004, 0.005)	$5x10^{-4}$ (-0.004, 0.005)	$5x10^{-4}$ (-0.003, 0.004)
Mothers with normal weight [a] n=41 970						
Weight growth velocities (g/month)						
<5 µg						
5-9.9 µg	-1.03 (-3.28, 1.22)	-1.00 (-3.18, 1.18)	-0.96 (-3.04, 1.12)	-0.88 (-2.79, 1.02)	-0.80 (-2.57, 0.96)	-0.73 (-2.40, 0.95)
10-15 µg	-1.97 (-4.69, 0.75)	-1.95 (-4.59, 0.69)	-1.92 (-4.44, 0.60)	-1.86 (-4.17, 0.45)	-1.80 (-3.94, 0.35)	-1.74 (-3.77, 0.29)
>15 µg	-1.17 (-3.86, 1.53)	-1.14 (-3.76, 1.47)	-1.11 (-3.60, 1.38)	-1.05 (-3.34, 1.24)	-0.99 (-3.11, 1.13)	-0.93 (-2.94, 1.08)
Height growth velocities (cm/month)						
<5 µg						
5-9.9 µg	$-8x10^{-5}$ (-0.005, 0.005)	$-8x10^{-5}$ (-0.005, 0.005)	$-8x10^{-5}$ (-0.004, 0.004)	$-8x10^{-5}$ (-0.004, 0.004)	$-8x10^{-5}$ (-0.004, 0.004)	$-8x10^{-5}$ (-0.004, 0.003)
10-15 µg	$-4x10^{-4}$ (-0.006, 0.005)	$-5x10^{-4}$ (-0.006, 0.005)	$-5x10^{-4}$ (-0.006, 0.005)	$-6x10^{-4}$ (-0.006, 0.004)	$-8x10^{-4}$ (-0.005, 0.004)	$-9x10^{-4}$ (-0.005, 0.003)
>15 µg	$7x10^{-4}$ (-0.005, 0.006)	$6x10^{-4}$ (-0.005, 0.006)	$6x10^{-4}$ (-0.005, 0.006)	$-6x10^{-4}$ (-0.004, 0.005)	$-5x10^{-4}$ (-0.004, 0.005)	$-4x10^{-4}$ (-0.004, 0.005)

Mothers with overweight [b] ***n=20 080***						
Weight growth velocities (g/month)						
<5 µg						
5-9.9 µg	1.17 (-1.87, 1.22)	1.18 (-1.68, 4.03)	1.19 (-1.54, 3.92)	1.22 (-1.28, 3.72)	1.25 (-1.07, 3.56)	1.28 (-0.91, 3.47)
10-15 µg	2.23 (-1.54, 6.00)	2.20 (-1.46, 5.85)	2.15 (-1.35, 5.64)	2.05 (-1.15, 5.25)	1.95 (-1.02, 4.92)	1.85 (-0.95, 4.66)
>15 µg	0.68 (-3.29, 4.65)	0.72 (-3.13, 4.57)	0.78 (-2.90, 4.45)	0.89 (-2.48, 4.26)	1.01 (-2.11, 4.13)	1.13 (-1.83, 4.08)
Height growth velocities (cm/month)						
<5 µg						
5-9.9 µg	$-3x10^{-4}$ (-0.006, 0.006)	$-2x10^{-4}$ (-0.006, 0.006)	$-2x10^{-4}$ (-0.006, 0.006)	$-1x10^{-4}$ (-0.005, 0.005)	$4x10^{-5}$ (-0.005, 0.005)	$4x10^{-5}$ (-0.005, 0.005)
10-15 µg	$6x10^{-4}$ (-0.007, 0.008)	$6x10^{-4}$ (-0.007, 0.008)	$6x10^{-4}$ (-0.007, 0.008)	$6x10^{-4}$ (-0.006, 0.007)	$5x10^{-4}$ (-0.006, 0.007)	$6x10^{-4}$ (-0.005, 0.006)
>15 µg	$-6x10^{-4}$ (-0.009, 0.008)	$-5x10^{-4}$ (-0.009, 0.008)	$-4x10^{-4}$ (-0.008, 0.007)	$-3x10^{-4}$ (-0.007, 0.007)	$-1x10^{-4}$ (-0.007, 0.006)	$-5x10^{-5}$ (-0.006, 0.006)
Beta=Beta coefficient, CI=Confidence interval						
Effect estimates derive from multilevel mixed effects linear regression model, adjusted for maternal education, parity, maternal milk and yoghurt intake, maternal fiber intake, child´s gender and birth weight. The model with all mothers was also adjusted for maternal pre-pregnancy BMI.						
[a] Mothers with pre-pregnancy BMI 18.5-24.9 kg/m^2						
[b] Mothers with pre-pregnancy BMI ≥25 kg/m^2						

BMJ Publishing Group Limited (BMJ) disclaims all liability and responsibility arising from any reliance placed on this supplemental material which has been supplied by the author(s)

Supplementary table 9. Associations between total maternal vitamin D intake and predicted weight growth velocities and height growth velocities from 3 years to 8 years of age.

	**Preschool age**		**School age**
	**3 years**	**5 years**	**8 years**
**Maternal vitamin D intake**	**Beta (95% CI)**	**Beta (95% CI)**	**Beta (95% CI)**
All mothers ***n=66 840***			
Weight growth velocities (g/month)			
<5 µg			
5-9.9 µg	0.07 (-1.39, 1.52)	0.20 (-1.71, 2.12)	0.40 (-2.80, 3.61)
10-15 µg	-0.64 (-2.44, 1.15)	-0.73 (-3.09, 1.63)	-0.86 (-4.81, 3.08)
>15 µg	-0.16 (-1.96, 1.64)	-0.10 (-2.46, 2.26)	-0.007 (-3.95, 3.93)
Height growth velocities (cm/month)			
<5 µg			
5-9.9 µg	$4x10^{-6}$ (-0.003, 0.003)	$-4x10^{-5}$ (-0.004, 0.004)	$-5x10^{-5}$ (-0.007, 0.007)
10-15 µg	$-7x10^{-4}$ (-0.004, 0.003)	-0.001 (-0.006, 0.004)	-0.002 (-0.010, 0.006)
>15 µg	$3x10^{-4}$ (-0.003, 0.004)	$9x10^{-5}$ (-0.005, 0.005)	$-3x10^{-4}$ (-0.009, 0.008)
Mothers with normal weight [a] ***n=41 970***			
Weight growth velocities (g/month)			
<5 µg			
5-9.9 µg	-0.57 (-2.21, 1.07)	-0.26 (-2.42, 1.91)	-0.21 (-3,42, 3.84)
10-15 µg	-1.61 (-3.60, 0.38)	-1.37 (-3.99, 1.25)	-1.00 (-5.39, 3.39)
>15 µg	-0.80 (-2.78, 1.17)	-0.56 (-3.15, 2.04)	-0.18 (-4.52, 4.15)
Height growth velocities (cm/month)			
<5 µg			
5-9.9 µg	$-8x10^{-5}$ (-0.004, 0.003)	$-7x10^{-5}$ (-0.005, 0.004)	$-7x10^{-5}$ (-0.008, 0.008)
10-15 µg	-0.001 (-0.005, 0.003)	-0.002 (-0.007, 0.004)	-0.002 (-0.011, 0.007)
>15 µg	$3x10^{-4}$ (-0.004, 0.004)	$1x10^{-4}$ (-0.005, 0.006)	$-2x10^{-4}$ (-0.009, 0.009)

**Mothers with overweight [b]**<br>**n=20 080**			
Weight growth velocities (g/month)			
<5 µg			
5-9.9 µg	1.33 (-0.81, 3.48)	1.45 (-1.39, 4.29)	1.62 (-3.14, 6.38)
10-15 µg	1.66 (-1.10, 4.41)	1.26 (-2.37, 4.89)	0.67 (-5.42, 6.77)
>15 µg	1.36 (-1.54, 4.26)	1.82 (-2.00, 5.64)	2.52 (-3.88, 8.92)
Height growth velocities (cm/month)			
<5 µg			
5-9.9 µg	$2x10^{-4}$ (-0.004, 0.005)	$5x10^{-4}$ (-0.005, 0.006)	0.001 (-0.009, 0.011)
10-15 µg	$5x10^{-4}$ (-0.005, 0.006)	$4x10^{-4}$ (-0.007, 0.008)	$2x10^{-4}$ (-0.013, 0.013)
>15 µg	$4x10^{-4}$ (-0.006, 0.006)	0.001 (-0.007, 0.009)	0.002 (-0.011, 0.015)

Beta=Beta coefficient, CI=Confidence interval

Effect estimates derive from multilevel mixed effects linear regression model, adjusted for maternal education, parity, maternal milk and yoghurt intake, maternal fiber intake, child´s gender and birth weight. The model with all mothers was also adjusted for maternal pre-pregnancy BMI.

[a] Mothers with pre-pregnancy BMI 18.5-24.9 kg/m^2

[b] Mothers with pre-pregnancy BMI ≥25 kg/m^2

Supplementary table 10. Associations (OR and 95% CI) between total maternal vitamin D intake and risk of childhood overweight at 3 years, 5 years and 8 years, using reported anthropometrics.

	**Risk of childhood overweight [a]**
**Maternal vitamin D intake**	**OR (95% CI)**
All mothers	
**3 years *(n=34 438)***	
<5 µg	
5-9.9 µg	1.00 (0.93, 1.08)
10-15 µg	0.96 (0.87, 1.06)
>15 µg	0.89 (0.81, 0.98)
**5 years (n=29 301)**	
<5 µg	
5-9.9 µg	0.97 (0.88, 1.06)
10-15 µg	0.97 (0.87, 1.08)
>15 µg	0.95 (0.85, 1.06)
**8 years (n=22 447)**	
<5 µg	
5-9.9 µg	1.10 (1.00, 1.20)
10-15 µg	1.05 (0.94, 1.17)
>15 µg	1.06 (0.95, 1.18)
Mothers with normal weight [b]	
**3 years *(n=22 863)***	
<5 µg	
5-9.9 µg	0.98 (0.89, 1.08)
10-15 µg	0.95 (0.84, 1.07)
>15 µg	0.87 (0.77, 0.98)
**5 years (n=19 767)**	
<5 µg	
5-9.9 µg	0.98 (0.87, 1.10)
10-15 µg	0.94 (0.81, 1.08)
>15 µg	0.90 (0.78, 1.04)
**8 years (n=15 171)**	
<5 µg	
5-9.9 µg	1.03 (0.91, 1.16)
10-15 µg	0.96 (0.83, 1.11)
>15 µg	1.02 (0.89, 1.18)
Mothers with overweight [c]	
**3 years (n=10 633)**	
<5 µg	
5-9.9 µg	1.02 (0.90, 1.15)
10-15 µg	0.96 (0.82, 1.13)
>15 µg	0.88 (0.74, 1.04)
**5 years (n=8709)**	
<5 µg	
5-9.9 µg	0.94 (0.82, 1.08)

10-15 µg	1.02 (0.86, 1.21)
>15 µg	0.98 (0.82, 1.17)
**8 years (n=6661)**	
<5 µg	
5-9.9 µg	1.22 (1.06, 1.40)
10-15 µg	1.19 (1.00, 1.42)
>15 µg	1.11 (0.93, 1.33)

OR=Odds Ratio, CI=Confidence interval

Effect estimates derived by logistic regression, adjusted for maternal education, parity, maternal milk and yoghurt intake, maternal fiber intake and child´s gender. The model with all mothers was also adjusted for maternal pre-pregnancy BMI.

[a] Overweight, including obesity, defined by Cole TJ, Lobstein T. Extended international (IOTF) body mass index cut-offs for thinness, overweight and obesity. Pediatr Obes. 2012;7(4):284-294. doi:10.1111/j.2047-6310.2012.00064.x

[b] Mothers with pre-pregnancy BMI 18.5-24.9 kg/m^2

[c] Mothers with pre-pregnancy BMI ≥25 kg/m^2
