# Supplementary material for: Maternal vitamin D intake and BMI during pregnancy in relation to child’s growth and weight status from birth to 8 years: a large national cohort study
Source: BMJ Open. 2021 Oct 1;11(10):e048980. doi: 10.1136/bmjopen-2021-048980 (PMC8488702; doi:10.1136/bmjopen-2021-048980)
Supplement: Author's manuscript [file bmjopen-2021-048980.draft_revisions.pdf]

BMJ Open is committed to open peer review. As part of this commitment we make the peer review history of every article we publish publicly available.

When an article is published we post the peer reviewers' comments and the authors' responses online. We also post the versions of the paper that were used during peer review. These are the versions that the peer review comments apply to.

The versions of the paper that follow are the versions that were submitted during the peer review process. They are not the versions of record or the final published versions. They should not be cited or distributed as the published version of this manuscript.

BMJ Open is an open access journal and the full, final, typeset and author-corrected version of record of the manuscript is available on our site with no access controls, subscription charges or pay-per-view fees (http://bmjopen.bmj.com).

If you have any questions on BMJ Open's open peer review process please email info.bmjopen@bmj.com

BMJ Open

Maternal vitamin D intake and BMI during pregnancy and child growth and overweight from birth to 8 years

Journal:	*BMJ Open*
Manuscript ID	bmjopen-2021-048980
Article Type:	Original research
Date Submitted by the Author:	12-Jan-2021
Complete List of Authors:	Amberntsson, Anna; University of Gothenburg, Institute of Medicine<br>Papadopoulou, Eleni; Norwegian Institute of Public Health<br>Winkvist, Anna; University of Gothenburg, Institute of Medicine<br>Lissner, Lauren; University of Gothenburg, Institute of Medicine<br>Meltzer, Helle Margrete; Norwegian Institute of Public Health<br>Brantsaeter, Anne Lise; Norwegian Institute of Public Health<br>Augustin, Hanna; University of Gothenburg, Institute of Medicine
Keywords:	EPIDEMIOLOGY, NUTRITION & DIETETICS, PREVENTIVE MEDICINE, PUBLIC HEALTH, SOCIAL MEDICINE

SCHOLARONE™
Manuscripts

I, the Submitting Author has the right to grant and does grant on behalf of all authors of the Work (as defined in the below author licence), an exclusive licence and/or a non-exclusive licence for contributions from authors who are: i) UK Crown employees; ii) where BMJ has agreed a CC-BY licence shall apply, and/or iii) in accordance with the terms applicable for US Federal Government officers or employees acting as part of their official duties; on a worldwide, perpetual, irrevocable, royalty-free basis to BMJ Publishing Group Ltd ("BMJ") its licensees and where the relevant Journal is co-owned by BMJ to the co-owners of the Journal, to publish the Work in this journal and any other BMJ products and to exploit all rights, as set out in our licence.

The Submitting Author accepts and understands that any supply made under these terms is made by BMJ to the Submitting Author unless you are acting as an employee on behalf of your employer or a postgraduate student of an affiliated institution which is paying any applicable article publishing charge ("APC") for Open Access articles. Where the Submitting Author wishes to make the Work available on an Open Access basis (and intends to pay the relevant APC), the terms of reuse of such Open Access shall be governed by a Creative Commons licence – details of these licences and which Creative Commons licence will apply to this Work are set out in our licence referred to above.

Other than as permitted in any relevant BMJ Author's Self Archiving Policies, I confirm this Work has not been accepted for publication elsewhere, is not being considered for publication elsewhere and does not duplicate material already published. I confirm all authors consent to publication of this Work and authorise the granting of this licence.

For peer review only

Maternal vitamin D intake and BMI during pregnancy and child growth and overweight from birth to 8 years

Anna Amberntsson[1], Eleni Papadopoulou[2], Anna Winkvist[1], Lauren Lissner[1], Helle Margrete Meltzer[2], Anne-Lise Brantsæter[2], Hanna Augustin[1]

1. Institute of Medicine, Sahlgrenska Academy, University of Gothenburg

2. Norwegian Institute of Public Health, Oslo, Norway

Corresponding author: Anna Amberntsson; anna.amberntsson@gu.se

For peer review only

Abstract

Objectives: To examine the associations between maternal vitamin D intake and childhood growth and risk of overweight up to 8 years. We further examined the effect modification by maternal pre-pregnancy BMI.

Design: Prospective population-based pregnancy cohort study

Setting: The Norwegian Mother, Father and Child Cohort Study.

Participants: In total, 58 724 mothers and 66 840 singleton children, with information on maternal vitamin D intake during pregnancy and minimum one postnatal anthropometric measurement.

Outcome measures: Predicted weight and height growth trajectories and velocities from 1 month to 8 years, rapid growth during infancy and toddlerhood, and risk of overweight in pre-school and school age.

Results: Overall, maternal vitamin D intake was associated with lower weight trajectory, lower odds of rapid weight growth and higher odds of childhood overweight. In children of mothers with pre-pregnancy normal weight, maternal vitamin D intake was negatively associated with weight trajectory and lower odds ratio of a rapid weight growth during the first year, compared to reference (<5µg/day). Children of mothers with normal weight, with maternal vitamin D intakes of 10-15 and >15 µg/day, also had 0.86 (95% CI 0.77, 0.97) and 0.88 (95% CI 0.79, 0.99) lower odds for overweight at 3 years, compared to reference. In contrast, in children of mothers with pre-pregnancy overweight (BMI ≥25 kg/m^2), vitamin D intake was positively associated with weight trajectory. Children of mothers with overweight, with maternal vitamin D intake of 5-9.9 µg/day, also had (1.09 (95% CI 1.01, 1.18) and 1.12 (95% CI 1.02, 1.23)) higher odds for overweight at 5 years and 8 years, compared to reference.

Conclusions: Maternal vitamin D intake affects postnatal growth and is inversely associated with childhood overweight in children of mothers with normal weight. Associations between maternal vitamin D intake and child growth and risk of overweight varied by pre-pregnancy BMI.

For peer review only

Article Summary

- To our knowledge, this is the first study to investigate the association between maternal vitamin D intake during pregnancy and postnatal growth
- The large, nationwide study population is a major strength
- Maternal vitamin D intake was estimated from both foods and supplements by a validated food frequency questionnaire
- A limitation is self-reported maternal data and parental-reported growth data of the child

Key words: The Norwegian Mother, Father and Child Cohort Study, weight, height

For peer review only

Introduction

Vitamin D has received much attention over the last few decades and its role in human physiology seems to extend beyond bone health [1]. The human skin can synthesize a precursor of the vitamin when exposed to ultra violet-B rays of wavelength 290-315 nm [2]. At northern latitudes, this only occurs from April to October. Therefore, intake of vitamin D from foods and supplements becomes important during winter months in many countries, including the Nordics [3]. Vitamin D occurs naturally in a few foods, such as fatty fish, but is presently added to many fortified foods in the form of vitamin D_3[2]. The more uncommon form vitamin D_2 is only present in some plants and mushrooms [4]. Synthesized or ingested vitamin D (vitamin D_3 and vitamin D_2) is hydroxylated in the liver to 25-hydroxyvitamin D (25OHD), an established blood biomarker of vitamin D status. Further, 25OHD undergoes another hydroxylation primarily in the kidneys to form the physiologically active 1,25-dihydroxyvitamin D (1,25OH_2D) [4]. The circulating concentration of 25OHD has shown to be inversely associated with BMI, suggesting that individuals with obesity have a decreased bioavailability of vitamin D [5].

Worldwide, the prevalence of vitamin D insufficiency (here defined as 25OHD <50 nmol/L) is high among pregnant women [6]. In some regions, such as South-East Asia and Western Pacific, the prevalence of vitamin D insufficiency were as high as 80% [6]. In contrast, in Europe and Eastern Mediterranean, the prevalence of vitamin D insufficiency were 57% and 46%, respectively [6]. In the Nordic countries, up to 28% of the pregnant fair-skinned European women have vitamin D insufficiency, but among non-fair skinned European women living in the Nordic countries, the prevalence is rising between 69% and 82% [7-9]. Therefore, on a population level, vitamin D insufficiency and deficiency in pregnancy are a relevant public health issue affecting the future generations. Pregnancy is a critical period where fetal exposure to poor conditions can affect later outcomes in the offspring [10, 11]. Maternal vitamin D status, assessed as serum or plasma 25OHD concentrations, has been suggested as a potential modifiable early-life risk factor for development of childhood obesity [12]. Early rapid growth can increase the risk for childhood obesity, and both conditions are associated with several comorbidities in both childhood and adulthood, and is likely to follow the child through life [13-16]. In 2018, globally the prevalence of children with overweight and obesity under 5 years of age had risen to 40 million [17].

For peer review only

Associations have been found between low maternal vitamin D status and an increased risk of the child being born small for gestational age and with a lower birth weight [18], as well as accelerated growth in weight and height during infancy [19]. Maternal vitamin D status has also been inversely associated with childhood adiposity at 4 and 6 years [20] and childhood insulin resistance at 9.5 years [21]. Further, associations of an increased risk of childhood overweight at 12 months of age was seen in children of mothers with low vitamin D status during pregnancy [22, 23]. However, some studies have found opposite results, i.e. that poor maternal vitamin D status was associated with reduced child growth [24], while others have reported null associations with childhood adiposity [21, 25, 26] and growth [22, 25]. Even though the vitamin D intake from food and supplements is a determinant of vitamin D status [8], the role of maternal vitamin D intake during pregnancy has been less studied, and the limited evidence regarding fetal growth is discordant [27, 28]. Associations between maternal vitamin D intake in pregnancy and postnatal growth and overweight have not yet been explored. Since vitamin D intake affects vitamin D status [4], and due to the decreased bioavailability of vitamin D in obesity (due to volumetric dilution of 25OHD [5]), it is possible that such an association could be modified by maternal pre-pregnancy BMI.

Our objectives were to assess the associations between maternal vitamin D intake during pregnancy and childhood weight and height growth trajectories and velocities, rapid growth and risk of overweight up to 8 years, in a large, prospective pregnancy cohort study. Further, we aimed to examine maternal pre-pregnancy BMI as an effect modifier.

Method

Study population

Our study draws resources from the Norwegian Mother, Father and Child Cohort Study (MoBa), a population-based pregnancy cohort study conducted by the Norwegian Institute of Public Health [29]. Participants were recruited from all over Norway during 1999-2008 and invited at the time of their first ultrasound scanning. The women consented to participation in 41% of the pregnancies. The study now includes 95 200 mothers, 75 200 fathers and 114 500 children. The current study is based on version 12 of the quality-assured data files released for research in January 2019. The establishment of MoBa and initial data collection was based on a license from the Norwegian Data Protection Agency and approval from The Regional Committees for Medical and Health Research Ethics. The MoBa cohort is now based on regulations related to the Norwegian Health Registry Act. The current study was

For peer review only

approved by The Regional Committees for Medical and Health Research Ethics (2019/770 REK Nord). The were no involvement from members of the public in the design, or conduct, or reporting, or dissemination plans of the research.

Questionnaires were sent out to the participating women in pregnancy week 15, week 22 and week 30. The questionnaires in week 15 and 30 requested background information, such as education and parity, whereas the questionnaire in week 22 was a food frequency questionnaire (FFQ). After delivery, follow-up of the participants by questionnaires was conducted at six different time points; 6 months, 18 months, 36 months, 5 years, 7 years and 8 years [29, 30]. The MoBa database is linked to the Medical Birth Registry of Norway (MBRN), which is a national health registry containing information about all births in Norway [31].

Exclusion criteria for this study were stillbirth, multiple gestation, malformation and chromosomal abnormalities. Of the eligible 105 344 mother-child pairs, further exclusion was done due to no available data on vitamin D intake during pregnancy, child´s gender and birth weight and length, and implausible maternal energy intakes (<1075 kcal and >4780 kcal per day) [32]. The final study population included 66 840 mother-child pairs (58 724 mothers and 66 840 children) with available information on all covariates and at least one postnatal measurement on weight and length/height. There are more children than mothers because 22 039 of the children were siblings. The retention rate of our study population by follow-up is presented in Supplementary Table 1.

Maternal vitamin D intake

Maternal diet during pregnancy was assessed by a validated semi-quantitative Food Frequency Questionnaire (FFQ), during gestational week 22. This FFQ was used in the MoBa study from the year 2002 and onwards [32]. Pregnant women were asked to report how often they consumed 225 different food and beverage items and their supplement use since becoming pregnant. The FFQ was tailored to capture dietary habits and supplement use during the first half of pregnancy. Of all the participating women in MoBa, 93% answered the FFQ [32]. Validation studies on a subset of the MoBa population demonstrated adequate estimates of the intake and fair ability of the FFQ to rank individuals according to food, energy and nutrient intakes [33]. The Spearman's rank correlation coefficient between maternal vitamin D intake and plasma 25OHD concentration was $r=0.32$ ($p<0.01$) for all participants of the validation study ($n=119$) and $r=0.45$ ($p<0.01$) when only individuals

For peer review only - http://bmjopen.bmj.com/site/about/guidelines.xhtml

recruited during October to April were included [34]. Total vitamin D (from foods and supplements), vitamin D from foods alone and vitamin D from supplements alone were estimated from the FFQ, and used in our study.

Maternal vitamin D intake was categorized according to national recommended intakes at the time of MoBa recruitment as well as according to current recommendations [35]. Total vitamin D intake (from foods and supplements), and vitamin D intake from supplements alone, were categorized into; <5 µg/day, 5-9.9 µg/day, 10-15 µg/day and >15 µg/day. The categories for vitamin D intake from foods alone were; <2.5 µg/day, 2.5-4.9 µg/day, 5.0-7.5 µg/day and >7.5 µg/day.

Childhood anthropometry

Measurements of weight and height and the dates of the measurements of the children were obtained at eleven ages (6 weeks, 3 months, 6 months and 8 months and 1 year, 1.5 years, 2 years, 3 years, 5 years, 7 years and 8 years) by six questionnaires. During the first 18 months postpartum, the mothers were asked to report the child´s documented anthropometric measures and dates from the health card, but no specification was made for measurements onwards. There were on average seven reported measurements of weight and height respectively per child. From the available reported growth measurements, individual growth trajectories for weight and height were predicted using the Jenss-Bayley growth curve model [36, 37] with the Saemix algorithm [37] applied. Hence, the predicted measures of weight and height were obtained on the dates of the reported anthropometrics. Implausible values of the reported anthropometrics were identified and removed if they deviated ≥ 3 standard deviations (SD) from the predicted value, derived from the growth curve model. About 3% (n=50 331) of the weight and height values were excluded as implausible.

Outcomes

After exclusion of implausible reported anthropometric values, individual growth trajectories of weight and height, as well as weight growth velocity (g/month) and height growth velocity (cm/month) were predicted at eleven different age points (1 month, 3 months, 6 months, 9 months, 1 year, 1.5 years, 2 years, 3 years, 5 years, 7 years and 8 years). The trajectories and velocities were predicted at the exact age points (the exact day the child turned 1 month, 3 months, etc.), separately for girls and boys, in order to compensate for the loss to follow-up of anthropometric measures. By not including birth weight and length in the models of growth, the effect of maternal vitamin D intake on growth could be assessed independently of its

For peer review only

potential effect on birth size [27, 28], as birth size might influence the estimated growth trajectories.

Rapid weight and height growth in infancy and toddlerhood (up to 2 years) were evaluated using conditional growth modeling, an approach to obtain growth measures of weight and height independent of their prior measurements [38]. Reported anthropometrics were used for the calculation of conditional weight and height at 12 months and at 2 years. Standardized residuals were calculated by a regression of the anthropometric measure of interest, adjusting for its prior measurements. Rapid weight or height growth were defined as the residual deviating >1SD from the expected value. All other children were defined as non-rapid growers and were used as the reference.

Children's BMI values were calculated at pre-school age (3 years and 5 years) and school age (8 years) using the predicted values from the growth model. By using the extended International Obesity Task Force (IOTF) gender-specific cut offs [39], overweight and obesity were defined. The outcome childhood overweight includes obesity (further denoted as overweight). The BMI cut-offs used for categorization at the different ages are presented in Supplementary Table 2.

Statistical analysis

Differences in characteristics of the study population by categories of maternal vitamin D intake were investigated using Chi-2 test for categorical variables and ANOVA for continuous variables. The association between maternal vitamin D intake and predicted weight and height growth trajectories and velocities at the eleven time points were examined by multilevel mixed effects linear regression model, with a random intercept by child and random slope for age. The covariates were included as fixed in the mixed effect models. Logistic regression was used to investigate the association between total maternal vitamin D intake and the risk to experience rapid growth in infancy and toddlerhood, and risk of overweight in pre-school and school ages. The lowest categories of maternal vitamin D intake was used as the reference group (for total intake and for supplementary intake <5 µg/day), and for food intake <2.5 µg/day). Our primary exposure was total maternal vitamin D intake and in secondary analyses, we examined intakes from food and supplements separately.

By drawing a directed acyclic graph, hypotheses about the causal paths were set and potential confounders identified (Supplementary Figure 1). Based on the pathways in the graph, models were adjusted for maternal education, parity, maternal milk and yoghurt

For peer review only

intake, maternal fiber intake, maternal pre-pregnancy BMI and child´s gender. Maternal age and maternal energy intake were also considered but not included in the final models, as these variables did not change the estimated effect. Since some mothers participated in more than one mother-child pair, all regression models were also adjusted for random effects of sibling clusters. Potential interaction variables tested were maternal pre-pregnancy BMI (in kg/m^2) [40, 41] and birth weight [28, 42]. Interaction were considered if p<0.100 for the included interaction variable. The only significant interaction variable was pre-pregnancy BMI (p<0.001). Therefore, results are presented for all mothers, and separately for children of mothers with pre-pregnancy BMI 18,5-24,9 kg/m^2 (further denoted as mothers with normal weight), and for children of mothers with pre-pregnancy BMI ≥25 kg/m^2 (further denoted as mothers with overweight). Maternal age was divided into four categories (<20, 20–29, 30–39, >40 years). Maternal education was divided into three categories (<13, 13-16 and >16 years). Parity was categorized as primiparous or multiparous, for women without and with children from previous than the index pregnancies, respectively. Maternal pre-pregnancy body mass index (BMI) was divided into four categories (<18.5, 18.5–24.9, 25–29.9, and >30 kg/m^2), according to the World Health Organization [43]. Smoking during pregnancy was categorized as never and ever.

Three sensitivity analyses were performed: (1) using the reported measures of weight and length/height instead of the predicted anthropometrics, (2) after excluding mothers with total vitamin D intakes >90th percentile (18,8 µg/day), (3) adjustment for birth weight in the childhood overweight models.

As an approach to estimate causal effects, discordant sibling analysis was conducted, with the purpose to control for unmeasured familial confounding [44, 45]. In total, there were 22 039 siblings identified in the study subpopulation. For all women who participated with more than one pregnancy, mean maternal total vitamin D intake by family was calculated, as well as each sibling's deviation from its family mean. A cut off at 5 µg/day was used, as this corresponds to the range of the maternal vitamin D categories. Discordant siblings were defined as having a maternal vitamin D intake deviating more than, or less than the cut off from its family mean. Finally, weight and height growth trajectories, and the risk of overweight at 3, 5 and 8 years, were compared between siblings categorized as discordant (n= 2346). The sibling in the lower vitamin D category was used as a reference.

For peer review only - http://bmjopen.bmj.com/site/about/guidelines.xhtml

The main analyses were performed using Stata version 16 statistical software (Stata Corporation, College Station, Texas, USA), and RStudio version 1.2.5042 was used for the growth models.

Results

Maternal characteristics and vitamin D intake

For mothers taking a vitamin D supplement, the supplement contributed overall with 68% of the total vitamin D intake. Total maternal vitamin D intake was positively associated with maternal age, education, vitamin D supplement use in pregnancy, gestational weight gain and maternal energy intake, and negatively associated with parity, pre-pregnancy BMI and maternal smoking in pregnancy (Table 1). In 31% of the pregnancies, the mother had a BMI ≥25 kg/m^2. The median (25th−75th centiles) total vitamin D intake from foods and supplements was 7.2 µg/day (4.2–12.1 µg/day) for the included participants and 6.7 µg/day (3.9–11.7 µg/day) for the excluded group (n=13 826, p<0.001) (data not shown). The median vitamin D intake from foods alone was 3.1 µg/day (2.0-4.4 µg/day) for the included participants and 3.1 µg/day (2-4.5 µg/day) for the excluded group, with available information on vitamin D intake (n=16 872 mothers, p=0.589).

For peer review only

Table 1. Study population characteristics by categories of total maternal vitamin D intake during pregnancy (n=66 840)

	**Vitamin D intake <5µg/day**		**Vitamin D intake 5-9.9 µg/day**		**Vitamin D intake 10-15 µg/day**		**Vitamin D intake >15 µg/day**		**All**		
	N=21 748		N=22 718		N=11 143		N=11 231		N=66 840		
	**N**	**%**	**N**	**%**	**N**	**%**	**N**	**%**	**N**	**%**	**P-value[a]**
**Maternal age (years)**											<0.001
<20	194	0.9	135	0.6	83	0.8	82	0.7	494	0.7	
20-29	9566	44	9740	42.9	4684	42.0	4415	39.3	28 405	42.5	
30-39	11 622	53.4	12 449	54.9	6120	54.9	6429	57.3	36 620	54.8	
>40	366	1.7	394	2.3	256	2.3	305	2.7	1321	2.0	
**Maternal education (years)**											
<13	7480	34.4	6371	28.1	2923	26.2	2822	25.1	19 596	29.3	<0.001
13-16	9238	42.5	10 138	44.6	4954	44.5	4899	43.6	29 229	43.7	
>16	5030	23.1	6209	27.3	3266	29.3	3510	31.3	18 015	27.0	
**Parity**											<0.001
Primiparous	8733	40.2	10 546	46.4	5469	49.1	5677	50.6	30 425	45.5	
Multiparious	13 015	59.8	12 172	53.6	5674	50.9	5554	49.4	36 415	54.5	
**Pre-pregnancy BMI (kg/m^2)**											<0.001
<18.5	534	2.5	685	3	336	3	358	3.2	1913	2.9	
18.5-24.9	13 320	61.2	15 087	66.4	7599	68.2	7974	71	43 980	65.8	
25-29.9	5385	24.8	4870	21.4	2273	20.4	2152	19.2	14 680	22	
>30	2509	11.5	2076	9.1	935	8.4	747	6.7	6267	9.4	
**Maternal smoking in pregnancy**											<0.001
Never	17 759	90.7	19 278	92.7	9643	93.8	9707	93.8	56 387	92.4	
Ever	1826	9.3	1525	7.3	640	6.2	648	6.2	4628	7.6	
**Maternal vitamin D supplement use**	9134	42	20 446	90	11 031	99	11 175	99.5	51 667	77.3	<0.001
**Child´s gender**											0.198
Girls	10 711	49.3	11 122	49	5385	48.3	5412	48.2	32 630	48.8	
Boys	11 037	50.7	11 596	51	5758	51.7	5819	51.8	34 210	51.2	
	**Median**	**Range[b]**	**Median**	**Range[b]**	**Median**	**Range[b]**	**Median**	**Range[b]**	**Median**	**Range[b]**	
**Total maternal vitamin D intake (µg/day)**	3.3	1.1-4.8	7.1	5.2-9.7	12.0	10.2-14.6	19.8	15.4-33.6	7.2	1.8-23.0	<0.001
**Gestational age (weeks)**	40.1	37.3-42.1	40.1	37.3-42.1	40.3	37.3-42.1	40.3	37.3-42.1	40.1	37.3-42.1	0.224
**Gestational weight gain (kg)**	14.2	5-25	14.5	6-25	14.4	6-25	15.0	6-25	14.5	6-25	<0.001
**Birth weight (g)**	3630	2780-4480	3620	2780-4470	3620	2800-4430	3620	2780-4460	3620	2780-4464	<0.001
**Maternal energy intake (kcal)**	2092	1384-3166	2261	1491-3462	2321	1521-3558	2324	1527-3631	2222	1457-3436	<0.001

[a]Chi-2 test for categorical variables and ANOVA for continuous variables

[b]5th to 95th percentile

For peer review only

Total maternal vitamin D intake during pregnancy and childhood growth

Weight and height growth trajectories and velocities from 1 month to 8 years

Overall, total maternal vitamin D intake was negatively associated with child weight growth from 1 month to 8 years for intakes ≥10 µg/day, compared to low intakes (<5 µg/day, reference group) (Table 2 and 3). Pre-pregnancy BMI modified the association between the exposure and the outcome (p-value for interaction <0.001). More specifically, among mothers with normal pre-pregnancy BMI, we found that higher maternal vitamin D intake was negatively associated with child's weight, especially during the first 3 months, compared to low intakes (Table 2 and 3). The negative association with child's weight persisted for intakes of 10-15 µg/day and for up to 2 years, while no further reduction in weight was observed for higher intakes and after 3 months (>15 µg/day). Similar negative associations with weight from 3 months to 2 years were found when we examined maternal vitamin D intake from supplements only (Supplementary Table 3 and 4). In addition, maternal vitamin D intakes of 10-15 µg/day were associated with a lower height trajectory, compared to lower intakes, while no other intake level was associated with the child's height. No association was found between maternal vitamin D intake and child´s weight or height growth velocity from 1 month to 8 years of age, in children of mothers with normal weight (Supplementary Table 8 and 9).

Among children of mothers with overweight pre-pregnancy BMI, any maternal vitamin D intake was positively associated with weight trajectory from 12 months onwards, compared to lower intakes (Table 2 and 3). Nevertheless, for intakes between 10-15 µg/day, the positive association with child´s weight growth was already observed from 6 months and onwards. Similar positive associations with child weight after 3 years were found when we examined the intake from food and supplements separately, but for different intake levels (Supplementary Table 3 and 4). For height growth trajectory, the only significant difference from the reference group was a positive association in children prenatally exposed to vitamin D intake of >15 µg/day at 7 and 8 years of age, but the effect estimate was small. No association was found between maternal vitamin D intake and weight or height growth velocity from 1 month to 8 years of age, in children of mothers with overweight (Supplementary Table 8 and 9).

The sensitivity analyses on the association between maternal vitamin D intake and reported anthropometrics, gave similar results as the main models, in both children of mothers with

For peer review only

normal weight and children of mothers with overweight (results not shown). Also, when excluding the highest 90th percentile of total maternal vitamin D intake, the associations seen in the main models on child´s weights and heights remained.

For peer review only

Table 2. Associations between total maternal vitamin D intake and childhood weight and height trajectories from 1 month to 2 years, using predicted anthropometrics.

	**Infancy**			**Toddlerhood**		
	**1 month**	**3 months**	**6 months**	**12 months**	**18 months**	**2 years**
**Maternal vitamin D intake**	**Beta (95% CI)**	**Beta (95% CI)**	**Beta (95% CI)**	**Beta (95% CI)**	**Beta (95% CI)**	**Beta (95% CI)**
All mothers n=66 840						
**Weight (g)**						
<5 µg (ref)						
5-9.9 µg	-9.4 (-19.1, 0.2)	-9.2 (-19.9, 0.6)	-8.8 (-19.2, 1.5)	-8.1 (-20.7, 4.4)	-7.4 (-23.1, 8.3)	-6.7 (-26.0, 12.5)
10-15 µg	-7.6 (-19.5, 4.3)	-9.3 (-21.3, 2.8)	-11.7 (-24.5, 1.0)	-16.7 (-32.2, -1.2)	-21.6 (-40.9, -2.3)	-26.6 (-50.3, -2.9)
>15 µg	-14.7 (-26.7, -2.7)	-15.4 (-27.6, -3.3)	-16.6 (-29.5, -3.8)	-19.0 (-34.5, -3.5)	-21.4 (-40.7, -2.1)	-23.7 (-47.4, -0.1)
**Height (cm)**						
<5 µg (ref)						
5-9.9 µg	-0.01 (-0.07, 0.04)	-0.01 (-0.06, 0.04)	-0.01 (-0.06, 0.04)	-0.01 (-0.06, 0.04)	-0.01 (-0.05, 0.04)	-0.001 (-0.05, 0.04)
10-15 µg	-0.05 (-0.11, 0.01)	-0.05 (-0.11, 0.01)	-0.05 (-0.11, 0.01)	-0.05 (-0.11, 0.003)	-0.06 (-0.11, -0.002)	-0.06 (-0.11, -0.01)
>15 µg	-0.04 (-0.10, 0.03)	-0.04 (-0.10, 0.03)	-0.03 (-0.09, 0.03)	-0.03 (-0.09, 0.03)	-0.02 (-0.08, 0.03)	-0.02 (-0.07, 0.04)
Mothers with normal weight [a] ***n=41 970***						
**Weight (g)**						
<5 µg (ref)						
5-9.9 µg	-11.5 (-22.4, -0.6)	-10.9 (-22.0, 0.14)	-10.1 (-21.8, 1.6)	-8.4 (-22.5, 5.8)	-6.7 (-24.3, 10.9)	-5.0 (-26.6, 16.6)
10-15 µg	-14.5 (-27.7,-1.2)	-15.7 (-29.1, -2.3)	-17.5 (-31.7, -3.3)	-21.1 (-38.2, -4.0)	-24.7 (-46.0, -3.5)	-28.4 (-54.5, -2.3)
>15 µg	-15.2 (-28.3, -2.1)	-14.5 (-27.8, -1.2)	-13.5 (-27.5, 0.6)	-11.4 (-28.3, 5.5)	-9.3 (-30.4, 11.7)	-7.3 (-33.1, 18.5)
**Height (cm)**						
<5 µg (ref)						
5-9.9 µg	-0.02 (-0.08, 0.04)	-0.02 (-0.08, 0.04)	-0.02 (-0.07, 0.04)	-0.02 (-0.07, 0.04)	-0.01 (-0.06, 0.04)	-0.01 (-0.06, 0.04)
10-15 µg	-0.07 (-0.14, -0.003)	-0.07 (-0.14, -0.005)	-0.08 (-0.14, -0.01)	-0.08 (-0.15, -0.02)	-0.09 (-0.15, -0.03)	-0.09 (-0.15, -0.03)
>15 µg	-0.05 (-0.12, 0.02)	-0.05 (-0.12, 0.02)	-0.05 (-0.11, 0.02)	-0.04 (-0.11, 0.02)	-0.04 (-0.09, 0.02)	-0.03 (-0.09, 0.03)
Mothers with overweight [b] ***n=20 080***						
**Weight (g)**						
<5 µg (ref)						
5-9.9 µg	-5.5 (-19.8, 8.8)	-1.4 (-15.9, 13.1)	4.7 (-10.6, 20.0)	16.9 (-1.6, 35.4)	29.2 (6.1, 52.2)	41.4 (13.1, 69.7)
10-15 µg	10.3 (-8.0, 28.6)	14.4 (-4.1, 33.0)	20.6 (1.0, 40.2)	33.1 (9.3, 56.8)	45.5 (15.9, 75.0)	57.9 (21.7, 94.1)
>15 µg	-15.6 (-34.8, 3.7)	-9.9 (-29.5, 9.6)	-1.5 (-22.1, 19.1)	15.4 (-9.5, 40.3)	32.3 (1.2, 63.3)	49.2 (11.1, 87.3)

For peer review only

**Height (cm)**						
<5 µg (ref)						
5-9.9 µg	-0.02 (-0.09, 0.06)	-0.02 (-0.09, 0.06)	-0.01 (-0.08, 0.06)	-0.005 (-0.07, 0.06)	0.002 (-0.06, 0.07)	0.009 (-0.06, 0.07)
10-15 µg	-0.02 (-0.11, 0.08)	-0.02 (-0.11, 0.08)	-0.01 (-0.10, 0.08)	-0.003 (-0.09, 0.09)	0.005 (-0.08, 0.09)	0.01 (-0.07, 0.10)
>15 µg	-0.04 (-0.14, 0.07)	-0.03 (-0.13, 0.07)	-0.03 (-0.12, 0.07)	-0.01 (-0.11, 0.08)	-0.001 (-0.09, 0.09)	0.01 (-0.08, 0.10)

Beta=Beta coefficient, CI=Confidence interval

Effect estimates derive from multilevel mixed effects linear regression model, adjusted for maternal education, parity, maternal milk and yoghurt intake, maternal fiber intake, child´s gender and birth weight. The model with all mothers was also adjusted for maternal pre-pregnancy BMI.

[a] Mothers with pre-pregnancy BMI 18.5-24.9 kg/m^2

[b] Mothers with pre-pregnancy BMI ≥25 kg/m^2

For peer review only

Table 3. Associations between total maternal vitamin D intake and childhood weight and height trajectories from 3 years to 8 years, using predicted anthropometrics.

	Preschool age		School age
	3 years	5 years	8 years
**Maternal vitamin D intake**	**Beta (95% CI)**	**Beta (95% CI)**	**Beta (95% CI)**
All mothers n=66 840			
**Weight (g)**			
<5 µg (ref)			
5-9.9 µg	-5.3 (-32.4, 21.8)	-2.5 (-46.1, 41.2)	1.8 (-67.3, 70.9)
10-15 µg	-36.5 (-69.7, -3.2)	-56.3 (-110, -2.7)	-86.0 (-171, -1.1)
>15 µg	-28.5 (-61.7, 4.8)	-37.9 (-91.4, 15.5)	-52.1 (-137, 32.5)
**Height (cm)**			
<5 µg (ref)			
5-9.9 µg	0.01 (-0.04, 0.05)	0.02 (-0.03, 0.07)	0.04 (-0.03, 0.11)
10-15 µg	-0.06 (-0.11, -0.01)	-0.07 (-0.13, -0.01)	-0.09 (-0.17, 0.003)
>15 µg	-0.01 (-0.06, 0.05)	0.02 (-0.04, 0.08)	0.05 (-0.04, 0.14)

Mothers with normal weight [a] ***n=41 970***			
**Weight (g)**			
<5 µg (ref)			
5-9.9 µg	-1.6 (-31.9, 28.7)	5.2 (-43.6, 54.0)	15.3 (-62.0, 92.6)
10-15 µg	-35.6 (-72.3, 1.0)	-50.1 (-109, 8.9)	-71.8 (-165, 21.6)
>15 µg	-3.1 (-39.3, 33.0)	5.1 (-53.0, 63.3)	17.6 (-74.6, 110)
**Height (cm)**			
<5 µg (ref)			
5-9.9 µg	-0.004 (-0.05, 0.05)	0.008 (-0.05, 0.06)	0.03 (-0.06, 0.11)
10-15 µg	-0.1 (-0.16, -0.04)	-0.1 (-0.19, -0.05)	-0.1 (-0.25, -0.05)
>15 µg	-0.02 (-0.08, 0.04)	-0.003 (-0.07, 0.07)	0.03 (-0.07, 0.12)
Mothers with overweight [b] ***n=20 080***			
**Weight (g)**			
<5 µg (ref)			
5-9.9 µg	65.8 (26.1, 106)	115 (50.8, 179)	188 (86.8, 290)
10-15 µg	82.7 (31.9, 134)	132 (50.5, 214)	207 (77.2, 337)
>15 µg	82.9 (29.5, 136)	151 (64.4, 237)	252 (116, 388)
**Height (cm)**			
<5 µg (ref)			
5-9.9 µg	0.02 (-0.04, 0.09)	0.05 (-0.02, 0.13)	0.09, (-0.01, 0.20)
10-15 µg	0.03 (-0.05, 0.11)	0.06 (-0.03, 0.16)	0.1 (-0.02, 0.25)
>15 µg	0.04 (-0.05, 0.12)	0.09 (-0.01, 0.19)	0.2 (0.02, 0.31)

Beta=Beta coefficient, CI=Confidence interval

Effect estimates derive from multilevel mixed effects linear regression model, adjusted for maternal education, parity, maternal milk and yoghurt intake, maternal fiber intake, child´s gender and birth weight. The model with all mothers was also adjusted for maternal pre-pregnancy BMI.

[a] Mothers with pre-pregnancy BMI 18.5-24.9 kg/m^2

[b] Mothers with pre-pregnancy BMI ≥25 kg/m^2

For peer review only

Risk of rapid weight and height growth in infancy and toddlerhood

Overall, maternal vitamin D intakes of 5-9.9 and >15 µg/day was associated with lower odds of having a rapid weight gain at 12 months of age, both crude and adjusted, compared to low intakes (<5 µg/day) (Table 4).

When exploring the association by maternal pre-pregnancy BMI, children of mothers with normal pre-pregnancy weight, with total maternal vitamin D intakes of 5-9.9, 10-15 and >15 µg/day during pregnancy, had lower odds of having a rapid weight gain during the first year of life, both crude and adjusted compared to the reference group (Table 4). Similar lower OR were found for the risk of rapid height gain from birth to 12 months, but the confidence interval included 1. The associations with rapid weight and height gain from birth to 2 years were weaker.

For children of mothers with pre-pregnancy overweight, we found higher odds ratio for rapid weight and height gain at 12 months and 2 years, both crude and adjusted, but these associations were weak and the confidence intervals included 1 (Table 4).

In the sensitivity analyses, when excluding the 90th percentile of maternal vitamin D intakes, similar results were obtained as in the main models, for both children of mothers with normal weight, and for children of mothers with overweight.

For peer review only

Table 4. Associations (OR and 95% CI) between total maternal vitamin D intake and rapid growth from birth to 12 months and from birth to 2 years, using reported anthropometrics.

	**Weight growth from birth**		**Height growth from birth**	
**Maternal vitamin D intake**	**12 months**	**2 years**	**12 months**	**2 years**
All mothers	n=39 296, 59% [a]	n=18 360, 27%	n=31 640, 47%	n=14 946, 22%
	**OR (95% CI)**	**OR (95% CI)**	**OR (95% CI)**	**OR (95% CI)**
<5 µg (ref)				
5-9.9 µg	0.91 (0.85, 0.97)	1.03 (0.93, 1.15)	1.05 (0.97, 1.13)	1.01 (0.90, 1.14)
10-15 µg	0.93 (0.86, 1.02)	1.02 (0.90, 1.16)	1.02 (0.93, 1.12)	0.95 (0.83, 1.09)
>15 µg	0.90 (0.83, 0.98)	0.97 (0.85, 1.10)	1.00 (0.91, 1.10)	1.04 (0.90, 1.19)
	**12 months**	**2 years**	**12 months**	**2 years**
Mothers with normal weight [b]	n=25 982, 62%	n=12 043, 29%	n=20 658, 49%	n=9700, 23%
	**OR (95% CI)**	**OR (95% CI)**	**OR (95% CI)**	**OR (95% CI)**
<5 µg (ref)				
5-9.9 µg	0.88 (0.81, 0.96)	1.04 (0.91, 1.18)	1.01 (0.91, 1.11)	1.05 (0.91, 1.21)
10-15 µg	0.85 (0.76, 0.95)	1.03 (0.89, 1.21)	0.94 (0.83, 1.06)	0.98 (0.82, 1.17)
>15 µg	0.86 (0.77, 0.95)	0.97 (0.84, 1.13)	0.94 (0.84, 1.06)	1.01 (0.85, 1.20)
	**12 months**	**2 years**	**12 months**	**2 years**

Mothers with overweight [c]	n=12 240, 61%	N=10 118, 50%	N=5838, 29%	N=4851, 24%
	**OR (95% CI)**	**OR (95% CI)**	**OR (95% CI)**	**OR (95% CI)**
<5 µg (ref)				
5-9.9 µg	0.96 (0.86, 1.08)	1.08 (0.90, 1.29)	1.12 (0.99, 1.28)	1.00 (0.82, 1.21)
10-15 µg	1.10 (0.95, 1.27)	1.01 (0.81, 1.27)	1.14 (0.97, 1.34)	0.87 (0.68, 1.12)
>15 µg	1.01 (0.87, 1.18)	0.91 (0.72, 1.16)	1.08 (0.91, 1.28)	1.08 (0.84, 1.38)

OR=Odds Ratio, CI=Confidence interval

Effect estimates derived by logistic regression, adjusted for maternal education, parity, maternal milk and yoghurt intake, maternal fiber intake and child´s gender. The model with all mothers was also adjusted for maternal pre-pregnancy BMI.

[a] Proportions of the eligible pregnancies with the reported anthropometrics available from birth to the given age.

[b] Mothers with pre-pregnancy BMI 18.5-24.9 kg/m^2

[c] Mothers with pre-pregnancy BMI ≥25 kg/m^2

For peer review only

Risk of childhood overweight in pre-school and school age

Overall, the prevalence of childhood overweight was 8.9% at 3 years, 18.2% at 5 years and 6.3% at 8 years of age. Maternal vitamin D intakes of 5-9.9 and >15 µg/day were associated with a higher risk of overweight at 8 years of age, compared to the reference group (Table 5). However, in children of mothers with normal weight, with total vitamin D intakes of 10-15 and >15 µg/day during pregnancy, 0.86 (95% CI 0.77, 0.97) and 0.88 (95% CI 0.79, 0.99) lower odds for overweight at 3 years of age were found, both crude and adjusted, compared to the reference group (Table 5). At 5 years, children of mothers with vitamin D intakes of 10-15 µg/day during pregnancy, had 0.91 (95% CI 0.84, 0.99) lower adjusted odds of being overweight, compared to the reference group. At 8 years there were no association found for any intake group. For children of mothers with overweight, we found an increased risk of overweight at 5 years (OR=1.09, 95% CI 1.01, 1.18) and at 8 years (OR=1.12, 95% CI 1.02, 1.23) for children of mothers with vitamin D intakes of 5-9.9 µg/day, compared to the reference group (Table 5). However, no association between maternal vitamin D intake and risk of overweight was found for children of mothers with intakes of 10-15 or >15 µg/day at 3 years, 5 years or 8 years of age.

In the sensitivity analyses on childhood overweight using the reported weights and heights, the results for the children of mothers with normal weight, with vitamin D intake >15 µg/day persisted (OR=0.87, 95% CI 0.77, 0.98) (Supplementary Table 10). Also, for children of mothers with overweight, with total vitamin D intake of 5-9.9 µg/day, a significant odds ratio (OR=1.22, 95% CI 1.06, 1.40) was obtained. After exclusion of the highest 90th percentile of maternal vitamin D intakes, similar results as in the main models were obtained, both in children of mothers with normal weight and with overweight. Adjusting for birth weight did

not change the direction of the associations, neither in children of mothers with normal weight, nor in children of mothers with overweight.

Table 5. Associations (OR and 95% CI) between total maternal vitamin D intake and risk of childhood overweight at 3, 5 and 8 years, using predicted anthropometrics.

Maternal vitamin D intake	3 years<br>OR (95% CI)	5 years<br>OR (95% CI)	8 years<br>OR (95% CI)
All mothers n=66 840			
<5 µg (ref)			
5-9.9 µg	0.99 (0.93, 1.06)	1.03 (0.98, 1.08)	1.10 (1.03, 1.17)
10-15 µg	0.93 (0.86, 1.01)	0.97 (0.91, 1.04)	1.02 (0.94, 1.11)
>15 µg	0.97 (0.89, 1.06)	1.00 (0.94, 1.06)	1.09 (1.01, 1.19)
p-value for trend [a]	0.222	0.553	0.098
Mothers with normal weight [b] n=41 970			
<5 µg (ref)			
5-9.9 µg	0.92 (0.84, 1.01)	0.97 (0.91, 1.04)	1.07 (0.98, 1.17)
10-15 µg	0.86 (0.77, 0.97)	0.91 (0.84, 0.99)	0.98 (0.87, 1.09)
>15 µg	0.88 (0.79, 0.99)	0.94 (0.87, 1.02)	1.05 (0.94, 1.17)
p-value for trend [a]	0.015	0.039	0.920
Mothers with overweight [c] n=20 080			
<5 µg (ref)			
5-9.9 µg	1.07 (0.97, 1.18)	1.09 (1.01, 1.18)	1.12 (1.02, 1.23)
10-15 µg	1.01 (0.89, 1.14)	1.05 (0.95, 1.16)	1.06 (0.94, 1.20)
>15 µg	1.06 (0.93, 1.20)	1.03 (0.93, 1.14)	1.11 (0.98, 1.26)
p-value for trend [a]	0.513	0.443	0.156

OR=Odds Ratio, CI=Confidence interval

Effect estimates derived by logistic regression, adjusted for maternal education, parity, maternal milk and yoghurt intake, maternal fiber intake and child´s gender. The model with all mothers was also adjusted for maternal pre-pregnancy BMI.

[a] P-value for linear trend, obtained by regression with maternal vitamin D intake in categories as continuous variable.

[b] Mothers with pre-pregnancy BMI 18.5-24.9 kg/m^2

[c] Mothers with pre-pregnancy BMI ≥25 kg/m^2

Siblings with discordant total maternal vitamin D intake

When comparing weight and height growth trajectories of discordant siblings, the analyzes showed similar estimates as the main models, but non-significant differences (Supplementary Table 5 and 6). The growth trajectories did not differ between siblings with discordant maternal vitamin D intake. When comparing the risk of childhood overweight at 3 years, 5 years and 8 years in discordant siblings, the same effect estimates as for the main models were obtained, however not significant (Supplementary Table 7).

Discussion

In this longitudinal study of the associations between maternal vitamin D intake and child growth, we identified pre-pregnancy BMI as a major modifying factor. Among mothers with normal pre-pregnancy BMI, a maternal vitamin D intake ≥10 µg/day was associated with

For peer review only

lower weight and height growth trajectories starting in infancy and persisting in toddlerhood. Higher maternal vitamin D intake was also associated with lower odds of a rapid weight gain during the first year of life and of child overweight in pre-school years. The results indicated associations in opposing directions in children of mothers with pre-pregnancy overweight. The discordant sibling analysis revealed similar but weaker associations, possibly due to small sample size, and supports the hypothesis. To our knowledge, no previous study has investigated the association between maternal vitamin D intake and childhood growth, risk of rapid growth or overweight. Our findings suggest that in children of normal weight mothers, vitamin D intake during pregnancy has a protective effect against rapid growth and overweight, perhaps due to a more favorable weight growth trajectory in infancy.

The main strengths of this study are the longitudinal design and the large, nationwide [29] study sample. The FFQ, used to capture the dietary vitamin D intake, was customized for assessing diet in the first half of pregnancy. Validation studies on the FFQ concluded the questionnaire to provide adequate estimates of vitamin D intakes from foods and supplements [33, 34]. Another strength was the use of a growth curve model, which is an approach to compensate for the loss to follow-up, by predicting weights and heights for all children. The similar estimates found in the sensitivity analyses of the reported weights and heights of the children bring some validity in the predicted anthropometrics of growth trajectories and childhood overweight. In addition, the drawing of a directed acyclic graph reduced the risk of confounding bias, since the models were adjusted for relevant covariates. Our study also has several weaknesses. Firstly, maternal anthropometrics, child anthropometrics and food intake measures are self-reported and prone to misreport [33, 46, 47]. Although the women were not aware of the outcome of interest, maternal weight status and other characteristic are likely to influence the accuracy of self-reported information. It is known that overweight and obese women misreport their bodyweight and food intake to a larger degree than normal weight women, pertaining also to pregnant women [46]. Although self-reported weight and height encompass inaccuracy, self-report is a cost-effective alternative in large cohorts and the inaccuracy is not likely to largely bias the results [48]. It is also possible that the association we found could be explained by another food, nutrient or behavior related to the maternal vitamin D intake that was not taken into account, or residual confounding.

The protective effect of maternal vitamin D in pregnancy on the risk of overweight in children from mothers with pre-pregnancy normal weight is consistent with previous findings from a Dutch [19] and a Spanish study [22]. However, previous studies investigating the

For peer review only

association between maternal vitamin D status and childhood growth or overweight, have explored the association by adjusting for pre-pregnancy BMI. We are the first study to report an interaction of pre-pregnancy maternal BMI when investigating the relation between maternal vitamin D intake with child growth and overweight. To simply adjust for pre-pregnancy BMI could potentially fail to capture the variation in maternal body size and thereby the magnitude of the distribution and dilution of vitamin D in the body [5]. This may explain the somewhat opposing results found in previous studies on the association on maternal vitamin D status and child´s growth, anthropometrics and risk of overweight [19, 20, 22-26].

Our results emphasize the importance of adequate vitamin D intake for the pregnant woman on growth in childhood. Also, our results illuminate the significant contribution of supplements to reach the recommended vitamin D intake in a population with relatively low intake from foods. The findings from our study suggest that maternal vitamin D intake is a potential modifiable factor for prevention of rapid postnatal growth in children of normal weight mothers. However, given the inverse association between BMI and vitamin D status [5], the increase in risk of childhood overweight in children of mothers with overweight, is unlikely driven by a higher maternal vitamin D intake. Since, there is a lack of previous studies investigating the association between maternal vitamin D intake and childhood growth and overweight, further studies will need to replicate our results before the relevance can be fully decided.

Conclusion

Our findings supports that vitamin D intake during pregnancy affects offspring postnatal growth, and that a higher vitamin D intake during pregnancy has a protective effect against the risk of childhood overweight but only in children of mothers with normal pre-pregnancy weight. In pregnancy, achieving the recommended vitamin D intake may reduce the risks of overweight in children of mothers with normal weight.

Acknowledgements

The Norwegian Mother, Father and Child Cohort Study is supported by the Norwegian Ministry of Health and Care Services and the Ministry of Education and Research. The authors thank the MoBa cohort funders and all the participating families in Norway for their valuable contribution.

For peer review only

Author contributions

HA initiated the study, all authors contributed with planning the study, ALB is responsible for data protection and access, AA conducted the statistical analyses and wrote the first version of the manuscript, EP assisted with the statistical analyses. All authors (HA, AA, EP, ALB, LL, AW and HMM) were involved in interpretation of the results and writing of the final manuscript.

Competing interests

None declared.

Patient consent

Obtained.

Funding statement

The current study was funded by the Swedish Research Council for Health, Working Life and Welfare (grant number 2018-00441) and the Sahlgrenska Academy (U2018/162). EP was funded by the Research Council of Norway, under the MILJØFORSK program (project number 268465).

Data sharing statement

No additional data are available. All data from the MoBa study are available to all qualified researchers/research groups in Norway and to international researchers who are collaborating with a Norwegian researcher.

For peer review only

References

1. Theodoratou, E., et al., *Vitamin D and multiple health outcomes: umbrella review of systematic reviews and meta-analyses of observational studies and randomised trials.* Bmj, 2014. **348**: p. g2035.
2. Hossein-nezhad, A. and M.F. Holick, *Optimize dietary intake of vitamin D: an epigenetic perspective.* Curr Opin Clin Nutr Metab Care, 2012. **15**(6): p. 567-79.
3. O'Neill, C.M., et al., *Seasonal Changes in Vitamin D-Effective UVB Availability in Europe and Associations with Population Serum 25-Hydroxyvitamin D.* Nutrients, 2016. **8**(9).
4. Hossein-nezhad, A. and M.F. Holick, *Vitamin D for health: a global perspective.* Mayo Clin Proc, 2013. **88**(7): p. 720-55.
5. Walsh, J.S., S. Bowles, and A.L. Evans, *Vitamin D in obesity.* Curr Opin Endocrinol Diabetes Obes, 2017. **24**(6): p. 389-394.
6. Saraf, R., et al., *Global summary of maternal and newborn vitamin D status - a systematic review.* Matern Child Nutr, 2016. **12**(4): p. 647-68.
7. Eggemoen, Å.R., et al., *Vitamin D levels during pregnancy and associations with birth weight and body composition of the newborn: a longitudinal multiethnic population-based study.* The British journal of nutrition, 2017. **117**(7): p. 985-993.
8. Barebring, L., et al., *Vitamin D Status during Pregnancy in a Multi-Ethnic Population-Representative Swedish Cohort.* Nutrients, 2016. **8**(10).
9. Itkonen, S.T., et al., *Vitamin D status and current policies to achieve adequate vitamin D intake in the Nordic countries.* Scand J Public Health, 2020: p. 1403494819896878.
10. Gluckman, P.D., M.A. Hanson, and T. Buklijas, *A conceptual framework for the developmental origins of health and disease.* J Dev Orig Health Dis, 2010. **1**(1): p. 6-18.
11. Kwon, E.J. and Y.J. Kim, *What is fetal programming?: a lifetime health is under the control of in utero health.* Obstet Gynecol Sci, 2017. **60**(6): p. 506-519.
12. Robinson, S.M., et al., *Modifiable early-life risk factors for childhood adiposity and overweight: an analysis of their combined impact and potential for prevention.* Am J Clin Nutr, 2015. **101**(2): p. 368-75.
13. Ong, K.K. and R.J. Loos, *Rapid infancy weight gain and subsequent obesity: systematic reviews and hopeful suggestions.* Acta Paediatr, 2006. **95**(8): p. 904-8.
14. Woo Baidal, J.A., et al., *Risk Factors for Childhood Obesity in the First 1,000 Days: A Systematic Review.* Am J Prev Med, 2016. **50**(6): p. 761-779.
15. Karaolis-Danckert, N., et al., *Rapid growth among term children whose birth weight was appropriate for gestational age has a longer lasting effect on body fat percentage than on body mass index.* Am J Clin Nutr, 2006. **84**(6): p. 1449-55.
16. WHO, *Commission on Ending Childhood Obesity. Report of the Commission on Ending Childhood Obesity*. 2016.
17. *UNICEF, WHO, World Bank. Global and regional trends by UN Regions, 1990-2030. Overweight: 1990-2018* 2015 [cited 2020.
18. Aghajafari, F., et al., *Association between maternal serum 25-hydroxyvitamin D level and pregnancy and neonatal outcomes: systematic review and meta-analysis of observational studies.* Bmj, 2013. **346**: p. f1169.
19. Leffelaar, E.R., T.G.M. Vrijkotte, and M. van Eijsden, *Maternal early pregnancy vitamin D status in relation to fetal and neonatal growth: results of the multi-ethnic Amsterdam Born Children and their Development cohort.* The British journal of nutrition, 2010. **104**(1): p. 108-117.
20. Crozier, S.R., et al., *Maternal vitamin D status in pregnancy is associated with adiposity in the offspring: findings from the Southampton Women's Survey.* The American journal of clinical nutrition, 2012. **96**(1): p. 57-63.

For peer review only

21. Krishnaveni, G.V., et al., *Maternal vitamin D status during pregnancy and body composition and cardiovascular risk markers in Indian children: the Mysore Parthenon Study.* The American journal of clinical nutrition, 2011. **93**(3): p. 628-635.
22. Morales, E., et al., *Deficit of vitamin D in pregnancy and growth and overweight in the offspring.* International journal of obesity (2005), 2015. **39**(1): p. 61-68.
23. Daraki, V., et al., *Low maternal vitamin D status in pregnancy increases the risk of childhood obesity.* Pediatric obesity, 2018. **13**(8): p. 467-475.
24. Eckhardt, C.L., et al., *Maternal vitamin D status and infant anthropometry in a US multi-centre cohort study.* Annals of human biology, 2015. **42**(3): p. 215-222.
25. Ong, Y.L., et al., *The association of maternal vitamin D status with infant birth outcomes, postnatal growth and adiposity in the first 2 years of life in a multi-ethnic Asian population: the Growing Up in Singapore Towards healthy Outcomes (GUSTO) cohort study.* The British journal of nutrition, 2016. **116**(4): p. 621-631.
26. Gale, C.R., et al., *Maternal vitamin D status during pregnancy and child outcomes.* European journal of clinical nutrition, 2008. **62**(1): p. 68-77.
27. Horan, M.K., et al., *The association between maternal dietary micronutrient intake and neonatal anthropometry - secondary analysis from the ROLO study.* Nutrition journal, 2015. **14**: p. 105-105.
28. Scholl, T.O. and X. Chen, *Vitamin D intake during pregnancy: association with maternal characteristics and infant birth weight.* Early human development, 2009. **85**(4): p. 231-234.
29. Magnus, P., et al., *Cohort Profile Update: The Norwegian Mother and Child Cohort Study (MoBa).* Int J Epidemiol, 2016. **45**(2): p. 382-8.
30. Magnus, P., et al., *Cohort profile: the Norwegian Mother and Child Cohort Study (MoBa).* Int J Epidemiol, 2006. **35**(5): p. 1146-50.
31. Irgens, L.M., *The Medical Birth Registry of Norway. Epidemiological research and surveillance throughout 30 years.* Acta Obstet Gynecol Scand, 2000. **79**(6): p. 435-9.
32. Meltzer, H.M., et al., *Methodological challenges when monitoring the diet of pregnant women in a large study: experiences from the Norwegian Mother and Child Cohort Study (MoBa).* Matern Child Nutr, 2008. **4**(1): p. 14-27.
33. Brantsaeter, A.L., et al., *Validity of a new food frequency questionnaire for pregnant women in the Norwegian Mother and Child Cohort Study (MoBa).* Matern Child Nutr, 2008. **4**(1): p. 28-43.
34. Brantsaeter, A.L., et al., *Self-reported dietary supplement use is confirmed by biological markers in the Norwegian Mother and Child Cohort Study (MoBa).* Ann Nutr Metab, 2007. **51**(2): p. 146-54.
35. Nordic Council of Ministers, N.C.o.M.S., *Nordic Nutrition Recommendations 2012: Integrating nutrition and physical activity* 2014.
36. Jenss RM, B.N., *A mathematical method for studying the growth of a child. .* Human Biology, 1937. **9**: p. 556-563.
37. Chirwa, E.D., et al., *Multi-level modelling of longitudinal child growth data from the Birth-to-Twenty Cohort: a comparison of growth models.* Annals of human biology, 2014. **41**(2): p. 168-179.
38. Keijzer-Veen, M.G., et al., *A regression model with unexplained residuals was preferred in the analysis of the fetal origins of adult diseases hypothesis.* J Clin Epidemiol, 2005. **58**(12): p. 1320-4.
39. Cole, T.J. and T. Lobstein, *Extended international (IOTF) body mass index cut-offs for thinness, overweight and obesity.* Pediatr Obes, 2012. **7**(4): p. 284-94.
40. Patro Golab, B., et al., *Influence of maternal obesity on the association between common pregnancy complications and risk of childhood obesity: an individual participant data meta-analysis.* Lancet Child Adolesc Health, 2018. **2**(11): p. 812-821.

For peer review only

41. Drincic, A.T., et al., *Volumetric dilution, rather than sequestration best explains the low vitamin D status of obesity.* Obesity (Silver Spring), 2012. **20**(7): p. 1444-8.
42. Rito, A.I., et al., *Association between Characteristics at Birth, Breastfeeding and Obesity in 22 Countries: The WHO European Childhood Obesity Surveillance Initiative - COSI 2015/2017.* Obes Facts, 2019. **12**(2): p. 226-243.
43. WHO, *Obesity: Preventing and managing the global epidemic. Report of a WHO consultation on obesity. p. 1–253*. 2000.
44. Petersen, A.H. and T. Lange, *What Is the Causal Interpretation of Sibling Comparison Designs?* Epidemiology, 2020. **31**(1): p. 75-81.
45. Susser, E., M.G. Eide, and M. Begg, *Invited commentary: The use of sibship studies to detect familial confounding.* Am J Epidemiol, 2010. **172**(5): p. 537-9.
46. Engstrom, J.L., et al., *Accuracy of self-reported height and weight in women: an integrative review of the literature.* J Midwifery Womens Health, 2003. **48**(5): p. 338-45.
47. Olafsdottir, A.S., et al., *Comparison of women's diet assessed by FFQs and 24-hour recalls with and without underreporters: associations with biomarkers.* Ann Nutr Metab, 2006. **50**(5): p. 450-60.
48. Headen, I., et al., *The accuracy of self-reported pregnancy-related weight: a systematic review.* Obes Rev, 2017. **18**(3): p. 350-369.

For peer review only

Supplementary table 1. Study population retention and anthropometric measures of the children at the 11 ages.

Intended age	Reported age	Weight (kg)		Response rate	Height (cm)		Response rate
	Mean (SD)	N	Mean (SD)	%	N	Mean (SD)	%
1.5 months	1.4 (0.2) months	56 625	5.0 (0.7)	84	45 745	57 (2.3)	67
3 months	3.1 (0.3) months	62 237	6.4 (0.8)	92	61 266	62 (2.5)	91
6 months	5.8 (0.5) months	60 515	7.9 (1.0)	89	60 224	68 (2.5)	89
8 months	8.0 (0.8) months	49 528	8.8 (1.0)	73	49 354	71 (2.7)	73
12 months	12.2 (0.6) months	49 609	9.9 (1.1)	73	49 686	76 (2.7)	73
15-18 months	16.0 (1.3) months	50 393	10.9 (1.2)	75	50 168	81 (3.0)	74
2 years	2.1 (0.2) years	25 195	13.0 (1.5)	37	25 346	89 (3.7)	37
3 years	3.0 (0.1) years	36 987	15.1 (1.8)	55	36 365	97 (3.8)	54
5 years	5.2 (0.3) years	30 288	20.0 (2.8)	45	31 671	113 (5.2)	47
7 years	7.1 (0.1) years	36 480	25.1 (4.0)	54	38 130	126 (5.4)	56
8 years	8.1 (0.1) years	23 123	28.2 (4.5)	34	24 611	132 (5.7)	36

For peer review only

Supplementary table 2. Cut-offs of overweight in children

Age (years)	Overweight cut-offs[a, b]		Prevalence (%)[c]	
	Boys	Girls	Boys	Girls
**3**	17.85	17.64	9.9	9.5
**5**	17.39	17.23	18.8	20.5
**8**	18.41	18.28	5.5	8.4

[a] Cole TJ, Lobstein T. Extended international (IOTF) body mass index cut-offs for thinness, overweight and obesity. Pediatr Obes. 2012;7(4):284-294. doi:10.1111/j.2047-6310.2012.00064.x

[b] All children with a body mass index equal to, or above, the cut-off are denoted as overweight

[c] Based on child´s body mass index calculated from the predicted anthropometric data.

For peer review only

Supplementary table 3. Associations between maternal vitamin D intake from foods alone, and from supplements alone, and predicted weight growth trajectories from 1 month to 2 years of age.

	**Infancy**			**Toddlerhood**		
	**1 month**	**3 months**	**6 months**	**12 months**	**18 months**	**2 years**
	**Beta (95% CI)**	**Beta (95% CI)**	**Beta (95% CI)**	**Beta (95% CI)**	**Beta (95% CI)**	**Beta (95% CI)**
**Maternal vitamin D intake from foods alone**						
All mothers n=66 840						
<2.5 µg						
2.5-4.9 µg	-5.8 (-14.6, 2.99)	-6.2 (-15.1, 2.7)	-6.7 (-16.1, 2.6)	-7.9 (-19.2, 3.5)	-9.0 (-23.1, 5.1)	-10.2 (-27.4, 7.1)
5-7.5 µg	-9.7 (-22.8, 3.5)	-7.1 (-20.4, 6.2)	-3.3 (-17.4, 10.7)	4.3 (-12.5, 21.1)	11.9 (-8.9, 32.7)	19.5 (-5.9, 45.0)
>7.5 µg	-12.0 (-34.8, 10.8)	-11.6 (-34.7, 11.4)	-11.0 (-35.3, 13.3)	-9.8 (-39.0, 19.3)	-8.7 (-44.7, 27.4)	-7.5 (-51.6, 36.6)
Mothers with normal weight [a] n=41 970						
<2.5 µg						
2.5-4.9 µg	-6.4 (-16.2, 3.4)	-7.0 (-16.9, 3.0)	-7.8 (-18.3, 2.7)	-9.3 (-22.0, 3.4)	-10.9 (-26.7, 4.9)	-12.5 (-31.9, 6.9)
5-7.5 µg	-7.3 (-21.7, 7.1)	-5.4 (-19.9, 9.2)	-2.5 (-17.8, 12.9)	3.3 (-15.1, 21.8)	9.1 (-13.8, 32.0)	14.9 (-13.1, 42.9)
>7.5 µg	-14.9 (-40.1, 10.3)	-14.8 (-40.3, 10.7)	-14.6 (-41.5, 12.3)	-14.1 (-46.5, 18.2)	-13.7 (-53.8, 26.4)	-13.2 (-62.3, 35.8)
Mothers with overweigh [b] n=20 080						
<2.5 µg						
2.5-4.9 µg	-4.3 (-17.5, 9.0)	-4.3 (-17.7, 9.2)	-4.3 (-18.5, 9.9)	-4.4 (-21.6, 12.8)	-4.5 (-25.9, 16.9)	-4.6 (-30.9, 21.7)
5-7.5 µg	-15.3 (-35.2, 4.6)	-11.3 (-31.4, 8.8)	-5.3 (-26.6, 15.9)	6.6 (-19.0, 32.3)	18.6 (-13.2, 50.4)	30.6 (-8.4, 69.6)
>7.5 µg	-5.1 (-39.1, 28.9)	-4.1 (-38.5, 30.3)	-2.6 (-38.9, 33.7)	0.3 (-43.5, 44.1)	3.3 (-51.1, 57.7)	6.2 (-60.4, 72.8)
**Maternal vitamin D intake from supplements alone**						
All mothers n=66 840						
<5 µg						
5-9.9 µg	-11.8 (-21.6, -2.1)	-11.4 (-21.3, -1.5)	-10.8 (-21.2, -0.3)	-9.5 (-22.1, 3.2)	-8.2 (-23.9, 7.6)	-6.9 (-26.2, 12.4)
10-15 µg	-4.0 (-17.4, 9.3)	-4.1 (-17.6, 9.4)	-4.1 (-18.4, 10.2)	-4.1 (-21.4, 13.2)	-4.2 (-25.7, 17.3)	-4.2 (-30.7, 22.2)
>15 µg	-8.5 (-21.5, 4.5)	-7.0 (-20.1, 6.2)	-4.6 (-18.5, 9.3)	0.1 (-16.8, 17.9)	4.8 (-16.2, 25.8)	9.5 (-16.2, 35.3)
Mothers with normal weight [a] n=41 970						
<5 µg						

5-9.9 µg	-10.6 (-21.4, 0.2)	-11.7 (-22.6, -0.7)	-13.3 (-24.9, -1.8)	-16.6 (-30.6, -2.6)	-19.9 (-37.4, -2.5)	-23.2 (-44.6, -1.8)
10-15 µg	-5.8 (-20.4, 8.7)	-7.1 (-21.9, 7.6)	-9.1 (-24.7, 6.5)	-13.0 (-31.9, 5.9)	-16.9 (-40.5, 6.6)	-20.9 (-49.7, 8.0)
>15 µg	-8.6 (-22.5, 5.4)	-8.2 (-22.4, 5.9)	-7.7 (-22.6, 7.3)	-6.6 (-24.7, 11.5)	-5.6 (-28.1, 17.0)	-4.5 (-32.2, 23.2)
Mothers with overweight* [b]**<br>n=20 080***						
<5 µg						
5-9.9 µg	-14.9 (-29.8, 0.1)	-10.8 (-25.9, 4.3)	-4.7 (-20.7, 11.3)	7.5 (-11.8, 26.9)	20.0 (-4.4, 43.9)	32.0 (2.3, 61.6)
10-15 µg	0.2 (-21.6, 22.0)	3.2 (-18.8, 25.3)	7.8 (-15.5, 31.2)	17.0 (-11.3, 45.3)	26.1 (-9.2, 61.4)	35.2 (-8.1, 78.6)
>15 µg	-8.4 (-30.2, 13.4)	-4.0 (-26.0, 18.1)	2.7 (-20.6, 26.0)	16.1 (-12.2, 44.3)	29.4 (-5.8, 64.7)	42.8 (-0.4, 86.1)

Beta=Beta coefficient, CI=Confidence interval

Effect estimates derive from multilevel mixed effects linear regression model, adjusted for maternal education, parity, maternal milk and yoghurt intake, maternal fiber intake, child´s gender and birth weight. The model with all mothers was also adjusted for maternal pre-pregnancy BMI.

[a] Mothers with pre-pregnancy BMI 18.5-24.9 kg/m^2

[b] Mothers with pre-pregnancy BMI ≥25 kg/m^2

For peer review only

Supplementary table 4. Associations between maternal vitamin D intake from foods alone, and from supplements alone, and predicted weight growth trajectories from 3 years to 8 years of age.

	**Preschool age**		**School age**
	**3 years**	**5 years**	**8 years**
	**Beta (95% CI)**	**Beta (95% CI)**	**Beta (95% CI)**
**Maternal vitamin D intake from foods alone**			
All mothers n=66 840			
<5 µg			
5-9.9 µg	-12.4 (-36.7, 11.8)	-17.0 (-56.0, 22.0)	-23.8 (-85.6, 38.0)
10-15 µg	34.8 (-0.8, 70.3)	65.2 (8.1, 122)	111 (20.5, 201)
>15 µg	-5.1 (-66.8, 56.5)	-0.4 (-99.4, 98.6)	6.7 (-150, 163)
Mothers with normal weight [a] n=41 970			
<5 µg			
5-9.9 µg	-15.7 (-42.8, 11.5)	-22.0 (-65.8, 21.8)	-31.4 (-100, 37.9)
10-15 µg	26.4 (-12.7, 65.6)	49.6 (-13.4, 113)	84.3 (-15.3, 184)
>15 µg	-12.4 (-81.0, 56.3)	-10.6 (-121, 99.8)	-8.0 (-183, 167)
Mothers with overweight [b] n=20 080			
<5 µg			
5-9.9 µg	-4.8 (-41.7, 32.1)	-5.1 (-64.6, 54.2)	-5.7 (-99.8, 88.3)
10-15 µg	54.5 (-0.1, 109)	102.4 (14.5, 190)	174.3 (35.1, 313)
>15 µg	12.1 (-81.3, 106)	23.9 (-126, 174)	41.7 (-196, 280)
**Maternal vitamin D intake from supplements alone**			
All mothers n=66 840			
<5 µg			
5-9.9 µg	-4.3 (-31.4, 22.8)	0.9 (-42.7, 44.6)	8.7 (-60.5, 77.9)
10-15 µg	-4.3 (-41.4, 32.8)	-4.5 (-64.3, 55.2)	-4.8 (-99.5, 89.8)
>15 µg	18.9 (-17.2, 55.1)	37.8 (-20.5, 96.0)	66.0 (-26.3, 158)
Mothers with normal weight [a] n=41 970			
<5 µg			

For peer review only

5-9.9 µg	-29.8 (-59.9, 0.3)	-43.0 (-91.5, 5.4)	-62.8 (-140, 13.9)
10-15 µg	-28.7 (-69.3, 11.9)	-44.4 (-110, 21.0)	-67.9 (-171, 35.6)
>15 µg	-2.4 (-41.2, 36.5)	1.9 (-60.7, 64.5)	8.2 (-90.9, 107)
Mothers with overweight [b] ***n=20 080***			
<5 µg			
5-9.9 µg	56.4 (14.8, 98.0)	105 (38.2, 172)	179 (72.3, 285)
10-15 µg	53.5 (-7.3, 114)	90.1 (-7.9, 188)	145 (-10.3, 300)
>15 µg	69.5 (8.8, 130)	123 (25.1, 221)	203 (48.2, 358)

Beta=Beta coefficient, CI=Confidence interval

Effect estimates derive from multilevel mixed effects linear regression model, adjusted for maternal education, parity, maternal milk and yoghurt intake, maternal fiber intake, child´s gender and birth weight. The model with all mothers was also adjusted for maternal pre-pregnancy BMI.

[a] Mothers with pre-pregnancy BMI 18.5-24.9 kg/m^2

[b] Mothers with pre-pregnancy BMI ≥25 kg/m^2

For peer review only

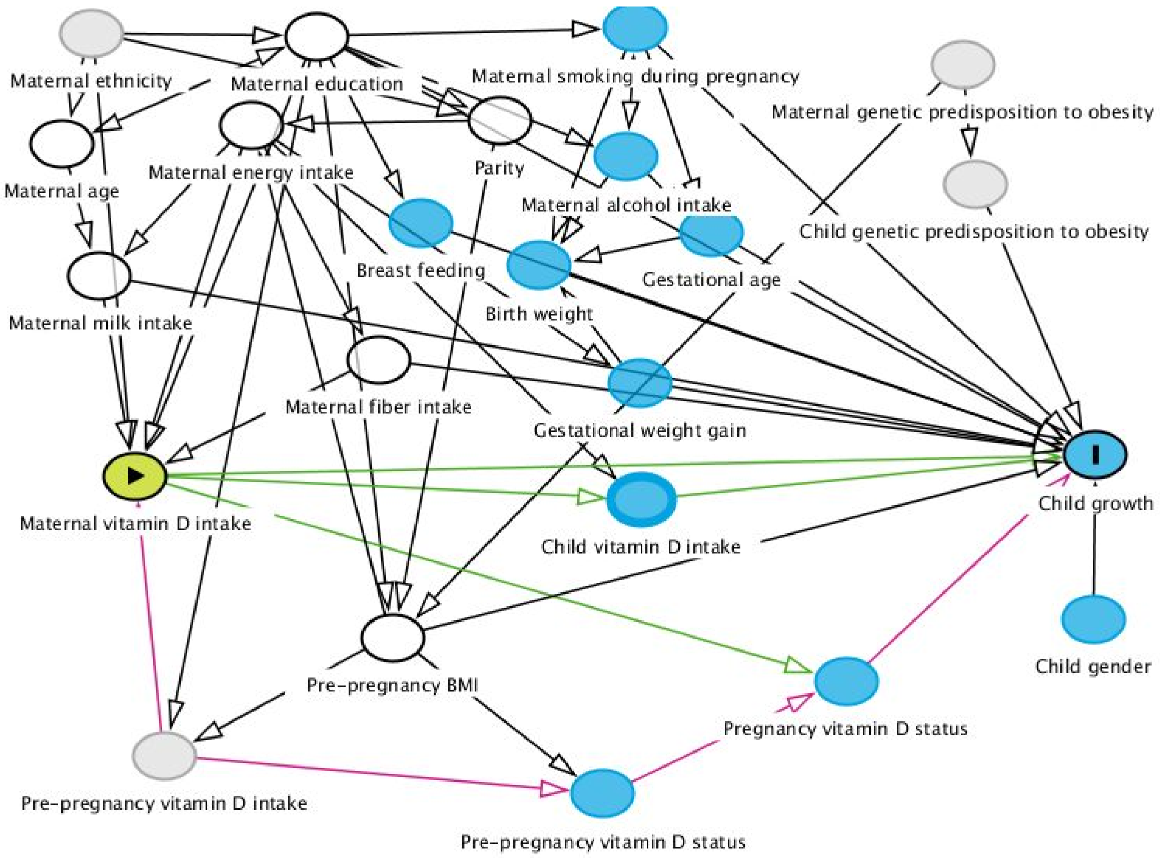

Supplementary figure 1. Directed acyclic graph on the suggested association between, exposure, outcome and covariates [a]. The graph is made using the software DAGitty[b].

Legends: White circle – ancestor of exposure and outcome, adjusted variable; blue circle – ancestor of outcome; grey circle – unobserved variable; black arrow – direct causal effects; green arrow – causal path; pink arrow – biasing path.

[a] Textor J, van der Zander B, Gilthorpe M. S, Liskiewicz M, Ellison G. T. Robust causal inference using directed acyclic graphs: the R package 'dagitty'. International Journal of Epidemiology 45(6):1887-1894, 2016.

[b] (www.dagitty.net/dags.html).

Supplementary table 5. Adjusted change in siblings' weights and heights from 1 month to 2 years, associated with an increase in maternal total vitamin D intake within sibships.

	**Infancy**			**Toddlerhood**		
	**1 month**	**3 months**	**6 months**	**12 months**	**18 months**	**2 years**
**Siblings in the higher maternal vitamin D category**	**Beta (95% CI)**	**Beta (95% CI)**	**Beta (95% CI)**	**Beta (95% CI)**	**Beta (95% CI)**	**Beta (95% CI)**
Weight (g)						
All mothers (n=1216)	22.0 (-28.0, 71.9)	20.4 (-30.0, 70.9)	18.2 (-34.3, 70.7)	13.7 (-46.6, 74.0)	9.2 (-62.5, 80.8)	4.6 (-80.6, 89.8)
Mothers with normal weight [a] (n=837)	3.2 (-56.6, 63.0)	2.0 (-58.2, 62.2)	0.3 (-61.7, 62,2)	-3.3 (-72.8, 66.2)	-6.8 (-87.8, 74.2)	-10.4 (-105, 84.6)
Mothers with overweight [b] (n=344)	31.3 (-57.7, 120)	29.6 (-59.9, 119)	27.0 (-65.1, 119)	21.9 (-81.4, 125)	16.9 (-104, 137)	11.8 (129, 153)
Height (cm)						
All mothers (n=1216)	0.07 (-0.15, 0.29)	0.07 (-0.14, 0.29)	0.07 (-0.14, 0.29)	0.08 (-0.12, 0.28)	0.08 (-0.11, 0.27)	0.08 (-0.11, 0.27)
Mothers with normal weight [a] (n=837)	0.02 (-0.22, 0.27)	0.02 (-0.22, 0.27)	0.03 (-0.21, 0.26)	0.04 (-0.19, 0.26)	0.04 (-0.17, 0.26)	0.05 (-0.16, 0.26)
Mothers with overweight [b] (n=344)	0.11 (-0.26, 0.47)	0.10 (-0.25, 0.46)	0.10 (-0.25, 0.45)	0.09 (-0.24, 0.42)	0.08 (-0.24, 0.40)	0.07 (-0.24, 0.39)

Beta=Beta coefficient, CI=Confidence interval

Effect estimates derived by multilevel mixed effects linear regression model, adjusted for maternal education, parity, maternal milk and yoghurt intake, maternal fiber intake, child´s gender and birth weight. The model with all mothers was also adjusted for maternal pre-pregnancy BMI. The siblings in the lower maternal vitamin D category was used as reference in separate analyses of overall (n=1211), mothers with normal weight (n=845) and with overweight (n=320).

[a] Mothers with pre-pregnancy BMI 18.5-24.9 kg/m^2

[b] Mothers with pre-pregnancy BMI ≥25 kg/m^2

For peer review only

Supplementary table 6. Adjusted changes in siblings' weights and heights from 3 years to 8 years of age, associated with an increase in maternal vitamin D intake within sibships.

	**Preschool age**		**School age**
	**3 years**	**5 years**	**8 years**
**Sibling in the higher maternal vitamin D category**	**Beta (95% CI)**	**Beta (95% CI)**	**Beta (95% CI)**
Weight (in g)			
All mothers (n=1216)	-4.4 (-120, 111)	-22.5 (-204, 159)	-49.6 (-334, 235)
Mothers with normal weight [a] (n=837)	-17.5 (-145, 110)	-31.7 (-230, 166)	-53.0 (-363, 257)
Mothers with overweight [b] (n=344)	1.6 (-187, 191)	-18.7 (-313, 276)	-49.2 (-510, 411)
Height (in cm)			
All mothers (n=1216)	0.09 (-0.10, 0.26)	0.10 (-0.12, 0.31)	0.11 (-0.20, 0.42)
Mothers with normal weight [a] (n=837)	0.07 (-0.14, 0.28)	0.10 (-0.14, 0.34)	0.15 (-0.19, 0.49)
Mothers with overweight [b] (n=344)	0.05 (-0.26, 0.36)	0.01 (-0.34, 0.37)	-0.04 (-0.55, 0.46)

Beta=Beta coefficient, CI=Confidence interval

Effect estimates derived by multilevel mixed effects linear regression model, adjusted for maternal education, parity, maternal milk and yoghurt intake, maternal fiber intake, child´s gender and birth weight. The model with all mothers was also adjusted for maternal pre-pregnancy BMI. The siblings in the lower maternal vitamin D category was used as reference in separate analyses of overall (n=1211), mothers with normal weight (n=845) and with overweight (n=320).

[a] Mothers with pre-pregnancy BMI 18.5-24.9 kg/m^2

[b] Mothers with pre-pregnancy BMI ≥25 kg/m^2

For peer review only

Supplementary table 7. Adjusted changes in siblings' risk of overweight at 3, 5 and 8 years, associated with an increase in maternal total vitamin D intake within sibships.

	**Risk of childhood overweight [a]**
	**OR (95% CI)**
**Sibling in the higher maternal vitamin D category**	
All mothers (n=1216)	
3 years	0.97 (0.75, 1.26)
5 years	0.92 (0.76, 1.12)
8 years	1.03 (0.80, 1.32)
Mothers with normal weight [b] (n=837)	
3 years	0.89 (0.62, 1.28)
5 years	0.92 (0.71, 1.20)
8 years	0.99 (0.70, 1.39)
Mothers with overweight [c] (n=344)	
3 years	1.04 (0.69, 1.57)
5 years	0.90 (0.64, 1.24)
8 years	1.03 (0.68, 1.57)

OR=Odds Ratio, CI=Confidence interval

Effect estimates derived by multilevel mixed effects linear regression model, adjusted for maternal education, parity, maternal milk and yoghurt intake, maternal fiber intake, child´s gender and birth weight. The model with all mothers was also adjusted for maternal pre-pregnancy BMI. The siblings in the lower maternal vitamin D category was used as reference in separate analyses of overall (n=1211), mothers with normal weight (n=845) and with overweight (n=320).

[a] Overweight, including obesity, defined by Cole TJ, Lobstein T. Extended international (IOTF) body mass index cut-offs for thinness, overweight and obesity. Pediatr Obes. 2012;7(4):284-294. doi:10.1111/j.2047-6310.2012.00064.x

[b] Mothers with pre-pregnancy BMI 18.5-24.9 kg/m^2

[c] Mothers with pre-pregnancy BMI ≥25 kg/m^2

For peer review only

Supplementary table 8. Associations between total maternal vitamin D intake and predicted weight growth velocities and height growth velocities from 1 month to 2 years of age.

	**Infancy**			**Toddlerhood**		
**Maternal vitamin D intake**	**1 month**	**3 months**	**6 months**	**12 months**	**18 months**	**2 years**
	**Beta (95% CI)**	**Beta (95% CI)**	**Beta (95% CI)**	**Beta (95% CI)**	**Beta (95% CI)**	**Beta (95% CI)**
All mothers n=66 840						
Weight growth velocities (g/month)						
<5 µg						
5-9.9 µg	-0.13 (-2.11, 1.86)	-0.11 (-2.04, 1.81)	-0.10 (-1.94, 1.75)	-0.06 (-1.75, 1.63)	-0.03 (-1.60, 1.54)	0.003 (-1.48, 1.49)
10-15 µg	-0.51 (-2.96, 1.94)	-0.52 (-2.89, 1.86)	-0.53 (-2.80, 1.74)	-0.55 (-2.63, 1.53)	-0.57 (-2.51, 1.36)	-0.60 (-2.43, 1.23)
>15 µg	-0.25 (-2.70, 2.20)	-0.24 (-2.62, 2.14)	-0.24 (-2.51, 2.04)	-0.22 (-2.31, 1.87)	-0.20 (-2.14, 1.73)	-0.19 (-2.03, 1.65)
Height growth velocities (cm/month)						
<5 µg						
5-9.9 µg	$4x10^{-5}$ (-0.004, 0.004)	$4x10^{-5}$ (-0.004, 0.004)	$3x10^{-5}$ (-0.004, 0.004)	$3x10^{-5}$ (-0.004, 0.004)	$2x10^{-5}$ (-0.003, 0.003)	$2x10^{-5}$ (-0.003, 0.003)
10-15 µg	$7x10^{-5}$ (-0.005, 0.005)	$3x10^{-5}$ (-0.005, 0.005)	$-3x10^{-5}$ (-0.005, 0.005)	$-2x10^{-4}$ (-0.005, 0.004)	$-3x10^{-4}$ (-0.004, 0.004)	$-4x10^{-4}$ (-0.004, 0.003)
>15 µg	$7x10^{-4}$ (-0.004, 0.006)	$7x10^{-4}$ (-0.004, 0.006)	$7x10^{-4}$ (-0.004, 0.005)	$6x10^{-4}$ (-0.004, 0.005)	$5x10^{-4}$ (-0.004, 0.005)	$5x10^{-4}$ (-0.003, 0.004)
Mothers with normal weight [a] n=41 970						
Weight growth velocities (g/month)						
<5 µg						
5-9.9 µg	-1.03 (-3.28, 1.22)	-1.00 (-3.18, 1.18)	-0.96 (-3.04, 1.12)	-0.88 (-2.79, 1.02)	-0.80 (-2.57, 0.96)	-0.73 (-2.40, 0.95)
10-15 µg	-1.97 (-4.69, 0.75)	-1.95 (-4.59, 0.69)	-1.92 (-4.44, 0.60)	-1.86 (-4.17, 0.45)	-1.80 (-3.94, 0.35)	-1.74 (-3.77, 0.29)
>15 µg	-1.17 (-3.86, 1.53)	-1.14 (-3.76, 1.47)	-1.11 (-3.60, 1.38)	-1.05 (-3.34, 1.24)	-0.99 (-3.11, 1.13)	-0.93 (-2.94, 1.08)
Height growth velocities (cm/month)						
<5 µg						
5-9.9 µg	$-8x10^{-5}$ (-0.005, 0.005)	$-8x10^{-5}$ (-0.005, 0.005)	$-8x10^{-5}$ (-0.004, 0.004)	$-8x10^{-5}$ (-0.004, 0.004)	$-8x10^{-5}$ (-0.004, 0.004)	$-8x10^{-5}$ (-0.004, 0.003)
10-15 µg	$-4x10^{-4}$ (-0.006, 0.005)	$-5x10^{-4}$ (-0.006, 0.005)	$-5x10^{-4}$ (-0.006, 0.005)	$-6x10^{-4}$ (-0.006, 0.004)	$-8x10^{-4}$ (-0.005, 0.004)	$-9x10^{-4}$ (-0.005, 0.003)
>15 µg	$7x10^{-4}$ (-0.005, 0.006)	$6x10^{-4}$ (-0.005, 0.006)	$6x10^{-4}$ (-0.005, 0.006)	$-6x10^{-4}$ (-0.004, 0.005)	$-5x10^{-4}$ (-0.004, 0.005)	$-4x10^{-4}$ (-0.004, 0.005)

For peer review only

Mothers with overweight [b] ***n=20 080***						
Weight growth velocities (g/month)						
<5 µg						
5-9.9 µg	1.17 (-1.87, 1.22)	1.18 (-1.68, 4.03)	1.19 (-1.54, 3.92)	1.22 (-1.28, 3.72)	1.25 (-1.07, 3.56)	1.28 (-0.91, 3.47)
10-15 µg	2.23 (-1.54, 6.00)	2.20 (-1.46, 5.85)	2.15 (-1.35, 5.64)	2.05 (-1.15, 5.25)	1.95 (-1.02, 4.92)	1.85 (-0.95, 4.66)
>15 µg	0.68 (-3.29, 4.65)	0.72 (-3.13, 4.57)	0.78 (-2.90, 4.45)	0.89 (-2.48, 4.26)	1.01 (-2.11, 4.13)	1.13 (-1.83, 4.08)
Height growth velocities (cm/month)						
<5 µg						
5-9.9 µg	$-3x10^{-4}$ (-0.006, 0.006)	$-2x10^{-4}$ (-0.006, 0.006)	$-2x10^{-4}$ (-0.006, 0.006)	$-1x10^{-4}$ (-0.005, 0.005)	$4x10^{-5}$ (-0.005, 0.005)	$4x10^{-5}$ (-0.005, 0.005)
10-15 µg	$6x10^{-4}$ (-0.007, 0.008)	$6x10^{-4}$ (-0.007, 0.008)	$6x10^{-4}$ (-0.007, 0.008)	$6x10^{-4}$ (-0.006, 0.007)	$5x10^{-4}$ (-0.006, 0.007)	$6x10^{-4}$ (-0.005, 0.006)
>15 µg	$-6x10^{-4}$ (-0.009, 0.008)	$-5x10^{-4}$ (-0.009, 0.008)	$-4x10^{-4}$ (-0.008, 0.007)	$-3x10^{-4}$ (-0.007, 0.007)	$-1x10^{-4}$ (-0.007, 0.006)	$-5x10^{-5}$ (-0.006, 0.006)
Beta=Beta coefficient, CI=Confidence interval						
Effect estimates derive from multilevel mixed effects linear regression model, adjusted for maternal education, parity, maternal milk and yoghurt intake, maternal fiber intake, child´s gender and birth weight. The model with all mothers was also adjusted for maternal pre-pregnancy BMI.						
[a] Mothers with pre-pregnancy BMI 18.5-24.9 kg/m^2						
[b] Mothers with pre-pregnancy BMI ≥25 kg/m^2						

For peer review only

Supplementary table 9. Associations between total maternal vitamin D intake and predicted weight growth velocities and height growth velocities from 3 years to 8 years of age.

	Preschool age		School age
	**3 years**	**5 years**	**8 years**
**Maternal vitamin D intake**	**Beta (95% CI)**	**Beta (95% CI)**	**Beta (95% CI)**
All mothers n=66 840			
Weight growth velocities (g/month)			
<5 µg			
5-9.9 µg	0.07 (-1.39, 1.52)	0.20 (-1.71, 2.12)	0.40 (-2.80, 3.61)
10-15 µg	-0.64 (-2.44, 1.15)	-0.73 (-3.09, 1.63)	-0.86 (-4.81, 3.08)
>15 µg	-0.16 (-1.96, 1.64)	-0.10 (-2.46, 2.26)	-0.007 (-3.95, 3.93)
Height growth velocities (cm/month)			
<5 µg			
5-9.9 µg	$4x10^{-6}$ (-0.003, 0.003)	$-4x10^{-5}$ (-0.004, 0.004)	$-5x10^{-5}$ (-0.007, 0.007)
10-15 µg	$-7x10^{-4}$ (-0.004, 0.003)	-0.001 (-0.006, 0.004)	-0.002 (-0.010, 0.006)
>15 µg	$3x10^{-4}$ (-0.003, 0.004)	$9x10^{-5}$ (-0.005, 0.005)	$-3x10^{-4}$ (-0.009, 0.008)
Mothers with normal weight [a] n=41 970			
Weight growth velocities (g/month)			
<5 µg			
5-9.9 µg	-0.57 (-2.21, 1.07)	-0.26 (-2.42, 1.91)	-0.21 (-3,42, 3.84)
10-15 µg	-1.61 (-3.60, 0.38)	-1.37 (-3.99, 1.25)	-1.00 (-5.39, 3.39)
>15 µg	-0.80 (-2.78, 1.17)	-0.56 (-3.15, 2.04)	-0.18 (-4.52, 4.15)
Height growth velocities (cm/month)			
<5 µg			
5-9.9 µg	$-8x10^{-5}$ (-0.004, 0.003)	$-7x10^{-5}$ (-0.005, 0.004)	$-7x10^{-5}$ (-0.008, 0.008)
10-15 µg	-0.001 (-0.005, 0.003)	-0.002 (-0.007, 0.004)	-0.002 (-0.011, 0.007)
>15 µg	$3x10^{-4}$ (-0.004, 0.004)	$1x10^{-4}$ (-0.005, 0.006)	$-2x10^{-4}$ (-0.009, 0.009)

For peer review only

**Mothers with overweight [b]**<br>**n=20 080**			
Weight growth velocities (g/month)			
<5 µg			
5-9.9 µg	1.33 (-0.81, 3.48)	1.45 (-1.39, 4.29)	1.62 (-3.14, 6.38)
10-15 µg	1.66 (-1.10, 4.41)	1.26 (-2.37, 4.89)	0.67 (-5.42, 6.77)
>15 µg	1.36 (-1.54, 4.26)	1.82 (-2.00, 5.64)	2.52 (-3.88, 8.92)
Height growth velocities (cm/month)			
<5 µg			
5-9.9 µg	$2x10^{-4}$ (-0.004, 0.005)	$5x10^{-4}$ (-0.005, 0.006)	0.001 (-0.009, 0.011)
10-15 µg	$5x10^{-4}$ (-0.005, 0.006)	$4x10^{-4}$ (-0.007, 0.008)	$2x10^{-4}$ (-0.013, 0.013)
>15 µg	$4x10^{-4}$ (-0.006, 0.006)	0.001 (-0.007, 0.009)	0.002 (-0.011, 0.015)

Beta=Beta coefficient, CI=Confidence interval

Effect estimates derive from multilevel mixed effects linear regression model, adjusted for maternal education, parity, maternal milk and yoghurt intake, maternal fiber intake, child´s gender and birth weight. The model with all mothers was also adjusted for maternal pre-pregnancy BMI.

[a] Mothers with pre-pregnancy BMI 18.5-24.9 kg/m^2

[b] Mothers with pre-pregnancy BMI ≥25 kg/m^2

For peer review only

Supplementary table 10. Associations (OR and 95% CI) between total maternal vitamin D intake and risk of childhood overweight at 3 years, 5 years and 8 years, using reported anthropometrics.

	**Risk of childhood overweight [a]**
**Maternal vitamin D intake**	**OR (95% CI)**
All mothers	
**3 years *(n=34 438)***	
<5 µg	
5-9.9 µg	1.00 (0.93, 1.08)
10-15 µg	0.96 (0.87, 1.06)
>15 µg	0.89 (0.81, 0.98)
**5 years (n=29 301)**	
<5 µg	
5-9.9 µg	0.97 (0.88, 1.06)
10-15 µg	0.97 (0.87, 1.08)
>15 µg	0.95 (0.85, 1.06)
**8 years (n=22 447)**	
<5 µg	
5-9.9 µg	1.10 (1.00, 1.20)
10-15 µg	1.05 (0.94, 1.17)
>15 µg	1.06 (0.95, 1.18)
Mothers with normal weight [b]	
**3 years *(n=22 863)***	
<5 µg	
5-9.9 µg	0.98 (0.89, 1.08)
10-15 µg	0.95 (0.84, 1.07)
>15 µg	0.87 (0.77, 0.98)
**5 years (n=19 767)**	
<5 µg	
5-9.9 µg	0.98 (0.87, 1.10)
10-15 µg	0.94 (0.81, 1.08)
>15 µg	0.90 (0.78, 1.04)
**8 years (n=15 171)**	
<5 µg	
5-9.9 µg	1.03 (0.91, 1.16)
10-15 µg	0.96 (0.83, 1.11)
>15 µg	1.02 (0.89, 1.18)
Mothers with overweight [c]	
**3 years (n=10 633)**	
<5 µg	
5-9.9 µg	1.02 (0.90, 1.15)
10-15 µg	0.96 (0.82, 1.13)
>15 µg	0.88 (0.74, 1.04)
**5 years (n=8709)**	
<5 µg	
5-9.9 µg	0.94 (0.82, 1.08)

For peer review only

10-15 µg	1.02 (0.86, 1.21)
>15 µg	0.98 (0.82, 1.17)
**8 years (n=6661)**	
<5 µg	
5-9.9 µg	1.22 (1.06, 1.40)
10-15 µg	1.19 (1.00, 1.42)
>15 µg	1.11 (0.93, 1.33)

OR=Odds Ratio, CI=Confidence interval

Effect estimates derived by logistic regression, adjusted for maternal education, parity, maternal milk and yoghurt intake, maternal fiber intake and child´s gender. The model with all mothers was also adjusted for maternal pre-pregnancy BMI.

[a] Overweight, including obesity, defined by Cole TJ, Lobstein T. Extended international (IOTF) body mass index cut-offs for thinness, overweight and obesity. Pediatr Obes. 2012;7(4):284-294. doi:10.1111/j.2047-6310.2012.00064.x

[b] Mothers with pre-pregnancy BMI 18.5-24.9 kg/m^2

[c] Mothers with pre-pregnancy BMI ≥25 kg/m^2

For peer review only

For peer review only

Reporting checklist for cohort study.

Based on the STROBE cohort guidelines.

Instructions to authors

Complete this checklist by entering the page numbers from your manuscript where readers will find each of the items listed below.

Your article may not currently address all the items on the checklist. Please modify your text to include the missing information. If you are certain that an item does not apply, please write "n/a" and provide a short explanation.

Upload your completed checklist as an extra file when you submit to a journal.

In your methods section, say that you used the STROBE cohortreporting guidelines, and cite them as:

von Elm E, Altman DG, Egger M, Pocock SJ, Gotzsche PC, Vandenbroucke JP. The Strengthening the Reporting of Observational Studies in Epidemiology (STROBE) Statement: guidelines for reporting observational studies.

		Reporting Item	Page Number
**Title and abstract**			
Title	#1a	Indicate the study’s design with a commonly used term in the title or the abstract	2
Abstract	#1b	Provide in the abstract an informative and balanced summary of what was done and what was found	2

For peer review only

Introduction

Background / rationale	#2	Explain the scientific background and rationale for the investigation being reported	4-5
Objectives	#3	State specific objectives, including any prespecified hypotheses	5

Methods

Study design	#4	Present key elements of study design early in the paper	7-8
Setting	#5	Describe the setting, locations, and relevant dates, including periods of recruitment, exposure, follow-up, and data collection	5-6
Eligibility criteria	#6a	Give the eligibility criteria, and the sources and methods of selection of participants. Describe methods of follow-up.	6
Eligibility criteria	#6b	For matched studies, give matching criteria and number of exposed and unexposed	n/a
Variables	#7	Clearly define all outcomes, exposures, predictors, potential confounders, and effect modifiers. Give diagnostic criteria, if applicable	6-8
Data sources / measurement	#8	For each variable of interest give sources of data and details of methods of assessment (measurement). Describe comparability of	6-8

For peer review only

		assessment methods if there is more than one group. Give information separately for for exposed and unexposed groups if applicable.	
Bias	#9	Describe any efforts to address potential sources of bias	7
Study size	#10	Explain how the study size was arrived at	6
Quantitative variables	#11	Explain how quantitative variables were handled in the analyses. If applicable, describe which groupings were chosen, and why	7-9
Statistical methods	#12a	Describe all statistical methods, including those used to control for confounding	
8-9			
Statistical methods	#12b	Describe any methods used to examine subgroups and interactions	9
Statistical methods	#12c	Explain how missing data were addressed	6
Statistical methods	#12d	If applicable, explain how loss to follow-up was addressed	7
Statistical methods	#12e	Describe any sensitivity analyses	
9			

Results

For peer review only

Participants	#13a	Report numbers of individuals at each stage of study—eg numbers potentially eligible, examined for eligibility, confirmed eligible, included in the study, completing follow-up, and analysed. Give information separately for for exposed and unexposed groups if applicable.	6+10
Participants	#13b	Give reasons for non-participation at each stage	n/a
Participants	#13c	Consider use of a flow diagram	
n/a			
Descriptive data	#14a	Give characteristics of study participants (eg demographic, clinical, social) and information on exposures and potential confounders. Give information separately for exposed and unexposed groups if applicable.	10-11
Descriptive data	#14b	Indicate number of participants with missing data for each variable of interest	
n/a			
Descriptive data	#14c	Summarise follow-up time (eg, average and total amount)	
Supplementary table 1			

For peer review only

Outcome data	#15	Report numbers of outcome events or summary measures over time. Give information separately for exposed and unexposed groups if applicable.	
19			
Main results	#16a	Give unadjusted estimates and, if applicable, confounder-adjusted estimates and their precision (eg, 95% confidence interval). Make clear which confounders were adjusted for and why they were included	14-20
Main results	#16b	Report category boundaries when continuous variables were categorized	14-20
Main results	#16c	If relevant, consider translating estimates of relative risk into absolute risk for a meaningful time period	
n/a			
Other analyses	#17	Report other analyses done—eg analyses of subgroups and interactions, and sensitivity analyses	12-13, 18-19, Supplementary table 10

Discussion

Key results	#18	Summarise key results with reference to study objectives	20-21

For peer review only

Limitations	#19	Discuss limitations of the study, taking into account sources of potential bias or imprecision. Discuss both direction and magnitude of any potential bias.	21
Interpretation	#20	Give a cautious overall interpretation considering objectives, limitations, multiplicity of analyses, results from similar studies, and other relevant evidence.	22
Generalisability	#21	Discuss the generalisability (external validity) of the study results	22
**Other Information**			
Funding	#22	Give the source of funding and the role of the funders for the present study and, if applicable, for the original study on which the present article is based	23

None The STROBE checklist is distributed under the terms of the Creative Commons Attribution License CC-BY. This checklist can be completed online using https://www.goodreports.org/, a tool made by the EQUATOR Network in collaboration with Penelope.ai

For peer review only

BMJ Open

Maternal vitamin D intake and BMI during pregnancy in relation to child´s growth and weight status from birth to 8 years - a large national cohort study

Journal:	*BMJ Open*
Manuscript ID	bmjopen-2021-048980.R1
Article Type:	Original research
Date Submitted by the Author:	25-Aug-2021
Complete List of Authors:	Amberntsson, Anna; University of Gothenburg, Institute of Medicine<br>Papadopoulou, Eleni; Norwegian Institute of Public Health<br>Winkvist, Anna; University of Gothenburg, Institute of Medicine<br>Lissner, Lauren; University of Gothenburg, Institute of Medicine<br>Meltzer, Helle Margrete; Norwegian Institute of Public Health<br>Brantsaeter, Anne Lise; Norwegian Institute of Public Health<br>Augustin, Hanna; University of Gothenburg, Institute of Medicine
**Primary Subject Heading**:	Nutrition and metabolism
Secondary Subject Heading:	Epidemiology
Keywords:	EPIDEMIOLOGY, NUTRITION & DIETETICS, PREVENTIVE MEDICINE, PUBLIC HEALTH, SOCIAL MEDICINE

SCHOLARONE™
Manuscripts

I, the Submitting Author has the right to grant and does grant on behalf of all authors of the Work (as defined in the below author licence), an exclusive licence and/or a non-exclusive licence for contributions from authors who are: i) UK Crown employees; ii) where BMJ has agreed a CC-BY licence shall apply, and/or iii) in accordance with the terms applicable for US Federal Government officers or employees acting as part of their official duties; on a worldwide, perpetual, irrevocable, royalty-free basis to BMJ Publishing Group Ltd ("BMJ") its licensees and where the relevant Journal is co-owned by BMJ to the co-owners of the Journal, to publish the Work in this journal and any other BMJ products and to exploit all rights, as set out in our licence.

The Submitting Author accepts and understands that any supply made under these terms is made by BMJ to the Submitting Author unless you are acting as an employee on behalf of your employer or a postgraduate student of an affiliated institution which is paying any applicable article publishing charge ("APC") for Open Access articles. Where the Submitting Author wishes to make the Work available on an Open Access basis (and intends to pay the relevant APC), the terms of reuse of such Open Access shall be governed by a Creative Commons licence – details of these licences and which Creative Commons licence will apply to this Work are set out in our licence referred to above.

Other than as permitted in any relevant BMJ Author's Self Archiving Policies, I confirm this Work has not been accepted for publication elsewhere, is not being considered for publication elsewhere and does not duplicate material already published. I confirm all authors consent to publication of this Work and authorise the granting of this licence.

For peer review only

Maternal vitamin D intake and BMI during pregnancy in relation to child´s growth and weight status from birth to 8 years - a large national cohort study

Anna Amberntsson[1], Eleni Papadopoulou[2], Anna Winkvist[1], Lauren Lissner[1], Helle Margrete Meltzer[2], Anne-Lise Brantsæter[2], Hanna Augustin[1]

1. Institute of Medicine, Sahlgrenska Academy, University of Gothenburg

2. Norwegian Institute of Public Health, Oslo, Norway

Corresponding author: Anna Amberntsson; anna.amberntsson@gu.se

For peer review only

For peer review only - http://bmjopen.bmj.com/site/about/guidelines.xhtml

Abstract

Objectives: To examine the associations between maternal vitamin D intake and childhood growth and risk of overweight up to 8 years. We further examined the effect modification by maternal pre-pregnancy BMI.

Design: Prospective population-based pregnancy cohort study

Setting: The Norwegian Mother, Father and Child Cohort Study.

Participants: In total, 58 724 mothers and 66 840 singleton children, with information on maternal vitamin D intake during pregnancy and minimum one postnatal anthropometric measurement.

Outcome measures: Predicted weight and height growth trajectories and velocities from 1 month to 8 years, rapid growth during infancy and toddlerhood, and risk of overweight in pre-school and school age.

Results: Overall, maternal vitamin D intake was associated with lower weight trajectory, lower odds of rapid weight growth and higher odds of childhood overweight. In children of mothers with pre-pregnancy normal weight, maternal vitamin D intake was negatively associated with weight trajectory and lower odds ratio of a rapid weight growth during the first year, compared to reference (<5µg/day). Children of mothers with normal weight, with maternal vitamin D intakes of 10-15 and >15 µg/day, also had 0.86 (95% CI 0.77, 0.97) and 0.88 (95% CI 0.79, 0.99) lower odds for overweight at 3 years, compared to reference. In contrast, in children of mothers with pre-pregnancy overweight (BMI ≥25 kg/m^2), vitamin D intake was positively associated with weight trajectory. Children of mothers with overweight, with maternal vitamin D intake of 5-9.9 µg/day, also had (1.09 (95% CI 1.01, 1.18) and 1.12 (95% CI 1.02, 1.23)) higher odds for overweight at 5 years and 8 years, compared to reference.

Conclusions: Maternal vitamin D intake affects postnatal growth and is inversely associated with childhood overweight in children of mothers with normal weight. Associations between maternal vitamin D intake and child growth and risk of overweight varied by pre-pregnancy BMI.

For peer review only

Article Summary

- To our knowledge, this is the first study to investigate the association between maternal vitamin D intake during pregnancy and postnatal growth
- The large, nationwide study population is a major strength
- Maternal vitamin D intake was estimated from both foods and supplements by a validated food frequency questionnaire
- A limitation is self-reported maternal data and parental-reported growth data of the child

Key words: The Norwegian Mother, Father and Child Cohort Study, weight, height

For peer review only

Introduction

Vitamin D has received much attention over the last few decades and its role in human physiology seems to extend beyond bone health [1]. The human skin can synthesize a precursor of the vitamin when exposed to ultra violet-B rays of wavelength 290-315 nm [2]. At northern latitudes, this only occurs from April to October. Therefore, intake of vitamin D from foods and supplements becomes important during winter months in many countries, including the Nordics [3]. Vitamin D occurs naturally in a few foods, such as fatty fish, but is presently added to many fortified foods in the form of vitamin D_3[2]. The more uncommon form vitamin D_2 is only present in some plants and mushrooms [4]. Synthesized or ingested vitamin D (vitamin D_3 and vitamin D_2) is hydroxylated in the liver to 25-hydroxyvitamin D (25OHD), an established blood biomarker of vitamin D status. Further, 25OHD undergoes another hydroxylation primarily in the kidneys to form the physiologically active 1,25-dihydroxyvitamin D (1,25OH_2D) [4]. The circulating concentration of 25OHD has shown to be inversely associated with BMI, suggesting that individuals with obesity have a decreased bioavailability of vitamin D [5].

Worldwide, the prevalence of vitamin D insufficiency (here defined as 25OHD <50 nmol/L) is high among pregnant women [6]. In some regions, such as South-East Asia and Western Pacific, the prevalence of vitamin D insufficiency were as high as 80% [6]. In contrast, in Europe and Eastern Mediterranean, the prevalence of vitamin D insufficiency were 57% and 46%, respectively [6]. In the Nordic countries, up to 28% of the pregnant fair-skinned European women have vitamin D insufficiency, but among non-fair skinned European women living in the Nordic countries, the prevalence is rising between 69% and 82% [7-9]. Therefore, on a population level, vitamin D insufficiency and deficiency in pregnancy are a relevant public health issue affecting the future generations. Pregnancy is a critical period where fetal exposure to poor conditions can affect later outcomes in the offspring [10, 11]. Maternal vitamin D status, assessed as serum or plasma 25OHD concentrations, has been suggested as a potential modifiable early-life risk factor for development of childhood obesity [12]. Early rapid growth can increase the risk for childhood obesity, and both conditions are associated with several comorbidities in both childhood and adulthood, and is likely to follow the child through life [13-16]. In 2018, globally the prevalence of children with overweight and obesity under 5 years of age had risen to 40 million [17].

For peer review only - http://bmjopen.bmj.com/site/about/guidelines.xhtml

Vitamin D contributes to the embryo implantation and is also important for placental function [18]. The maternal 25OHD easily passes the placenta and is further hydroxylated into 1,25OH$_2$D by the fetal kidneys, providing the fetus with vitamin D [19]. Vitamin D deficiency during pregnancy increases risk of developing rickets in the child [20]. Maternal vitamin D deficiency has also been associated with several other adverse neonatal outcomes, such as small for gestational age [21]. Associations have been found between low maternal vitamin D status and an increased risk of the child being born small for gestational age and with a lower birth weight [22], as well as accelerated growth in weight and height during infancy [23]. Maternal vitamin D status has also been inversely associated with childhood adiposity at 4 and 6 years [24] and childhood insulin resistance at 9.5 years [25]. Further, associations of an increased risk of childhood overweight at 12 months of age was seen in children of mothers with low vitamin D status during pregnancy [26, 27]. However, some studies have found opposite results, i.e. that poor maternal vitamin D status was associated with reduced child growth [28], while others have reported null associations with childhood adiposity [25, 29, 30] and growth [26, 29]. Even though the vitamin D intake from food and supplements is a determinant of vitamin D status , the role of maternal vitamin D intake during pregnancy has been less studied, and the limited evidence regarding fetal growth is discordant [31, 32]. Associations between maternal vitamin D intake in pregnancy and postnatal growth and overweight have not yet been explored. Since vitamin D intake affects vitamin D status [4], and due to the decreased bioavailability of vitamin D in obesity (due to volumetric dilution of 25OHD [5]), it is possible that such an association could be modified by maternal pre-pregnancy BMI.

Our objectives were to assess the associations between maternal vitamin D intake during pregnancy and childhood weight and height growth trajectories and velocities, rapid growth and risk of overweight up to 8 years, in a large, prospective pregnancy cohort study. Further, we aimed to examine maternal pre-pregnancy BMI as an effect modifier.

Method

Study population

Our study draws resources from the Norwegian Mother, Father and Child Cohort Study (MoBa), a population-based pregnancy cohort study conducted by the Norwegian Institute of Public Health (NIPH) [33]. Participants were recruited from all over Norway during 1999-2008 and invited at the time of their first ultrasound scanning. The women consented to

For peer review only

participation in 41% of the pregnancies. The study now includes 95 200 mothers, 75 200 fathers and 114 500 children. The current study is based on version 12 of the quality-assured data files released for research in January 2019. The establishment of MoBa and initial data collection was based on a license from the Norwegian Data Protection Agency and approval from The Regional Committees for Medical and Health Research Ethics. The MoBa cohort is now based on regulations related to the Norwegian Health Registry Act. The current study was approved by The Regional Committees for Medical and Health Research Ethics (2019/770 REK Nord).

Questionnaires were sent out to the participating women in pregnancy week 15, week 22 and week 30. The questionnaires in week 15 and 30 requested background information, such as education and parity, whereas the questionnaire in week 22 was a food frequency questionnaire (FFQ). After delivery, follow-up of the participants by questionnaires was conducted at six different time points; 6 months, 18 months, 36 months, 5 years, 7 years and 8 years [33, 34]. Participants in MoBa received all questionnaires in Norwegian, and lack of language skills was the only exclusion criterion. However, the questionnaires are now available in English translation on the NIPH website [35]. The MoBa database is linked to the Medical Birth Registry of Norway (MBRN), which is a national health registry containing information about all births in Norway [36].

Exclusion criteria for this study were stillbirth, multiple gestation, malformation and chromosomal abnormalities. Of the eligible 105 344 mother-child pairs, further exclusion was done due to no available data on vitamin D intake during pregnancy, child´s gender and birth weight and length, and implausible maternal energy intakes (<1075 kcal and >4780 kcal per day) [37]. The final study population included 66 840 mother-child pairs (58 724 mothers and 66 840 children) with available information on all covariates and at least one postnatal measurement on weight and length/height. There are more children than mothers because 22 039 of the children were siblings. The retention rate of our study population by follow-up is presented in Supplementary Table 1.

Patient and public involvement

Neither patients nor the public were involved in the design and conduct of this study. The cohort is ongoing and information about research projects and results from MoBa are available to participants, researchers and the public on the NIPH website [38].

For peer review only - http://bmjopen.bmj.com/site/about/guidelines.xhtml

Maternal vitamin D intake

Maternal diet during pregnancy was assessed by a validated semi-quantitative Food Frequency Questionnaire (FFQ), during gestational week 22. This FFQ was used in the MoBa study from the year 2002 and onwards [37]. Pregnant women were asked to report how often they consumed 225 different food and beverage items and their supplement use since becoming pregnant. The FFQ was tailored to capture dietary habits and supplement use during the first half of pregnancy. Of all the participating women in MoBa, 93% answered the FFQ [37]. A validation study in a subset of 119 MoBa participants demonstrated satisfactory validity for FFQ intakes and fair ability of the FFQ to rank individuals according to food, energy and nutrient intakes using a 4-day weighed food dairy and biological markers of food intake as reference methods [39, 40]. The results showed that vitamin D supplement use was the dominant predictor of plasma 25OHD, whereas vitamin D from foods was not associated with 25OHD. The majority of participants were recruited in the months with low sun exposure (October to April), but even within this range seasonal variation in plasma 25OHD could be observed [40]. The Spearman's rank correlation coefficient between maternal vitamin D intake and plasma 25OHD concentration was $r=0.32$ ($p<0.01$) for all participants in the validation study ($n=119$) and $r=0.45$ ($p<0.01$) when only individuals recruited during October to April ($n=81$) were included [40]. Total vitamin D (from foods and supplements), vitamin D from foods alone and vitamin D from supplements alone were estimated from the FFQ and used in our study.

Maternal vitamin D intake was categorized according to national recommended intakes at the time of MoBa recruitment as well as according to current recommendations [41]. Total vitamin D intake (from foods and supplements), and vitamin D intake from supplements alone, were categorized into; <5 µg/day, 5-9.9 µg/day, 10-15 µg/day and >15 µg/day. The categories for vitamin D intake from foods alone were; <2.5 µg/day, 2.5-4.9 µg/day, 5.0-7.5 µg/day and >7.5 µg/day.

Childhood anthropometry

Measurements of weight and height and the dates of the measurements of the children were obtained at eleven ages (6 weeks, 3 months, 6 months and 8 months and 1 year, 1.5 years, 2 years, 3 years, 5 years, 7 years and 8 years) by six questionnaires. During the first 18 months postpartum, the mothers were asked to report the child´s documented anthropometric measures and dates from the health card, but no specification was made for measurements onwards.

For peer review only - http://bmjopen.bmj.com/site/about/guidelines.xhtml

There were on average seven reported measurements of weight and height respectively per child. From the available reported growth measurements, individual growth trajectories for weight and height were predicted using the Jenss-Bayley growth curve model [42, 43] with the Saemix algorithm [43] applied. Hence, the predicted measures of weight and height were obtained on the dates of the reported anthropometrics. Implausible values of the reported anthropometrics were identified and removed if they deviated ≥ 3 standard deviations (SD) from the predicted value, derived from the growth curve model. About 3% (n=50 331) of the weight and height values were excluded as implausible.

Outcomes

After exclusion of implausible reported anthropometric values, individual growth trajectories of weight and height, as well as weight growth velocity (g/month) and height growth velocity (cm/month) were predicted at eleven different age points (1 month, 3 months, 6 months, 9 months, 1 year, 1.5 years, 2 years, 3 years, 5 years, 7 years and 8 years). The trajectories and velocities were predicted at the exact age points (the exact day the child turned 1 month, 3 months, etc.), separately for girls and boys, in order to compensate for the loss to follow-up of anthropometric measures. By not including birth weight and length in the models of growth, the effect of maternal vitamin D intake on growth could be assessed independently of its potential effect on birth size [31, 32], as birth size might influence the estimated growth trajectories.

Rapid weight and height growth in infancy and toddlerhood (up to 2 years) were evaluated using conditional growth modeling, an approach to obtain growth measures of weight and height independent of their prior measurements [44]. Reported anthropometrics were used for the calculation of conditional weight and height at 12 months and at 2 years. Standardized residuals were calculated by a regression of the anthropometric measure of interest, adjusting for its prior measurements. Rapid weight or height growth were defined as the residual deviating >1 SD from the expected value. All other children were defined as non-rapid growers and were used as the reference.

Children's BMI values were calculated at pre-school age (3 years and 5 years) and school age (8 years) using the predicted values from the growth model. By using the extended International Obesity Task Force (IOTF) gender-specific cut offs [45], overweight and obesity were defined. The outcome childhood overweight includes obesity (further denoted as

For peer review only - http://bmjopen.bmj.com/site/about/guidelines.xhtml

overweight). The BMI cut-offs used for categorization at the different ages are presented in Supplementary Table 2.

Statistical analysis

Differences in characteristics of the study population by categories of maternal vitamin D intake were investigated using Chi-2 test for categorical variables and ANOVA for continuous variables. The association between maternal vitamin D intake and predicted weight and height growth trajectories and velocities at the eleven time points were examined by multilevel mixed effects linear regression model, with a random intercept by child and random slope for age. The covariates were included as fixed in the mixed effect models. Logistic regression was used to investigate the association between total maternal vitamin D intake and the risk to experience rapid growth in infancy and toddlerhood, and risk of overweight in pre-school and school ages. The lowest categories of maternal vitamin D intake was used as the reference group (for total intake and for supplementary intake <5 μg/day), and for food intake <2.5 μg/day). Our primary exposure was total maternal vitamin D intake and in secondary analyses, we examined intakes from food and supplements separately.

By drawing a directed acyclic graph, hypotheses about the causal paths were set and potential confounders identified (Supplementary Figure 1). Based on the pathways in the graph, models were adjusted for maternal education as a proxy for socio-economic position, parity, maternal milk and yoghurt intake, maternal fiber intake, maternal pre-pregnancy BMI and child´s gender. Maternal age, maternal energy intake and birth weight were also considered but not included in the final models, as these variables did not change the estimated effect. Since some mothers participated in more than one mother-child pair, all regression models were also adjusted for random effects of sibling clusters. Potential interaction variables tested were maternal pre-pregnancy BMI (in kg/m^2) [46, 47] and birth weight [32, 48]. Interaction were considered if $p<0.100$ for the included interaction variable. The only significant interaction variable was pre-pregnancy BMI ($p<0.001$). Therefore, results are presented for all mothers, and separately for children of mothers with pre-pregnancy BMI 18.5-24.9 kg/m^2 (further denoted as mothers with normal weight), and for children of mothers with pre-pregnancy BMI ≥25 kg/m^2 (further denoted as mothers with overweight). Maternal age was divided into four categories (<20, 20–29, 30–39, ≥40 years). Maternal education was divided into three categories (<13, 13-16 and >16 years). Parity was categorized as primiparous or multiparous, for women without and with children from

For peer review only - http://bmjopen.bmj.com/site/about/guidelines.xhtml

previous than the index pregnancies, respectively. Maternal pre-pregnancy body mass index (BMI) was divided into four categories (<18.5, 18.5–24.9, 25–29.9, and ≥30 kg/m^2), according to the World Health Organization [49]. Smoking during pregnancy was categorized as never and ever.

Three sensitivity analyses were performed: (1) using the reported measures of weight and length/height instead of the predicted anthropometrics, (2) after excluding mothers with total vitamin D intakes >90th percentile (18.8 µg/day), (3) adjustment for birth weight in the childhood overweight models.

As an approach to estimate causal effects, discordant sibling analysis was conducted, with the purpose to control for unmeasured familial confounding [50, 51]. In total, there were 22 039 siblings identified in the study subpopulation. For all women who participated with more than one pregnancy, mean maternal total vitamin D intake by family was calculated, as well as each sibling's deviation from its family mean. A cut off at 5 µg/day was used, as this corresponds to the range of the maternal vitamin D categories. Discordant siblings were defined as having a maternal vitamin D intake deviating more than, or less than the cut off from its family mean. Finally, weight and height growth trajectories, and the risk of overweight at 3, 5 and 8 years, were compared between siblings categorized as discordant (n= 2346). The sibling in the lower vitamin D category was used as a reference.

The main analyses were performed using Stata version 16 statistical software (Stata Corporation, College Station, Texas, USA), and RStudio version 1.2.5042 was used for the growth models.

Results

Maternal characteristics and vitamin D intake

For mothers taking a vitamin D supplement, the supplement contributed overall with 68% of the total vitamin D intake. Total maternal vitamin D intake was positively associated with maternal age, education, vitamin D supplement use in pregnancy, gestational weight gain and maternal energy intake, and negatively associated with parity, pre-pregnancy BMI and maternal smoking in pregnancy (Table 1). In 31% of the pregnancies, the mother had a BMI ≥25 kg/m^2. The median (25th−75th centiles) total vitamin D intake from foods and supplements was 7.2 µg/day (4.2–12.1 µg/day) for the included participants and 6.7 µg/day (3.9–11.7 µg/day) for the excluded group (n=13 826, p<0.001) (data not shown). The median

For peer review only - http://bmjopen.bmj.com/site/about/guidelines.xhtml

For peer review only

vitamin D intake from foods alone was 3.1 µg/day (2.0-4.4 µg/day) for the included participants and 3.1 µg/day (2-4.5 µg/day) for the excluded group, with available information on vitamin D intake (n=16 872 mothers, p=0.589).

For peer review only

Table 1. Study population characteristics by categories of total maternal vitamin D intake during pregnancy (n=66 840)

	**Vitamin D intake <5µg/day**		**Vitamin D intake 5-9.9 µg/day**		**Vitamin D intake 10-15 µg/day**		**Vitamin D intake >15 µg/day**		**All**		
	N=21 748		N=22 718		N=11 143		N=11 231		N=66 840		
	**N**	**%**	**N**	**%**	**N**	**%**	**N**	**%**	**N**	**%**	**P-value[a]**
**Maternal age (years)**											<0.001
<20	194	0.9	135	0.6	83	0.8	82	0.7	494	0.7	
20-29	9566	44	9740	42.9	4684	42.0	4415	39.3	28 405	42.5	
30-39	11 622	53.4	12 449	54.9	6120	54.9	6429	57.3	36 620	54.8	
≥40	366	1.7	394	2.3	256	2.3	305	2.7	1321	2.0	
**Maternal education (years)**											
<13	7480	34.4	6371	28.1	2923	26.2	2822	25.1	19 596	29.3	<0.001
13-16	9238	42.5	10 138	44.6	4954	44.5	4899	43.6	29 229	43.7	
>16	5030	23.1	6209	27.3	3266	29.3	3510	31.3	18 015	27.0	
**Parity**											<0.001
Primiparous	8733	40.2	10 546	46.4	5469	49.1	5677	50.6	30 425	45.5	
Multiparious	13 015	59.8	12 172	53.6	5674	50.9	5554	49.4	36 415	54.5	
**Pre-pregnancy BMI (kg/m²)**											<0.001
<18.5	534	2.5	685	3	336	3	358	3.2	1913	2.9	
18.5-24.9	13 320	61.2	15 087	66.4	7599	68.2	7974	71	43 980	65.8	
25-29.9	5385	24.8	4870	21.4	2273	20.4	2152	19.2	14 680	22	
≥30	2509	11.5	2076	9.1	935	8.4	747	6.7	6267	9.4	
**Maternal smoking in pregnancy**											<0.001
Never	17 759	90.7	19 278	92.7	9643	93.8	9707	93.8	56 387	92.4	
Ever	1826	9.3	1525	7.3	640	6.2	648	6.2	4628	7.6	
**Maternal vitamin D supplement use**	9134	42	20 446	90	11 031	99	11 175	99.5	51 667	77.3	<0.001
**Child´s gender**											0.198
Girls	10 711	49.3	11 122	49	5385	48.3	5412	48.2	32 630	48.8	
Boys	11 037	50.7	11 596	51	5758	51.7	5819	51.8	34 210	51.2	
	**Median**	**Range[b]**	**Median**	**Range[b]**	**Median**	**Range[b]**	**Median**	**Range[b]**	**Median**	**Range[b]**	
**Total maternal vitamin D intake (µg/day)**	3.3	1.1-4.8	7.1	5.2-9.7	12.0	10.2-14.6	19.8	15.4-33.6	7.2	1.8-23.0	<0.001
**Gestational age (weeks)**	40.1	37.3-42.1	40.1	37.3-42.1	40.3	37.3-42.1	40.3	37.3-42.1	40.1	37.3-42.1	0.224
**Gestational weight gain (kg)**	14.2	5-25	14.5	6-25	14.4	6-25	15.0	6-25	14.5	6-25	<0.001
**Birth weight (g)**	3630	2780-4480	3620	2780-4470	3620	2800-4430	3620	2780-4460	3620	2780-4464	<0.001
**Maternal energy intake (kcal)**	2092	1384-3166	2261	1491-3462	2321	1521-3558	2324	1527-3631	2222	1457-3436	<0.001

[a]Chi-2 test for categorical variables and ANOVA for continuous variables

For peer review only

[b] 5th to 95th percentile

For peer review only - http://bmjopen.bmj.com/site/about/guidelines.xhtml

Total maternal vitamin D intake during pregnancy and childhood growth

Weight and height growth trajectories and velocities from 1 month to 8 years

Overall, total maternal vitamin D intake was negatively associated with child weight growth from 1 month to 8 years for intakes ≥10 μg/day, compared to low intakes (<5 μg/day, reference group) (Table 2 and 3). Pre-pregnancy BMI modified the association between the exposure and the outcome (p-value for interaction <0.001). More specifically, among mothers with normal pre-pregnancy BMI, we found that higher maternal vitamin D intake was negatively associated with child's weight, especially during the first 3 months, compared to low intakes (Table 2 and 3). The negative association with child's weight persisted for intakes of 10-15 μg/day and for up to 2 years, while no further reduction in weight was observed for higher intakes and after 3 months (>15 μg/day). Similar negative associations with weight from 3 months to 2 years were found when we examined maternal vitamin D intake from supplements only (Supplementary Table 3 and 4). In addition, maternal vitamin D intakes of 10-15 μg/day were associated with a lower height trajectory, compared to lower intakes, while no other intake level was associated with the child's height. No association was found between maternal vitamin D intake and child´s weight or height growth velocity from 1 month to 8 years of age, in children of mothers with normal weight (Supplementary Table 8 and 9).

Among children of mothers with overweight pre-pregnancy BMI, any maternal vitamin D intake was positively associated with weight trajectory from 12 months onwards, compared to lower intakes (Table 2 and 3). Nevertheless, for intakes between 10-15 μg/day, the positive association with child´s weight growth was already observed from 6 months and onwards. Similar positive associations with child weight after 3 years were found when we examined the intake from food and supplements separately, but for different intake levels (Supplementary Table 3 and 4). For height growth trajectory, the only significant difference from the reference group was a positive association in children prenatally exposed to vitamin D intake of >15 μg/day at 7 and 8 years of age, but the effect estimate was small. No association was found between maternal vitamin D intake and weight or height growth velocity from 1 month to 8 years of age, in children of mothers with overweight (Supplementary Table 8 and 9).

The sensitivity analyses on the association between maternal vitamin D intake and reported anthropometrics, gave similar results as the main models, in both children of mothers with

For peer review only - http://bmjopen.bmj.com/site/about/guidelines.xhtml

normal weight and children of mothers with overweight (results not shown). Also, when excluding the highest 90th percentile of total maternal vitamin D intake, the associations seen in the main models on child´s weights and heights remained.

For peer review only

For peer review only - http://bmjopen.bmj.com/site/about/guidelines.xhtml

**Table 2.** Associations between total maternal vitamin D intake and childhood weight and height trajectories from 1 month to 2 years, using predicted anthropometrics.						
	**Infancy**			**Toddlerhood**		
	**1 month**	**3 months**	**6 months**	**12 months**	**18 months**	**2 years**
**Maternal vitamin D intake**	**Beta (95% CI)**	**Beta (95% CI)**	**Beta (95% CI)**	**Beta (95% CI)**	**Beta (95% CI)**	**Beta (95% CI)**
All mothers n=66 840						
**Weight (g)**						
<5 µg (ref)						
5-9.9 µg	-9.4 (-19.1, 0.2)	-9.2 (-19.9, 0.6)	-8.8 (-19.2, 1.5)	-8.1 (-20.7, 4.4)	-7.4 (-23.1, 8.3)	-6.7 (-26.0, 12.5)
10-15 µg	-7.6 (-19.5, 4.3)	-9.3 (-21.3, 2.8)	-11.7 (-24.5, 1.0)	-16.7 (-32.2, -1.2)	-21.6 (-40.9, -2.3)	-26.6 (-50.3, -2.9)
>15 µg	-14.7 (-26.7, -2.7)	-15.4 (-27.6, -3.3)	-16.6 (-29.5, -3.8)	-19.0 (-34.5, -3.5)	-21.4 (-40.7, -2.1)	-23.7 (-47.4, -0.1)
**Height (cm)**						
<5 µg (ref)						
5-9.9 µg	-0.01 (-0.07, 0.04)	-0.01 (-0.06, 0.04)	-0.01 (-0.06, 0.04)	-0.01 (-0.06, 0.04)	-0.01 (-0.05, 0.04)	-0.001 (-0.05, 0.04)
10-15 µg	-0.05 (-0.11, 0.01)	-0.05 (-0.11, 0.01)	-0.05 (-0.11, 0.01)	-0.05 (-0.11, 0.003)	-0.06 (-0.11, -0.002)	-0.06 (-0.11, -0.01)
>15 µg	-0.04 (-0.10, 0.03)	-0.04 (-0.10, 0.03)	-0.03 (-0.09, 0.03)	-0.03 (-0.09, 0.03)	-0.02 (-0.08, 0.03)	-0.02 (-0.07, 0.04)
Mothers with normal weight [a] ***n=41 970***						
**Weight (g)**						
<5 µg (ref)						
5-9.9 µg	-11.5 (-22.4, -0.6)	-10.9 (-22.0, 0.14)	-10.1 (-21.8, 1.6)	-8.4 (-22.5, 5.8)	-6.7 (-24.3, 10.9)	-5.0 (-26.6, 16.6)
10-15 µg	-14.5 (-27.7,-1.2)	-15.7 (-29.1, -2.3)	-17.5 (-31.7, -3.3)	-21.1 (-38.2, -4.0)	-24.7 (-46.0, -3.5)	-28.4 (-54.5, -2.3)
>15 µg	-15.2 (-28.3, -2.1)	-14.5 (-27.8, -1.2)	-13.5 (-27.5, 0.6)	-11.4 (-28.3, 5.5)	-9.3 (-30.4, 11.7)	-7.3 (-33.1, 18.5)
**Height (cm)**						
<5 µg (ref)						
5-9.9 µg	-0.02 (-0.08, 0.04)	-0.02 (-0.08, 0.04)	-0.02 (-0.07, 0.04)	-0.02 (-0.07, 0.04)	-0.01 (-0.06, 0.04)	-0.01 (-0.06, 0.04)
10-15 µg	-0.07 (-0.14, -0.003)	-0.07 (-0.14, -0.005)	-0.08 (-0.14, -0.01)	-0.08 (-0.15, -0.02)	-0.09 (-0.15, -0.03)	-0.09 (-0.15, -0.03)
>15 µg	-0.05 (-0.12, 0.02)	-0.05 (-0.12, 0.02)	-0.05 (-0.11, 0.02)	-0.04 (-0.11, 0.02)	-0.04 (-0.09, 0.02)	-0.03 (-0.09, 0.03)
Mothers with overweight [b] ***n=20 080***						
**Weight (g)**						
<5 µg (ref)						
5-9.9 µg	-5.5 (-19.8, 8.8)	-1.4 (-15.9, 13.1)	4.7 (-10.6, 20.0)	16.9 (-1.6, 35.4)	29.2 (6.1, 52.2)	41.4 (13.1, 69.7)
10-15 µg	10.3 (-8.0, 28.6)	14.4 (-4.1, 33.0)	20.6 (1.0, 40.2)	33.1 (9.3, 56.8)	45.5 (15.9, 75.0)	57.9 (21.7, 94.1)
>15 µg	-15.6 (-34.8, 3.7)	-9.9 (-29.5, 9.6)	-1.5 (-22.1, 19.1)	15.4 (-9.5, 40.3)	32.3 (1.2, 63.3)	49.2 (11.1, 87.3)

For peer review only - http://bmjopen.bmj.com/site/about/guidelines.xhtml

**Height (cm)**						
<5 µg (ref)						
5-9.9 µg	-0.02 (-0.09, 0.06)	-0.02 (-0.09, 0.06)	-0.01 (-0.08, 0.06)	-0.005 (-0.07, 0.06)	0.002 (-0.06, 0.07)	0.009 (-0.06, 0.07)
10-15 µg	-0.02 (-0.11, 0.08)	-0.02 (-0.11, 0.08)	-0.01 (-0.10, 0.08)	-0.003 (-0.09, 0.09)	0.005 (-0.08, 0.09)	0.01 (-0.07, 0.10)
>15 µg	-0.04 (-0.14, 0.07)	-0.03 (-0.13, 0.07)	-0.03 (-0.12, 0.07)	-0.01 (-0.11, 0.08)	-0.001 (-0.09, 0.09)	0.01 (-0.08, 0.10)

Beta=Beta coefficient, CI=Confidence interval

Effect estimates derive from multilevel mixed effects linear regression model, adjusted for maternal education, parity, maternal milk and yoghurt intake, maternal fiber intake, child´s gender and birth weight. The model with all mothers was also adjusted for maternal pre-pregnancy BMI.

[a] Mothers with pre-pregnancy BMI 18.5-24.9 kg/m^2

[b] Mothers with pre-pregnancy BMI ≥25 kg/m^2

For peer review only

Table 3. Associations between total maternal vitamin D intake and childhood weight and height trajectories from 3 years to 8 years, using predicted anthropometrics.

	**Preschool age**		**School age**
	**3 years**	**5 years**	**8 years**
**Maternal vitamin D intake**	**Beta (95% CI)**	**Beta (95% CI)**	**Beta (95% CI)**
All mothers n=66 840			
**Weight (g)**			
<5 µg (ref)			
5-9.9 µg	-5.3 (-32.4, 21.8)	-2.5 (-46.1, 41.2)	1.8 (-67.3, 70.9)
10-15 µg	-36.5 (-69.7, -3.2)	-56.3 (-110, -2.7)	-86.0 (-171, -1.1)
>15 µg	-28.5 (-61.7, 4.8)	-37.9 (-91.4, 15.5)	-52.1 (-137, 32.5)
**Height (cm)**			
<5 µg (ref)			
5-9.9 µg	0.01 (-0.04, 0.05)	0.02 (-0.03, 0.07)	0.04 (-0.03, 0.11)
10-15 µg	-0.06 (-0.11, -0.01)	-0.07 (-0.13, -0.01)	-0.09 (-0.17, 0.003)
>15 µg	-0.01 (-0.06, 0.05)	0.02 (-0.04, 0.08)	0.05 (-0.04, 0.14)

Mothers with normal weight [a] ***n=41 970***			
**Weight (g)**			
<5 µg (ref)			
5-9.9 µg	-1.6 (-31.9, 28.7)	5.2 (-43.6, 54.0)	15.3 (-62.0, 92.6)
10-15 µg	-35.6 (-72.3, 1.0)	-50.1 (-109, 8.9)	-71.8 (-165, 21.6)
>15 µg	-3.1 (-39.3, 33.0)	5.1 (-53.0, 63.3)	17.6 (-74.6, 110)
**Height (cm)**			
<5 µg (ref)			
5-9.9 µg	-0.004 (-0.05, 0.05)	0.008 (-0.05, 0.06)	0.03 (-0.06, 0.11)
10-15 µg	-0.1 (-0.16, -0.04)	-0.1 (-0.19, -0.05)	-0.1 (-0.25, -0.05)
>15 µg	-0.02 (-0.08, 0.04)	-0.003 (-0.07, 0.07)	0.03 (-0.07, 0.12)
Mothers with overweight [b] ***n=20 080***			
**Weight (g)**			
<5 µg (ref)			
5-9.9 µg	65.8 (26.1, 106)	115 (50.8, 179)	188 (86.8, 290)
10-15 µg	82.7 (31.9, 134)	132 (50.5, 214)	207 (77.2, 337)
>15 µg	82.9 (29.5, 136)	151 (64.4, 237)	252 (116, 388)
**Height (cm)**			
<5 µg (ref)			
5-9.9 µg	0.02 (-0.04, 0.09)	0.05 (-0.02, 0.13)	0.09, (-0.01, 0.20)
10-15 µg	0.03 (-0.05, 0.11)	0.06 (-0.03, 0.16)	0.1 (-0.02, 0.25)
>15 µg	0.04 (-0.05, 0.12)	0.09 (-0.01, 0.19)	0.2 (0.02, 0.31)

Beta=Beta coefficient, CI=Confidence interval

Effect estimates derive from multilevel mixed effects linear regression model, adjusted for maternal education, parity, maternal milk and yoghurt intake, maternal fiber intake, child´s gender and birth weight. The model with all mothers was also adjusted for maternal pre-pregnancy BMI.

[a] Mothers with pre-pregnancy BMI 18.5-24.9 kg/m^2

[b] Mothers with pre-pregnancy BMI ≥25 kg/m^2

For peer review only - http://bmjopen.bmj.com/site/about/guidelines.xhtml

Risk of rapid weight and height growth in infancy and toddlerhood

Overall, maternal vitamin D intakes of 5-9.9 and >15 µg/day was associated with lower odds of having a rapid weight gain at 12 months of age, both crude and adjusted, compared to low intakes (<5 µg/day) (Table 4).

When exploring the association by maternal pre-pregnancy BMI, children of mothers with normal pre-pregnancy weight, with total maternal vitamin D intakes of 5-9.9, 10-15 and >15 µg/day during pregnancy, had lower odds of having a rapid weight gain during the first year of life, both crude and adjusted compared to the reference group (Table 4). Similar lower OR were found for the risk of rapid height gain from birth to 12 months, but the confidence interval included 1. The associations with rapid weight and height gain from birth to 2 years were weaker.

For children of mothers with pre-pregnancy overweight, we found higher odds ratio for rapid weight and height gain at 12 months and 2 years, both crude and adjusted, but these associations were weak and the confidence intervals included 1 (Table 4).

In the sensitivity analyses, when excluding the 90th percentile of maternal vitamin D intakes, similar results were obtained as in the main models, for both children of mothers with normal weight, and for children of mothers with overweight.

Table 4. Associations (OR and 95% CI) between total maternal vitamin D intake and rapid growth from birth to 12 months and from birth to 2 years, using reported anthropometrics.

	**Weight growth from birth**		**Height growth from birth**	
**Maternal vitamin D intake**	**12 months**	**2 years**	**12 months**	**2 years**
All mothers	n=39 296, 59% [a]	n=18 360, 27%	n=31 640, 47%	n=14 946, 22%
	**OR (95% CI)**	**OR (95% CI)**	**OR (95% CI)**	**OR (95% CI)**
<5 µg (ref)				
5-9.9 µg	0.91 (0.85, 0.97)	1.03 (0.93, 1.15)	1.05 (0.97, 1.13)	1.01 (0.90, 1.14)
10-15 µg	0.93 (0.86, 1.02)	1.02 (0.90, 1.16)	1.02 (0.93, 1.12)	0.95 (0.83, 1.09)
>15 µg	0.90 (0.83, 0.98)	0.97 (0.85, 1.10)	1.00 (0.91, 1.10)	1.04 (0.90, 1.19)
	**12 months**	**2 years**	**12 months**	**2 years**
Mothers with normal weight [b]	n=25 982, 62%	n=12 043, 29%	n=20 658, 49%	n=9700, 23%
	**OR (95% CI)**	**OR (95% CI)**	**OR (95% CI)**	**OR (95% CI)**
<5 µg (ref)				
5-9.9 µg	0.88 (0.81, 0.96)	1.04 (0.91, 1.18)	1.01 (0.91, 1.11)	1.05 (0.91, 1.21)
10-15 µg	0.85 (0.76, 0.95)	1.03 (0.89, 1.21)	0.94 (0.83, 1.06)	0.98 (0.82, 1.17)
>15 µg	0.86 (0.77, 0.95)	0.97 (0.84, 1.13)	0.94 (0.84, 1.06)	1.01 (0.85, 1.20)
	**12 months**	**2 years**	**12 months**	**2 years**

For peer review only - http://bmjopen.bmj.com/site/about/guidelines.xhtml

Mothers with overweight [c]	n=12 240, 61%	N=10 118, 50%	N=5838, 29%	N=4851, 24%
	**OR (95% CI)**	**OR (95% CI)**	**OR (95% CI)**	**OR (95% CI)**
<5 µg (ref)				
5-9.9 µg	0.96 (0.86, 1.08)	1.08 (0.90, 1.29)	1.12 (0.99, 1.28)	1.00 (0.82, 1.21)
10-15 µg	1.10 (0.95, 1.27)	1.01 (0.81, 1.27)	1.14 (0.97, 1.34)	0.87 (0.68, 1.12)
>15 µg	1.01 (0.87, 1.18)	0.91 (0.72, 1.16)	1.08 (0.91, 1.28)	1.08 (0.84, 1.38)

OR=Odds Ratio, CI=Confidence interval

Effect estimates derived by logistic regression, adjusted for maternal education, parity, maternal milk and yoghurt intake, maternal fiber intake and child´s gender. The model with all mothers was also adjusted for maternal pre-pregnancy BMI.

[a] Proportions of the eligible pregnancies with the reported anthropometrics available from birth to the given age.

[b] Mothers with pre-pregnancy BMI 18.5-24.9 kg/m^2

[c] Mothers with pre-pregnancy BMI ≥25 kg/m^2

For peer review only

Risk of childhood overweight in pre-school and school age

Overall, the prevalence of childhood overweight was 8.9% at 3 years, 18.2% at 5 years and 6.3% at 8 years of age. Maternal vitamin D intakes of 5-9.9 and >15 µg/day were associated with a higher risk of overweight at 8 years of age, compared to the reference group (Table 5). However, in children of mothers with normal weight, with total vitamin D intakes of 10-15 and >15 µg/day during pregnancy, 0.86 (95% CI 0.77, 0.97) and 0.88 (95% CI 0.79, 0.99) lower odds for overweight at 3 years of age were found, both crude and adjusted, compared to the reference group (Table 5). At 5 years, children of mothers with vitamin D intakes of 10-15 µg/day during pregnancy, had 0.91 (95% CI 0.84, 0.99) lower adjusted odds of being overweight, compared to the reference group. At 8 years there were no association found for any intake group. For children of mothers with overweight, we found an increased risk of overweight at 5 years (OR=1.09, 95% CI 1.01, 1.18) and at 8 years (OR=1.12, 95% CI 1.02, 1.23) for children of mothers with vitamin D intakes of 5-9.9 µg/day, compared to the reference group (Table 5). However, no association between maternal vitamin D intake and risk of overweight was found for children of mothers with intakes of 10-15 or >15 µg/day at 3 years, 5 years or 8 years of age.

In the sensitivity analyses on childhood overweight using the reported weights and heights, the results for the children of mothers with normal weight, with vitamin D intake >15 µg/day persisted (OR=0.87, 95% CI 0.77, 0.98) (Supplementary Table 10). Also, for children of mothers with overweight, with total vitamin D intake of 5-9.9 µg/day, a significant odds ratio (OR=1.22, 95% CI 1.06, 1.40) was obtained. After exclusion of the highest 90th percentile of maternal vitamin D intakes, similar results as in the main models were obtained, both in children of mothers with normal weight and with overweight. Adjusting for birth weight did

For peer review only - http://bmjopen.bmj.com/site/about/guidelines.xhtml

not change the direction of the associations, neither in children of mothers with normal weight, nor in children of mothers with overweight.

Table 5. Associations (OR and 95% CI) between total maternal vitamin D intake and risk of childhood overweight at 3, 5 and 8 years, using predicted anthropometrics.

	**3 years**	**5 years**	**8 years**
**Maternal vitamin D intake**	**OR (95% CI)**	**OR (95% CI)**	**OR (95% CI)**
All mothers ***n=66 840***			
<5 µg (ref)			
5-9.9 µg	0.99 (0.93, 1.06)	1.03 (0.98, 1.08)	1.10 (1.03, 1.17)
10-15 µg	0.93 (0.86, 1.01)	0.97 (0.91, 1.04)	1.02 (0.94, 1.11)
>15 µg	0.97 (0.89, 1.06)	1.00 (0.94, 1.06)	1.09 (1.01, 1.19)
p-value for trend [a]	0.222	0.553	0.098
Mothers with normal weight [b] ***n=41 970***			
<5 µg (ref)			
5-9.9 µg	0.92 (0.84, 1.01)	0.97 (0.91, 1.04)	1.07 (0.98, 1.17)
10-15 µg	0.86 (0.77, 0.97)	0.91 (0.84, 0.99)	0.98 (0.87, 1.09)
>15 µg	0.88 (0.79, 0.99)	0.94 (0.87, 1.02)	1.05 (0.94, 1.17)
p-value for trend [a]	0.015	0.039	0.920
Mothers with overweight [c] ***n=20 080***			
<5 µg (ref)			
5-9.9 µg	1.07 (0.97, 1.18)	1.09 (1.01, 1.18)	1.12 (1.02, 1.23)
10-15 µg	1.01 (0.89, 1.14)	1.05 (0.95, 1.16)	1.06 (0.94, 1.20)
>15 µg	1.06 (0.93, 1.20)	1.03 (0.93, 1.14)	1.11 (0.98, 1.26)
p-value for trend [a]	0.513	0.443	0.156

OR=Odds Ratio, CI=Confidence interval

Effect estimates derived by logistic regression, adjusted for maternal education, parity, maternal milk and yoghurt intake, maternal fiber intake and child´s gender. The model with all mothers was also adjusted for maternal pre-pregnancy BMI.

[a] P-value for linear trend, obtained by regression with maternal vitamin D intake in categories as continuous variable.

[b] Mothers with pre-pregnancy BMI 18.5-24.9 kg/m^2

[c] Mothers with pre-pregnancy BMI ≥25 kg/m^2

Siblings with discordant total maternal vitamin D intake

When comparing weight and height growth trajectories of discordant siblings, the analyzes showed similar estimates as the main models, but non-significant differences (Supplementary Table 5 and 6). The growth trajectories did not differ between siblings with discordant maternal vitamin D intake. When comparing the risk of childhood overweight at 3 years, 5 years and 8 years in discordant siblings, the same effect estimates as for the main models were obtained, however not significant (Supplementary Table 7).

Discussion

In this longitudinal study of the associations between maternal vitamin D intake and child growth, we identified pre-pregnancy BMI as a major modifying factor. Among mothers with normal pre-pregnancy BMI, a maternal vitamin D intake ≥10 µg/day was associated with

For peer review only - http://bmjopen.bmj.com/site/about/guidelines.xhtml

lower weight and height growth trajectories starting in infancy and persisting in toddlerhood. Higher maternal vitamin D intake was also associated with lower odds of a rapid weight gain during the first year of life and of child overweight in pre-school years. The results indicated associations in opposing directions in children of mothers with pre-pregnancy overweight. The discordant sibling analysis revealed similar but weaker associations, possibly due to small sample size, and supports the hypothesis. Our findings suggest that in children of normal weight mothers, vitamin D intake during pregnancy has a protective effect against rapid growth and overweight, perhaps due to a more favorable weight growth trajectory in infancy.

The main strengths of this study are the longitudinal design and the large, nationwide [33] study sample. The FFQ, used to capture the dietary vitamin D intake, was customized for assessing diet in the first half of pregnancy. Validation studies on the FFQ concluded the questionnaire to provide adequate estimates of vitamin D intakes from foods and supplements [39, 40]. Another strength was the use of a growth curve model, which is an approach to compensate for the loss to follow-up, by predicting weights and heights for all children. The similar estimates found in the sensitivity analyses of the reported weights and heights of the children bring some validity in the predicted anthropometrics of growth trajectories and childhood overweight. In addition, the drawing of a directed acyclic graph reduced the risk of confounding bias, since the models were adjusted for relevant covariates. Our study also has several weaknesses. Firstly, maternal anthropometrics, child anthropometrics and food intake measures are self-reported and prone to misreport [39, 52, 53]. Although the women were not aware of the outcome of interest, maternal weight status and other characteristic are likely to influence the accuracy of self-reported information. It is known that overweight and obese women misreport their bodyweight and food intake to a larger degree than normal weight women, pertaining also to pregnant women [52]. Although self-reported weight and height encompass inaccuracy, self-report is a cost-effective alternative in large cohorts and the inaccuracy is not likely to largely bias the results [54]. It is also possible that the association we found could be explained by another food, nutrient or behavior related to the maternal vitamin D intake that was not taken into account, or residual confounding.

The protective effect of maternal vitamin D in pregnancy on the risk of overweight in children from mothers with pre-pregnancy normal weight is consistent with previous findings from a Dutch [23] and a Spanish study [26]. However, previous studies investigating the association between maternal vitamin D status and childhood growth or overweight, have

For peer review only - http://bmjopen.bmj.com/site/about/guidelines.xhtml

explored the association by adjusting for pre-pregnancy BMI. We are the first study to report an interaction of pre-pregnancy maternal BMI when investigating the relation between maternal vitamin D intake with child growth and overweight. To simply adjust for pre-pregnancy BMI could potentially fail to capture the variation in maternal body size and thereby the magnitude of the distribution and dilution of vitamin D in the body [5]. This may explain the somewhat opposing results found in previous studies on the association on maternal vitamin D status and child´s growth, anthropometrics and risk of overweight [23, 24, 26-30].

Several possible pathways behind the association between maternal vitamin D intake, growth and risk of overweight in childhood have been suggested. Some studies have for example found associations between vitamin D deficiency and increased maternal insulin resistance in pregnancy and gestational diabetes mellitus [55-57]. It is suggested that if the fetus is exposed to hyperglycemia in utero, the fetal insulin production may be altered and the risk of type 2 diabetes later in life increased [58]. Also, children born by mothers affected by gestational diabetes mellitus are more likely to be overweight [59]. If low vitamin D intake in pregnancy increases maternal insulin resistance, this may be one mechanism programming the fetus towards increased susceptibility of metabolic diseases including overweight in childhood.

Our results underscore the importance of adequate vitamin D intake for the pregnant woman on growth in childhood. Moreover, our results highlight the significant contribution of supplements to reach the recommended vitamin D intake in a population with relatively low intake from foods. These findings suggest that maternal vitamin D intake is a potential modifiable factor for prevention of rapid postnatal growth in children of normal weight mothers. Since there is a lack of previous studies investigating the association between maternal vitamin D intake and childhood growth and overweight, further studies will need to replicate our results before the relevance can be fully determined.

Conclusion

Our findings supports that vitamin D intake during pregnancy affects offspring postnatal growth, and that a higher vitamin D intake during pregnancy has a protective effect against the risk of childhood overweight but only in children of mothers with normal pre-pregnancy

For peer review only - http://bmjopen.bmj.com/site/about/guidelines.xhtml

weight. In pregnancy, achieving the recommended vitamin D intake may reduce the risks of overweight in children of mothers with normal weight.

Acknowledgements

The Norwegian Mother, Father and Child Cohort Study is supported by the Norwegian Ministry of Health and Care Services and the Ministry of Education and Research. The authors thank the MoBa cohort funders and all the participating families in Norway for their valuable contribution.

Author contributions

HA initiated the study, all authors contributed with planning the study, ALB is responsible for data protection and access, AA conducted the statistical analyses and wrote the first version of the manuscript, EP assisted with the statistical analyses. All authors (HA, AA, EP, ALB, LL, AW and HMM) were involved in interpretation of the results and writing of the final manuscript.

Competing interests

None declared.

Patient consent

Obtained.

Funding statement

The current study was funded by the Swedish Research Council for Health, Working Life and Welfare (grant number 2018-00441) and the Sahlgrenska Academy (U2018/162). EP was funded by the Research Council of Norway, under the MILJØFORSK program (project number 268465).

Data sharing statement

No additional data are available. All data from the MoBa study are available to all qualified researchers/research groups in Norway and to international researchers who are collaborating with a Norwegian researcher.

For peer review only - http://bmjopen.bmj.com/site/about/guidelines.xhtml

References

1. Theodoratou, E., et al., *Vitamin D and multiple health outcomes: umbrella review of systematic reviews and meta-analyses of observational studies and randomised trials.* Bmj, 2014. **348**: p. g2035.
2. Hossein-nezhad, A. and M.F. Holick, *Optimize dietary intake of vitamin D: an epigenetic perspective.* Curr Opin Clin Nutr Metab Care, 2012. **15**(6): p. 567-79.
3. O'Neill, C.M., et al., *Seasonal Changes in Vitamin D-Effective UVB Availability in Europe and Associations with Population Serum 25-Hydroxyvitamin D.* Nutrients, 2016. **8**(9).
4. Hossein-nezhad, A. and M.F. Holick, *Vitamin D for health: a global perspective.* Mayo Clin Proc, 2013. **88**(7): p. 720-55.
5. Walsh, J.S., S. Bowles, and A.L. Evans, *Vitamin D in obesity.* Curr Opin Endocrinol Diabetes Obes, 2017. **24**(6): p. 389-394.
6. Saraf, R., et al., *Global summary of maternal and newborn vitamin D status - a systematic review.* Matern Child Nutr, 2016. **12**(4): p. 647-68.
7. Eggemoen, Å.R., et al., *Vitamin D levels during pregnancy and associations with birth weight and body composition of the newborn: a longitudinal multiethnic population-based study.* The British journal of nutrition, 2017. **117**(7): p. 985-993.
8. Barebring, L., et al., *Vitamin D Status during Pregnancy in a Multi-Ethnic Population-Representative Swedish Cohort.* Nutrients, 2016. **8**(10).
9. Itkonen, S.T., et al., *Vitamin D status and current policies to achieve adequate vitamin D intake in the Nordic countries.* Scand J Public Health, 2020: p. 1403494819896878.
10. Gluckman, P.D., M.A. Hanson, and T. Buklijas, *A conceptual framework for the developmental origins of health and disease.* J Dev Orig Health Dis, 2010. **1**(1): p. 6-18.
11. Kwon, E.J. and Y.J. Kim, *What is fetal programming?: a lifetime health is under the control of in utero health.* Obstet Gynecol Sci, 2017. **60**(6): p. 506-519.
12. Robinson, S.M., et al., *Modifiable early-life risk factors for childhood adiposity and overweight: an analysis of their combined impact and potential for prevention.* Am J Clin Nutr, 2015. **101**(2): p. 368-75.
13. Ong, K.K. and R.J. Loos, *Rapid infancy weight gain and subsequent obesity: systematic reviews and hopeful suggestions.* Acta Paediatr, 2006. **95**(8): p. 904-8.
14. Woo Baidal, J.A., et al., *Risk Factors for Childhood Obesity in the First 1,000 Days: A Systematic Review.* Am J Prev Med, 2016. **50**(6): p. 761-779.
15. Karaolis-Danckert, N., et al., *Rapid growth among term children whose birth weight was appropriate for gestational age has a longer lasting effect on body fat percentage than on body mass index.* Am J Clin Nutr, 2006. **84**(6): p. 1449-55.
16. WHO, *Commission on Ending Childhood Obesity. Report of the Commission on Ending Childhood Obesity*. 2016.
17. *UNICEF, WHO, World Bank. Global and regional trends by UN Regions, 1990-2030. Overweight: 1990-2018* 2015 [cited 2020.
18. Shin, J.S., et al., *Vitamin D effects on pregnancy and the placenta.* Placenta, 2010. **31**(12): p. 1027-34.
19. von Websky, K., et al., *Impact of vitamin D on pregnancy-related disorders and on offspring outcome.* J Steroid Biochem Mol Biol, 2018. **180**: p. 51-64.
20. Holick, M.F. and T.C. Chen, *Vitamin D deficiency: a worldwide problem with health consequences.* Am J Clin Nutr, 2008. **87**(4): p. 1080s-6s.
21. Tao, R.-X., et al., *Current Recommended Vitamin D Prenatal Supplementation and Fetal Growth: Results From the China-Anhui Birth Cohort Study.* The Journal of clinical endocrinology and metabolism, 2018. **103**(1): p. 244-252.

For peer review only - http://bmjopen.bmj.com/site/about/guidelines.xhtml

22. Aghajafari, F., et al., *Association between maternal serum 25-hydroxyvitamin D level and pregnancy and neonatal outcomes: systematic review and meta-analysis of observational studies.* Bmj, 2013. **346**: p. f1169.
23. Leffelaar, E.R., T.G.M. Vrijkotte, and M. van Eijsden, *Maternal early pregnancy vitamin D status in relation to fetal and neonatal growth: results of the multi-ethnic Amsterdam Born Children and their Development cohort.* The British journal of nutrition, 2010. **104**(1): p. 108-117.
24. Crozier, S.R., et al., *Maternal vitamin D status in pregnancy is associated with adiposity in the offspring: findings from the Southampton Women's Survey.* The American journal of clinical nutrition, 2012. **96**(1): p. 57-63.
25. Krishnaveni, G.V., et al., *Maternal vitamin D status during pregnancy and body composition and cardiovascular risk markers in Indian children: the Mysore Parthenon Study.* The American journal of clinical nutrition, 2011. **93**(3): p. 628-635.
26. Morales, E., et al., *Deficit of vitamin D in pregnancy and growth and overweight in the offspring.* International journal of obesity (2005), 2015. **39**(1): p. 61-68.
27. Daraki, V., et al., *Low maternal vitamin D status in pregnancy increases the risk of childhood obesity.* Pediatric obesity, 2018. **13**(8): p. 467-475.
28. Eckhardt, C.L., et al., *Maternal vitamin D status and infant anthropometry in a US multi-centre cohort study.* Annals of human biology, 2015. **42**(3): p. 215-222.
29. Ong, Y.L., et al., *The association of maternal vitamin D status with infant birth outcomes, postnatal growth and adiposity in the first 2 years of life in a multi-ethnic Asian population: the Growing Up in Singapore Towards healthy Outcomes (GUSTO) cohort study.* The British journal of nutrition, 2016. **116**(4): p. 621-631.
30. Gale, C.R., et al., *Maternal vitamin D status during pregnancy and child outcomes.* European journal of clinical nutrition, 2008. **62**(1): p. 68-77.
31. Horan, M.K., et al., *The association between maternal dietary micronutrient intake and neonatal anthropometry - secondary analysis from the ROLO study.* Nutrition journal, 2015. **14**: p. 105-105.
32. Scholl, T.O. and X. Chen, *Vitamin D intake during pregnancy: association with maternal characteristics and infant birth weight.* Early human development, 2009. **85**(4): p. 231-234.
33. Magnus, P., et al., *Cohort Profile Update: The Norwegian Mother and Child Cohort Study (MoBa).* Int J Epidemiol, 2016. **45**(2): p. 382-8.
34. Magnus, P., et al., *Cohort profile: the Norwegian Mother and Child Cohort Study (MoBa).* Int J Epidemiol, 2006. **35**(5): p. 1146-50.
35. *Questionnaires from MoBa*. [cited 2021- 08-04]; Available from: https://www.fhi.no/en/studies/moba/for-forskere-artikler/questionnaires-from-moba/.
36. Irgens, L.M., *The Medical Birth Registry of Norway. Epidemiological research and surveillance throughout 30 years.* Acta Obstet Gynecol Scand, 2000. **79**(6): p. 435-9.
37. Meltzer, H.M., et al., *Methodological challenges when monitoring the diet of pregnant women in a large study: experiences from the Norwegian Mother and Child Cohort Study (MoBa).* Matern Child Nutr, 2008. **4**(1): p. 14-27.
38. *What is the Norwegian Mother, Father and Child Cohort Study?* [cited 2021-08-18; Available from: https://www.fhi.no/en/studies/moba/what-is-the-norwegian-mother-and-child-cohort-study/.
39. Brantsaeter, A.L., et al., *Validity of a new food frequency questionnaire for pregnant women in the Norwegian Mother and Child Cohort Study (MoBa).* Matern Child Nutr, 2008. **4**(1): p. 28-43.
40. Brantsaeter, A.L., et al., *Self-reported dietary supplement use is confirmed by biological markers in the Norwegian Mother and Child Cohort Study (MoBa).* Ann Nutr Metab, 2007. **51**(2): p. 146-54.

For peer review only - http://bmjopen.bmj.com/site/about/guidelines.xhtml

41. Nordic Council of Ministers, N.C.o.M.S., *Nordic Nutrition Recommendations 2012: Integrating nutrition and physical activity* 2014.
42. Jenss RM, B.N., *A mathematical method for studying the growth of a child. .* Human Biology, 1937. **9**: p. 556-563.
43. Chirwa, E.D., et al., *Multi-level modelling of longitudinal child growth data from the Birth-to-Twenty Cohort: a comparison of growth models.* Annals of human biology, 2014. **41**(2): p. 168-179.
44. Keijzer-Veen, M.G., et al., *A regression model with unexplained residuals was preferred in the analysis of the fetal origins of adult diseases hypothesis.* J Clin Epidemiol, 2005. **58**(12): p. 1320-4.
45. Cole, T.J. and T. Lobstein, *Extended international (IOTF) body mass index cut-offs for thinness, overweight and obesity.* Pediatr Obes, 2012. **7**(4): p. 284-94.
46. Patro Golab, B., et al., *Influence of maternal obesity on the association between common pregnancy complications and risk of childhood obesity: an individual participant data meta-analysis.* Lancet Child Adolesc Health, 2018. **2**(11): p. 812-821.
47. Drincic, A.T., et al., *Volumetric dilution, rather than sequestration best explains the low vitamin D status of obesity.* Obesity (Silver Spring), 2012. **20**(7): p. 1444-8.
48. Rito, A.I., et al., *Association between Characteristics at Birth, Breastfeeding and Obesity in 22 Countries: The WHO European Childhood Obesity Surveillance Initiative - COSI 2015/2017.* Obes Facts, 2019. **12**(2): p. 226-243.
49. WHO, *Obesity: Preventing and managing the global epidemic. Report of a WHO consultation on obesity. p. 1–253*. 2000.
50. Petersen, A.H. and T. Lange, *What Is the Causal Interpretation of Sibling Comparison Designs?* Epidemiology, 2020. **31**(1): p. 75-81.
51. Susser, E., M.G. Eide, and M. Begg, *Invited commentary: The use of sibship studies to detect familial confounding.* Am J Epidemiol, 2010. **172**(5): p. 537-9.
52. Engstrom, J.L., et al., *Accuracy of self-reported height and weight in women: an integrative review of the literature.* J Midwifery Womens Health, 2003. **48**(5): p. 338-45.
53. Olafsdottir, A.S., et al., *Comparison of women's diet assessed by FFQs and 24-hour recalls with and without underreporters: associations with biomarkers.* Ann Nutr Metab, 2006. **50**(5): p. 450-60.
54. Headen, I., et al., *The accuracy of self-reported pregnancy-related weight: a systematic review.* Obes Rev, 2017. **18**(3): p. 350-369.
55. Chen, X., et al., *Sex Dependent Association of Vitamin D with Insulin Resistance.* J Clin Endocrinol Metab, 2021.
56. Zaki, M., et al., *Association of vitamin D receptor gene polymorphism (VDR) with vitamin D deficiency, metabolic and inflammatory markers in Egyptian obese women.* Genes Dis, 2017. **4**(3): p. 176-182.
57. Senti, J., D.K. Thiele, and C.M. Anderson, *Maternal vitamin D status as a critical determinant in gestational diabetes.* J Obstet Gynecol Neonatal Nurs, 2012. **41**(3): p. 328-38.
58. Tian, M., et al., *Low birth weight, a risk factor for diseases in later life, is a surrogate of insulin resistance at birth.* J Hypertens, 2019. **37**(11): p. 2123-2134.
59. Gillman, M.W., et al., *Maternal gestational diabetes, birth weight, and adolescent obesity.* Pediatrics, 2003. **111**(3): p. e221-6.

For peer review only - http://bmjopen.bmj.com/site/about/guidelines.xhtml

Supplementary table 1. Study population retention and anthropometric measures of the children at the 11 ages.

Intended age	Reported age	Weight (kg)		Response rate	Height (cm)		Response rate
	Mean (SD)	N	Mean (SD)	%	N	Mean (SD)	%
1.5 months	1.4 (0.2) months	56 625	5.0 (0.7)	84	45 745	57 (2.3)	67
3 months	3.1 (0.3) months	62 237	6.4 (0.8)	92	61 266	62 (2.5)	91
6 months	5.8 (0.5) months	60 515	7.9 (1.0)	89	60 224	68 (2.5)	89
8 months	8.0 (0.8) months	49 528	8.8 (1.0)	73	49 354	71 (2.7)	73
12 months	12.2 (0.6) months	49 609	9.9 (1.1)	73	49 686	76 (2.7)	73
15-18 months	16.0 (1.3) months	50 393	10.9 (1.2)	75	50 168	81 (3.0)	74
2 years	2.1 (0.2) years	25 195	13.0 (1.5)	37	25 346	89 (3.7)	37
3 years	3.0 (0.1) years	36 987	15.1 (1.8)	55	36 365	97 (3.8)	54
5 years	5.2 (0.3) years	30 288	20.0 (2.8)	45	31 671	113 (5.2)	47
7 years	7.1 (0.1) years	36 480	25.1 (4.0)	54	38 130	126 (5.4)	56
8 years	8.1 (0.1) years	23 123	28.2 (4.5)	34	24 611	132 (5.7)	36

For peer review only

Supplementary table 2. Cut-offs of overweight in children

Age (years)	Overweight cut-offs[a, b]		Prevalence (%)[c]	
	Boys	Girls	Boys	Girls
**3**	17.85	17.64	9.9	9.5
**5**	17.39	17.23	18.8	20.5
**8**	18.41	18.28	5.5	8.4

[a] Cole TJ, Lobstein T. Extended international (IOTF) body mass index cut-offs for thinness, overweight and obesity. Pediatr Obes. 2012;7(4):284-294. doi:10.1111/j.2047-6310.2012.00064.x

[b] All children with a body mass index equal to, or above, the cut-off are denoted as overweight

[c] Based on child´s body mass index calculated from the predicted anthropometric data.

For peer review only

Supplementary table 3. Associations between maternal vitamin D intake from foods alone, and from supplements alone, and predicted weight growth trajectories from 1 month to 2 years of age.

	Infancy			Toddlerhood		
	**1 month**	**3 months**	**6 months**	**12 months**	**18 months**	**2 years**
	**Beta (95% CI)**	**Beta (95% CI)**	**Beta (95% CI)**	**Beta (95% CI)**	**Beta (95% CI)**	**Beta (95% CI)**
**Maternal vitamin D intake from foods alone**						
All mothers n=66 840						
<2.5 µg						
2.5-4.9 µg	-5.8 (-14.6, 2.99)	-6.2 (-15.1, 2.7)	-6.7 (-16.1, 2.6)	-7.9 (-19.2, 3.5)	-9.0 (-23.1, 5.1)	-10.2 (-27.4, 7.1)
5-7.5 µg	-9.7 (-22.8, 3.5)	-7.1 (-20.4, 6.2)	-3.3 (-17.4, 10.7)	4.3 (-12.5, 21.1)	11.9 (-8.9, 32.7)	19.5 (-5.9, 45.0)
>7.5 µg	-12.0 (-34.8, 10.8)	-11.6 (-34.7, 11.4)	-11.0 (-35.3, 13.3)	-9.8 (-39.0, 19.3)	-8.7 (-44.7, 27.4)	-7.5 (-51.6, 36.6)
Mothers with normal weight [a] n=41 970						
<2.5 µg						
2.5-4.9 µg	-6.4 (-16.2, 3.4)	-7.0 (-16.9, 3.0)	-7.8 (-18.3, 2.7)	-9.3 (-22.0, 3.4)	-10.9 (-26.7, 4.9)	-12.5 (-31.9, 6.9)
5-7.5 µg	-7.3 (-21.7, 7.1)	-5.4 (-19.9, 9.2)	-2.5 (-17.8, 12.9)	3.3 (-15.1, 21.8)	9.1 (-13.8, 32.0)	14.9 (-13.1, 42.9)
>7.5 µg	-14.9 (-40.1, 10.3)	-14.8 (-40.3, 10.7)	-14.6 (-41.5, 12.3)	-14.1 (-46.5, 18.2)	-13.7 (-53.8, 26.4)	-13.2 (-62.3, 35.8)
Mothers with overweigh [b] n=20 080						
<2.5 µg						
2.5-4.9 µg	-4.3 (-17.5, 9.0)	-4.3 (-17.7, 9.2)	-4.3 (-18.5, 9.9)	-4.4 (-21.6, 12.8)	-4.5 (-25.9, 16.9)	-4.6 (-30.9, 21.7)
5-7.5 µg	-15.3 (-35.2, 4.6)	-11.3 (-31.4, 8.8)	-5.3 (-26.6, 15.9)	6.6 (-19.0, 32.3)	18.6 (-13.2, 50.4)	30.6 (-8.4, 69.6)
>7.5 µg	-5.1 (-39.1, 28.9)	-4.1 (-38.5, 30.3)	-2.6 (-38.9, 33.7)	0.3 (-43.5, 44.1)	3.3 (-51.1, 57.7)	6.2 (-60.4, 72.8)
**Maternal vitamin D intake from supplements alone**						
All mothers n=66 840						
<5 µg						
5-9.9 µg	-11.8 (-21.6, -2.1)	-11.4 (-21.3, -1.5)	-10.8 (-21.2, -0.3)	-9.5 (-22.1, 3.2)	-8.2 (-23.9, 7.6)	-6.9 (-26.2, 12.4)
10-15 µg	-4.0 (-17.4, 9.3)	-4.1 (-17.6, 9.4)	-4.1 (-18.4, 10.2)	-4.1 (-21.4, 13.2)	-4.2 (-25.7, 17.3)	-4.2 (-30.7, 22.2)
>15 µg	-8.5 (-21.5, 4.5)	-7.0 (-20.1, 6.2)	-4.6 (-18.5, 9.3)	0.1 (-16.8, 17.9)	4.8 (-16.2, 25.8)	9.5 (-16.2, 35.3)
Mothers with normal weight [a] n=41 970						
<5 µg						

5-9.9 µg	-10.6 (-21.4, 0.2)	-11.7 (-22.6, -0.7)	-13.3 (-24.9, -1.8)	-16.6 (-30.6, -2.6)	-19.9 (-37.4, -2.5)	-23.2 (-44.6, -1.8)
10-15 µg	-5.8 (-20.4, 8.7)	-7.1 (-21.9, 7.6)	-9.1 (-24.7, 6.5)	-13.0 (-31.9, 5.9)	-16.9 (-40.5, 6.6)	-20.9 (-49.7, 8.0)
>15 µg	-8.6 (-22.5, 5.4)	-8.2 (-22.4, 5.9)	-7.7 (-22.6, 7.3)	-6.6 (-24.7, 11.5)	-5.6 (-28.1, 17.0)	-4.5 (-32.2, 23.2)
Mothers with overweight [b] ***n=20 080***						
<5 µg						
5-9.9 µg	-14.9 (-29.8, 0.1)	-10.8 (-25.9, 4.3)	-4.7 (-20.7, 11.3)	7.5 (-11.8, 26.9)	20.0 (-4.4, 43.9)	32.0 (2.3, 61.6)
10-15 µg	0.2 (-21.6, 22.0)	3.2 (-18.8, 25.3)	7.8 (-15.5, 31.2)	17.0 (-11.3, 45.3)	26.1 (-9.2, 61.4)	35.2 (-8.1, 78.6)
>15 µg	-8.4 (-30.2, 13.4)	-4.0 (-26.0, 18.1)	2.7 (-20.6, 26.0)	16.1 (-12.2, 44.3)	29.4 (-5.8, 64.7)	42.8 (-0.4, 86.1)

Beta=Beta coefficient, CI=Confidence interval

Effect estimates derive from multilevel mixed effects linear regression model, adjusted for maternal education, parity, maternal milk and yoghurt intake, maternal fiber intake, child´s gender and birth weight. The model with all mothers was also adjusted for maternal pre-pregnancy BMI.

[a] Mothers with pre-pregnancy BMI 18.5-24.9 kg/m^2

[b] Mothers with pre-pregnancy BMI ≥25 kg/m^2

For peer review only

Supplementary table 4. Associations between maternal vitamin D intake from foods alone, and from supplements alone, and predicted weight growth trajectories from 3 years to 8 years of age.

	**Preschool age**		**School age**
	**3 years**	**5 years**	**8 years**
	**Beta (95% CI)**	**Beta (95% CI)**	**Beta (95% CI)**
**Maternal vitamin D intake from foods alone**			
All mothers ***n=66 840***			
<5 µg			
5-9.9 µg	-12.4 (-36.7, 11.8)	-17.0 (-56.0, 22.0)	-23.8 (-85.6, 38.0)
10-15 µg	34.8 (-0.8, 70.3)	65.2 (8.1, 122)	111 (20.5, 201)
>15 µg	-5.1 (-66.8, 56.5)	-0.4 (-99.4, 98.6)	6.7 (-150, 163)
Mothers with normal weight [a] ***n=41 970***			
<5 µg			
5-9.9 µg	-15.7 (-42.8, 11.5)	-22.0 (-65.8, 21.8)	-31.4 (-100, 37.9)
10-15 µg	26.4 (-12.7, 65.6)	49.6 (-13.4, 113)	84.3 (-15.3, 184)
>15 µg	-12.4 (-81.0, 56.3)	-10.6 (-121, 99.8)	-8.0 (-183, 167)
Mothers with overweight [b] ***n=20 080***			
<5 µg			
5-9.9 µg	-4.8 (-41.7, 32.1)	-5.1 (-64.6, 54.2)	-5.7 (-99.8, 88.3)
10-15 µg	54.5 (-0.1, 109)	102.4 (14.5, 190)	174.3 (35.1, 313)
>15 µg	12.1 (-81.3, 106)	23.9 (-126, 174)	41.7 (-196, 280)
**Maternal vitamin D intake from supplements alone**			
All mothers ***n=66 840***			
<5 µg			
5-9.9 µg	-4.3 (-31.4, 22.8)	0.9 (-42.7, 44.6)	8.7 (-60.5, 77.9)
10-15 µg	-4.3 (-41.4, 32.8)	-4.5 (-64.3, 55.2)	-4.8 (-99.5, 89.8)
>15 µg	18.9 (-17.2, 55.1)	37.8 (-20.5, 96.0)	66.0 (-26.3, 158)
Mothers with normal weight [a] ***n=41 970***			
<5 µg			

5-9.9 µg	-29.8 (-59.9, 0.3)	-43.0 (-91.5, 5.4)	-62.8 (-140, 13.9)
10-15 µg	-28.7 (-69.3, 11.9)	-44.4 (-110, 21.0)	-67.9 (-171, 35.6)
>15 µg	-2.4 (-41.2, 36.5)	1.9 (-60.7, 64.5)	8.2 (-90.9, 107)
Mothers with overweight [b] ***n=20 080***			
<5 µg			
5-9.9 µg	56.4 (14.8, 98.0)	105 (38.2, 172)	179 (72.3, 285)
10-15 µg	53.5 (-7.3, 114)	90.1 (-7.9, 188)	145 (-10.3, 300)
>15 µg	69.5 (8.8, 130)	123 (25.1, 221)	203 (48.2, 358)

Beta=Beta coefficient, CI=Confidence interval

Effect estimates derive from multilevel mixed effects linear regression model, adjusted for maternal education, parity, maternal milk and yoghurt intake, maternal fiber intake, child´s gender and birth weight. The model with all mothers was also adjusted for maternal pre-pregnancy BMI.

[a] Mothers with pre-pregnancy BMI 18.5-24.9 kg/m^2

[b] Mothers with pre-pregnancy BMI ≥25 kg/m^2

For peer review only

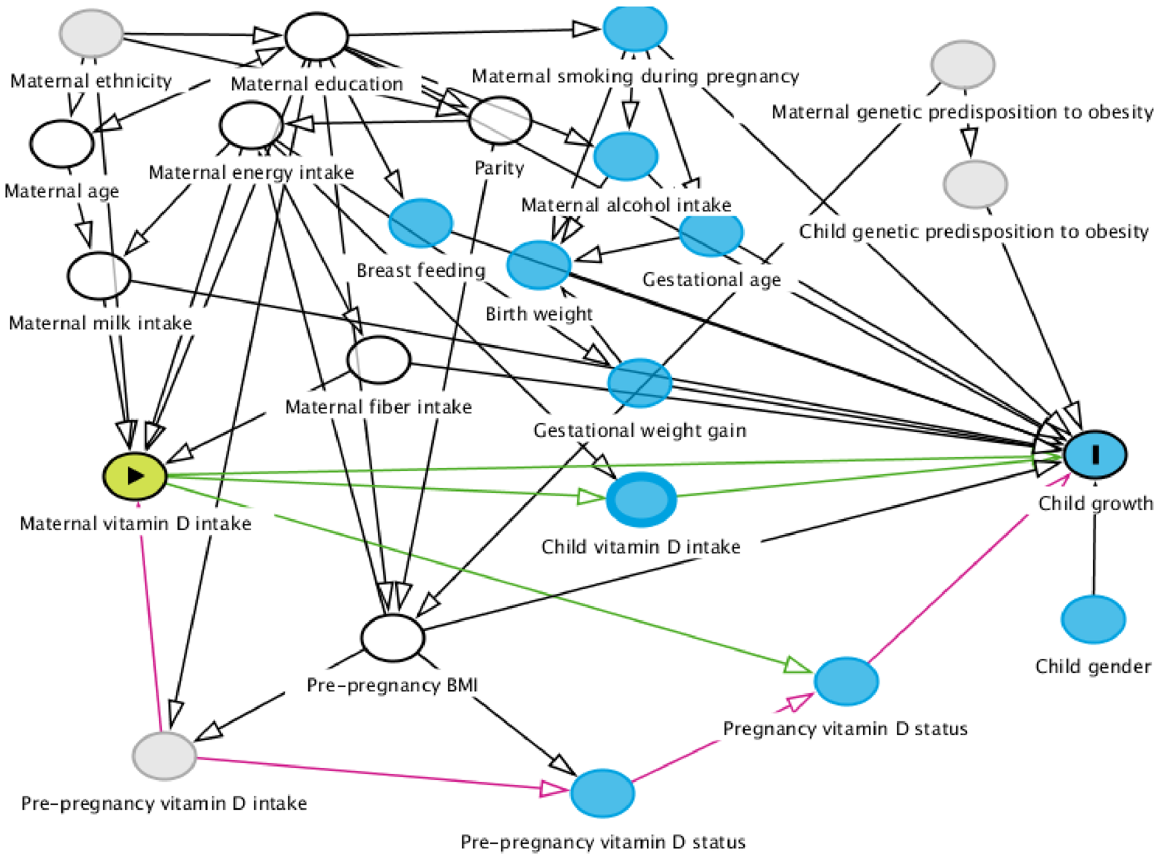

Supplementary figure 1. Directed acyclic graph on the suggested association between, exposure, outcome and covariates [a]. The graph is made using the software DAGitty[b].

Legends: White circle – ancestor of exposure and outcome, adjusted variable; blue circle – ancestor of outcome; grey circle – unobserved variable; black arrow – direct causal effects; green arrow – causal path; pink arrow – biasing path.

[a] Textor J, van der Zander B, Gilthorpe M. S, Liskiewicz M, Ellison G. T. Robust causal inference using directed acyclic graphs: the R package 'dagitty'. International Journal of Epidemiology 45(6):1887-1894, 2016.

[b] (www.dagitty.net/dags.html).

For peer review only

Supplementary table 5. Adjusted change in siblings' weights and heights from 1 month to 2 years, associated with an increase in maternal total vitamin D intake within sibships.

	Infancy			Toddlerhood		
	**1 month**	**3 months**	**6 months**	**12 months**	**18 months**	**2 years**
**Siblings in the higher maternal vitamin D category**	**Beta (95% CI)**	**Beta (95% CI)**	**Beta (95% CI)**	**Beta (95% CI)**	**Beta (95% CI)**	**Beta (95% CI)**
Weight (g)						
All mothers (n=1216)	22.0 (-28.0, 71.9)	20.4 (-30.0, 70.9)	18.2 (-34.3, 70.7)	13.7 (-46.6, 74.0)	9.2 (-62.5, 80.8)	4.6 (-80.6, 89.8)
Mothers with normal weight [a] (n=837)	3.2 (-56.6, 63.0)	2.0 (-58.2, 62.2)	0.3 (-61.7, 62,2)	-3.3 (-72.8, 66.2)	-6.8 (-87.8, 74.2)	-10.4 (-105, 84.6)
Mothers with overweight [b] (n=344)	31.3 (-57.7, 120)	29.6 (-59.9, 119)	27.0 (-65.1, 119)	21.9 (-81.4, 125)	16.9 (-104, 137)	11.8 (129, 153)
Height (cm)						
All mothers (n=1216)	0.07 (-0.15, 0.29)	0.07 (-0.14, 0.29)	0.07 (-0.14, 0.29)	0.08 (-0.12, 0.28)	0.08 (-0.11, 0.27)	0.08 (-0.11, 0.27)
Mothers with normal weight [a] (n=837)	0.02 (-0.22, 0.27)	0.02 (-0.22, 0.27)	0.03 (-0.21, 0.26)	0.04 (-0.19, 0.26)	0.04 (-0.17, 0.26)	0.05 (-0.16, 0.26)
Mothers with overweight [b] (n=344)	0.11 (-0.26, 0.47)	0.10 (-0.25, 0.46)	0.10 (-0.25, 0.45)	0.09 (-0.24, 0.42)	0.08 (-0.24, 0.40)	0.07 (-0.24, 0.39)

Beta=Beta coefficient, CI=Confidence interval

Effect estimates derived by multilevel mixed effects linear regression model, adjusted for maternal education, parity, maternal milk and yoghurt intake, maternal fiber intake, child´s gender and birth weight. The model with all mothers was also adjusted for maternal pre-pregnancy BMI. The siblings in the lower maternal vitamin D category was used as reference in separate analyses of overall (n=1211), mothers with normal weight (n=845) and with overweight (n=320).

[a] Mothers with pre-pregnancy BMI 18.5-24.9 kg/m^2

[b] Mothers with pre-pregnancy BMI ≥25 kg/m^2

For peer review only

Supplementary table 6. Adjusted changes in siblings' weights and heights from 3 years to 8 years of age, associated with an increase in maternal vitamin D intake within sibships.

	Preschool age		School age
	**3 years**	**5 years**	**8 years**
**Sibling in the higher maternal vitamin D category**	**Beta (95% CI)**	**Beta (95% CI)**	**Beta (95% CI)**
Weight (in g)			
All mothers (n=1216)	-4.4 (-120, 111)	-22.5 (-204, 159)	-49.6 (-334, 235)
Mothers with normal weight [a] (n=837)	-17.5 (-145, 110)	-31.7 (-230, 166)	-53.0 (-363, 257)
Mothers with overweight [b] (n=344)	1.6 (-187, 191)	-18.7 (-313, 276)	-49.2 (-510, 411)
Height (in cm)			
All mothers (n=1216)	0.09 (-0.10, 0.26)	0.10 (-0.12, 0.31)	0.11 (-0.20, 0.42)
Mothers with normal weight [a] (n=837)	0.07 (-0.14, 0.28)	0.10 (-0.14, 0.34)	0.15 (-0.19, 0.49)
Mothers with overweight [b] (n=344)	0.05 (-0.26, 0.36)	0.01 (-0.34, 0.37)	-0.04 (-0.55, 0.46)

Beta=Beta coefficient, CI=Confidence interval

Effect estimates derived by multilevel mixed effects linear regression model, adjusted for maternal education, parity, maternal milk and yoghurt intake, maternal fiber intake, child´s gender and birth weight. The model with all mothers was also adjusted for maternal pre-pregnancy BMI. The siblings in the lower maternal vitamin D category was used as reference in separate analyses of overall (n=1211), mothers with normal weight (n=845) and with overweight (n=320).

[a] Mothers with pre-pregnancy BMI 18.5-24.9 kg/m^2

[b] Mothers with pre-pregnancy BMI ≥25 kg/m^2

For peer review only

Supplementary table 7. Adjusted changes in siblings' risk of overweight at 3, 5 and 8 years, associated with an increase in maternal total vitamin D intake within sibships.

	**Risk of childhood overweight [a]**
	**OR (95% CI)**
**Sibling in the higher maternal vitamin D category**	
All mothers (n=1216)	
3 years	0.97 (0.75, 1.26)
5 years	0.92 (0.76, 1.12)
8 years	1.03 (0.80, 1.32)
Mothers with normal weight [b] (n=837)	
3 years	0.89 (0.62, 1.28)
5 years	0.92 (0.71, 1.20)
8 years	0.99 (0.70, 1.39)
Mothers with overweight [c] (n=344)	
3 years	1.04 (0.69, 1.57)
5 years	0.90 (0.64, 1.24)
8 years	1.03 (0.68, 1.57)

OR=Odds Ratio, CI=Confidence interval

Effect estimates derived by multilevel mixed effects linear regression model, adjusted for maternal education, parity, maternal milk and yoghurt intake, maternal fiber intake, child´s gender and birth weight. The model with all mothers was also adjusted for maternal pre-pregnancy BMI. The siblings in the lower maternal vitamin D category was used as reference in separate analyses of overall (n=1211), mothers with normal weight (n=845) and with overweight (n=320).

[a] Overweight, including obesity, defined by Cole TJ, Lobstein T. Extended international (IOTF) body mass index cut-offs for thinness, overweight and obesity. Pediatr Obes. 2012;7(4):284-294. doi:10.1111/j.2047-6310.2012.00064.x

[b] Mothers with pre-pregnancy BMI 18.5-24.9 kg/m^2

[c] Mothers with pre-pregnancy BMI ≥25 kg/m^2

For peer review only

Supplementary table 8. Associations between total maternal vitamin D intake and predicted weight growth velocities and height growth velocities from 1 month to 2 years of age.

	**Infancy**			**Toddlerhood**		
**Maternal vitamin D intake**	**1 month**	**3 months**	**6 months**	**12 months**	**18 months**	**2 years**
	**Beta (95% CI)**	**Beta (95% CI)**	**Beta (95% CI)**	**Beta (95% CI)**	**Beta (95% CI)**	**Beta (95% CI)**
All mothers n=66 840						
Weight growth velocities (g/month)						
<5 µg						
5-9.9 µg	-0.13 (-2.11, 1.86)	-0.11 (-2.04, 1.81)	-0.10 (-1.94, 1.75)	-0.06 (-1.75, 1.63)	-0.03 (-1.60, 1.54)	0.003 (-1.48, 1.49)
10-15 µg	-0.51 (-2.96, 1.94)	-0.52 (-2.89, 1.86)	-0.53 (-2.80, 1.74)	-0.55 (-2.63, 1.53)	-0.57 (-2.51, 1.36)	-0.60 (-2.43, 1.23)
>15 µg	-0.25 (-2.70, 2.20)	-0.24 (-2.62, 2.14)	-0.24 (-2.51, 2.04)	-0.22 (-2.31, 1.87)	-0.20 (-2.14, 1.73)	-0.19 (-2.03, 1.65)
Height growth velocities (cm/month)						
<5 µg						
5-9.9 µg	4x10^{-5} (-0.004, 0.004)	4x10^{-5} (-0.004, 0.004)	3x10^{-5} (-0.004, 0.004)	3x10^{-5} (-0.004, 0.004)	2x10^{-5} (-0.003, 0.003)	2x10^{-5} (-0.003, 0.003)
10-15 µg	7x10^{-5} (-0.005, 0.005)	3x10^{-5} (-0.005, 0.005)	-3x10^{-5} (-0.005, 0.005)	-2x10^{-4} (-0.005, 0.004)	-3x10^{-4} (-0.004, 0.004)	-4x10^{-4} (-0.004, 0.003)
>15 µg	7x10^{-4} (-0.004, 0.006)	7x10^{-4} (-0.004, 0.006)	7x10^{-4} (-0.004, 0.005)	6x10^{-4} (-0.004, 0.005)	5x10^{-4} (-0.004, 0.005)	5x10^{-4} (-0.003, 0.004)
Mothers with normal weight [a] n=41 970						
Weight growth velocities (g/month)						
<5 µg						
5-9.9 µg	-1.03 (-3.28, 1.22)	-1.00 (-3.18, 1.18)	-0.96 (-3.04, 1.12)	-0.88 (-2.79, 1.02)	-0.80 (-2.57, 0.96)	-0.73 (-2.40, 0.95)
10-15 µg	-1.97 (-4.69, 0.75)	-1.95 (-4.59, 0.69)	-1.92 (-4.44, 0.60)	-1.86 (-4.17, 0.45)	-1.80 (-3.94, 0.35)	-1.74 (-3.77, 0.29)
>15 µg	-1.17 (-3.86, 1.53)	-1.14 (-3.76, 1.47)	-1.11 (-3.60, 1.38)	-1.05 (-3.34, 1.24)	-0.99 (-3.11, 1.13)	-0.93 (-2.94, 1.08)
Height growth velocities (cm/month)						
<5 µg						
5-9.9 µg	-8x10^{-5} (-0.005, 0.005)	-8x10^{-5} (-0.005, 0.005)	-8x10^{-5} (-0.004, 0.004)	-8x10^{-5} (-0.004, 0.004)	-8x10^{-5} (-0.004, 0.004)	-8x10^{-5} (-0.004, 0.003)
10-15 µg	-4x10^{-4} (-0.006, 0.005)	-5x10^{-4} (-0.006, 0.005)	-5x10^{-4} (-0.006, 0.005)	-6x10^{-4} (-0.006, 0.004)	-8x10^{-4} (-0.005, 0.004)	-9x10^{-4} (-0.005, 0.003)
>15 µg	7x10^{-4} (-0.005, 0.006)	6x10^{-4} (-0.005, 0.006)	6x10^{-4} (-0.005, 0.006)	-6x10^{-4} (-0.004, 0.005)	-5x10^{-4} (-0.004, 0.005)	-4x10^{-4} (-0.004, 0.005)

For peer review only

Mothers with overweight [b] ***n=20 080***						
Weight growth velocities (g/month)						
<5 µg						
5-9.9 µg	1.17 (-1.87, 1.22)	1.18 (-1.68, 4.03)	1.19 (-1.54, 3.92)	1.22 (-1.28, 3.72)	1.25 (-1.07, 3.56)	1.28 (-0.91, 3.47)
10-15 µg	2.23 (-1.54, 6.00)	2.20 (-1.46, 5.85)	2.15 (-1.35, 5.64)	2.05 (-1.15, 5.25)	1.95 (-1.02, 4.92)	1.85 (-0.95, 4.66)
>15 µg	0.68 (-3.29, 4.65)	0.72 (-3.13, 4.57)	0.78 (-2.90, 4.45)	0.89 (-2.48, 4.26)	1.01 (-2.11, 4.13)	1.13 (-1.83, 4.08)
Height growth velocities (cm/month)						
<5 µg						
5-9.9 µg	$-3x10^{-4}$ (-0.006, 0.006)	$-2x10^{-4}$ (-0.006, 0.006)	$-2x10^{-4}$ (-0.006, 0.006)	$-1x10^{-4}$ (-0.005, 0.005)	$4x10^{-5}$ (-0.005, 0.005)	$4x10^{-5}$ (-0.005, 0.005)
10-15 µg	$6x10^{-4}$ (-0.007, 0.008)	$6x10^{-4}$ (-0.007, 0.008)	$6x10^{-4}$ (-0.007, 0.008)	$6x10^{-4}$ (-0.006, 0.007)	$5x10^{-4}$ (-0.006, 0.007)	$6x10^{-4}$ (-0.005, 0.006)
>15 µg	$-6x10^{-4}$ (-0.009, 0.008)	$-5x10^{-4}$ (-0.009, 0.008)	$-4x10^{-4}$ (-0.008, 0.007)	$-3x10^{-4}$ (-0.007, 0.007)	$-1x10^{-4}$ (-0.007, 0.006)	$-5x10^{-5}$ (-0.006, 0.006)

Beta=Beta coefficient, CI=Confidence interval

Effect estimates derive from multilevel mixed effects linear regression model, adjusted for maternal education, parity, maternal milk and yoghurt intake, maternal fiber intake, child´s gender and birth weight. The model with all mothers was also adjusted for maternal pre-pregnancy BMI.

[a] Mothers with pre-pregnancy BMI 18.5-24.9 kg/m^2

[b] Mothers with pre-pregnancy BMI ≥25 kg/m^2

For peer review only

Supplementary table 9. Associations between total maternal vitamin D intake and predicted weight growth velocities and height growth velocities from 3 years to 8 years of age.

	**Preschool age**		**School age**
	**3 years**	**5 years**	**8 years**
**Maternal vitamin D intake**	**Beta (95% CI)**	**Beta (95% CI)**	**Beta (95% CI)**
All mothers ***n=66 840***			
Weight growth velocities (g/month)			
<5 µg			
5-9.9 µg	0.07 (-1.39, 1.52)	0.20 (-1.71, 2.12)	0.40 (-2.80, 3.61)
10-15 µg	-0.64 (-2.44, 1.15)	-0.73 (-3.09, 1.63)	-0.86 (-4.81, 3.08)
>15 µg	-0.16 (-1.96, 1.64)	-0.10 (-2.46, 2.26)	-0.007 (-3.95, 3.93)
Height growth velocities (cm/month)			
<5 µg			
5-9.9 µg	$4x10^{-6}$ (-0.003, 0.003)	$-4x10^{-5}$ (-0.004, 0.004)	$-5x10^{-5}$ (-0.007, 0.007)
10-15 µg	$-7x10^{-4}$ (-0.004, 0.003)	-0.001 (-0.006, 0.004)	-0.002 (-0.010, 0.006)
>15 µg	$3x10^{-4}$ (-0.003, 0.004)	$9x10^{-5}$ (-0.005, 0.005)	$-3x10^{-4}$ (-0.009, 0.008)
Mothers with normal weight [a] ***n=41 970***			
Weight growth velocities (g/month)			
<5 µg			
5-9.9 µg	-0.57 (-2.21, 1.07)	-0.26 (-2.42, 1.91)	-0.21 (-3,42, 3.84)
10-15 µg	-1.61 (-3.60, 0.38)	-1.37 (-3.99, 1.25)	-1.00 (-5.39, 3.39)
>15 µg	-0.80 (-2.78, 1.17)	-0.56 (-3.15, 2.04)	-0.18 (-4.52, 4.15)
Height growth velocities (cm/month)			
<5 µg			
5-9.9 µg	$-8x10^{-5}$ (-0.004, 0.003)	$-7x10^{-5}$ (-0.005, 0.004)	$-7x10^{-5}$ (-0.008, 0.008)
10-15 µg	-0.001 (-0.005, 0.003)	-0.002 (-0.007, 0.004)	-0.002 (-0.011, 0.007)
>15 µg	$3x10^{-4}$ (-0.004, 0.004)	$1x10^{-4}$ (-0.005, 0.006)	$-2x10^{-4}$ (-0.009, 0.009)

For peer review only

**Mothers with overweight [b]**<br>**n=20 080**			
Weight growth velocities (g/month)			
<5 µg			
5-9.9 µg	1.33 (-0.81, 3.48)	1.45 (-1.39, 4.29)	1.62 (-3.14, 6.38)
10-15 µg	1.66 (-1.10, 4.41)	1.26 (-2.37, 4.89)	0.67 (-5.42, 6.77)
>15 µg	1.36 (-1.54, 4.26)	1.82 (-2.00, 5.64)	2.52 (-3.88, 8.92)
Height growth velocities (cm/month)			
<5 µg			
5-9.9 µg	2x10^{-4} (-0.004, 0.005)	5x10^{-4} (-0.005, 0.006)	0.001 (-0.009, 0.011)
10-15 µg	5x10^{-4} (-0.005, 0.006)	4x10^{-4} (-0.007, 0.008)	2x10^{-4} (-0.013, 0.013)
>15 µg	4x10^{-4} (-0.006, 0.006)	0.001 (-0.007, 0.009)	0.002 (-0.011, 0.015)

Beta=Beta coefficient, CI=Confidence interval

Effect estimates derive from multilevel mixed effects linear regression model, adjusted for maternal education, parity, maternal milk and yoghurt intake, maternal fiber intake, child´s gender and birth weight. The model with all mothers was also adjusted for maternal pre-pregnancy BMI.

[a] Mothers with pre-pregnancy BMI 18.5-24.9 kg/m^2

[b] Mothers with pre-pregnancy BMI ≥25 kg/m^2

For peer review only

Supplementary table 10. Associations (OR and 95% CI) between total maternal vitamin D intake and risk of childhood overweight at 3 years, 5 years and 8 years, using reported anthropometrics.

	**Risk of childhood overweight [a]**
**Maternal vitamin D intake**	**OR (95% CI)**
All mothers	
**3 years *(n=34 438)***	
<5 µg	
5-9.9 µg	1.00 (0.93, 1.08)
10-15 µg	0.96 (0.87, 1.06)
>15 µg	0.89 (0.81, 0.98)
**5 years (n=29 301)**	
<5 µg	
5-9.9 µg	0.97 (0.88, 1.06)
10-15 µg	0.97 (0.87, 1.08)
>15 µg	0.95 (0.85, 1.06)
**8 years (n=22 447)**	
<5 µg	
5-9.9 µg	1.10 (1.00, 1.20)
10-15 µg	1.05 (0.94, 1.17)
>15 µg	1.06 (0.95, 1.18)
Mothers with normal weight [b]	
**3 years *(n=22 863)***	
<5 µg	
5-9.9 µg	0.98 (0.89, 1.08)
10-15 µg	0.95 (0.84, 1.07)
>15 µg	0.87 (0.77, 0.98)
**5 years (n=19 767)**	
<5 µg	
5-9.9 µg	0.98 (0.87, 1.10)
10-15 µg	0.94 (0.81, 1.08)
>15 µg	0.90 (0.78, 1.04)
**8 years (n=15 171)**	
<5 µg	
5-9.9 µg	1.03 (0.91, 1.16)
10-15 µg	0.96 (0.83, 1.11)
>15 µg	1.02 (0.89, 1.18)
Mothers with overweight [c]	
**3 years (n=10 633)**	
<5 µg	
5-9.9 µg	1.02 (0.90, 1.15)
10-15 µg	0.96 (0.82, 1.13)
>15 µg	0.88 (0.74, 1.04)
**5 years (n=8709)**	
<5 µg	
5-9.9 µg	0.94 (0.82, 1.08)

For peer review only

10-15 µg	1.02 (0.86, 1.21)
>15 µg	0.98 (0.82, 1.17)
**8 years (n=6661)**	
<5 µg	
5-9.9 µg	1.22 (1.06, 1.40)
10-15 µg	1.19 (1.00, 1.42)
>15 µg	1.11 (0.93, 1.33)

OR=Odds Ratio, CI=Confidence interval

Effect estimates derived by logistic regression, adjusted for maternal education, parity, maternal milk and yoghurt intake, maternal fiber intake and child´s gender. The model with all mothers was also adjusted for maternal pre-pregnancy BMI.

[a] Overweight, including obesity, defined by Cole TJ, Lobstein T. Extended international (IOTF) body mass index cut-offs for thinness, overweight and obesity. Pediatr Obes. 2012;7(4):284-294. doi:10.1111/j.2047-6310.2012.00064.x

[b] Mothers with pre-pregnancy BMI 18.5-24.9 kg/m^2

[c] Mothers with pre-pregnancy BMI ≥25 kg/m^2

For peer review only

For peer review only

Reporting checklist for cohort study.

Based on the STROBE cohort guidelines.

Instructions to authors

Complete this checklist by entering the page numbers from your manuscript where readers will find each of the items listed below.

Your article may not currently address all the items on the checklist. Please modify your text to include the missing information. If you are certain that an item does not apply, please write "n/a" and provide a short explanation.

Upload your completed checklist as an extra file when you submit to a journal.

In your methods section, say that you used the STROBE cohortreporting guidelines, and cite them as:

von Elm E, Altman DG, Egger M, Pocock SJ, Gotzsche PC, Vandenbroucke JP. The Strengthening the Reporting of Observational Studies in Epidemiology (STROBE) Statement: guidelines for reporting observational studies.

For peer review only

		Reporting Item	Page Number
**Title and abstract**			
Title	#1a	Indicate the study's design with a commonly used term in the title or the abstract	2
Abstract	#1b	Provide in the abstract an informative and balanced summary of what was done and what was found	2

Introduction

Background / rationale	#2	Explain the scientific background and rationale for the investigation being reported	4-5
Objectives	#3	State specific objectives, including any prespecified hypotheses	5

Methods

Study design	#4	Present key elements of study design early in the paper	7-8
Setting	#5	Describe the setting, locations, and relevant dates, including periods of recruitment, exposure, follow-up, and data collection	5-6
Eligibility criteria	#6a	Give the eligibility criteria, and the sources and methods of selection of participants. Describe methods of follow-up.	6
Eligibility criteria	#6b	For matched studies, give matching criteria and number of exposed and unexposed	n/a
Variables	#7	Clearly define all outcomes, exposures, predictors, potential confounders, and effect modifiers. Give diagnostic criteria, if applicable	6-8
Data sources / measurement	#8	For each variable of interest give sources of data and details of methods of assessment (measurement). Describe comparability of	6-8

For peer review only

		assessment methods if there is more than one group. Give information separately for for exposed and unexposed groups if applicable.	
Bias	#9	Describe any efforts to address potential sources of bias	7
Study size	#10	Explain how the study size was arrived at	6
Quantitative variables	#11	Explain how quantitative variables were handled in the analyses. If applicable, describe which groupings were chosen, and why	7-9
Statistical methods	#12a	Describe all statistical methods, including those used to control for confounding	
8-9			
Statistical methods	#12b	Describe any methods used to examine subgroups and interactions	9
Statistical methods	#12c	Explain how missing data were addressed	6
Statistical methods	#12d	If applicable, explain how loss to follow-up was addressed	7
Statistical methods	#12e	Describe any sensitivity analyses	
9			

Results

For peer review only

Participants	#13a	Report numbers of individuals at each stage of study—eg numbers potentially eligible, examined for eligibility, confirmed eligible, included in the study, completing follow-up, and analysed. Give information separately for for exposed and unexposed groups if applicable.	6+10
Participants	#13b	Give reasons for non-participation at each stage	n/a
Participants	#13c	Consider use of a flow diagram	

n/a

Descriptive data	#14a	Give characteristics of study participants (eg demographic, clinical, social) and information on exposures and potential confounders. Give information separately for exposed and unexposed groups if applicable.	10-11
Descriptive data	#14b	Indicate number of participants with missing data for each variable of interest	

n/a

Descriptive data	#14c	Summarise follow-up time (eg, average and total amount)	

Supplementary table 1

For peer review only

Outcome data	#15	Report numbers of outcome events or summary measures over time. Give information separately for exposed and unexposed groups if applicable.	
19			
Main results	#16a	Give unadjusted estimates and, if applicable, confounder-adjusted estimates and their precision (eg, 95% confidence interval). Make clear which confounders were adjusted for and why they were included	14-20
Main results	#16b	Report category boundaries when continuous variables were categorized	14-20
Main results	#16c	If relevant, consider translating estimates of relative risk into absolute risk for a meaningful time period	
n/a			
Other analyses	#17	Report other analyses done—eg analyses of subgroups and interactions, and sensitivity analyses	12-13, 18-19, Supplementary table 10

Discussion

Key results	#18	Summarise key results with reference to study objectives	20-21

For peer review only

Limitations	#19	Discuss limitations of the study, taking into account sources of potential bias or imprecision. Discuss both direction and magnitude of any potential bias.	21
Interpretation	#20	Give a cautious overall interpretation considering objectives, limitations, multiplicity of analyses, results from similar studies, and other relevant evidence.	22
Generalisability	#21	Discuss the generalisability (external validity) of the study results	22
**Other Information**			
Funding	#22	Give the source of funding and the role of the funders for the present study and, if applicable, for the original study on which the present article is based	23

None The STROBE checklist is distributed under the terms of the Creative Commons Attribution License CC-BY. This checklist can be completed online using https://www.goodreports.org/, a tool made by the EQUATOR Network in collaboration with Penelope.ai

For peer review only
